# Advances in Dissolvable Polymers and Composites for the Oil and Gas Industry

**DOI:** 10.3390/polym18172181

**Published:** 2026-09-07

**Authors:** Lei Zhao, Jiaxiang Ren, Peixiang Xing, Donggang Yao, Meng Lu, Peng Cheng

**Affiliations:** 1 CNPC USA Corporation, Houston, TX 77042, USA; jason.ren@cnpcusa.com (J.R.); peter.xing@cnpcusa.com (P.X.); meng.lu@cnpcusa.com (M.L.); peng.cheng@cnpcusa.com (P.C.); 2 School of Materials Science and Engineering, Georgia Institute of Technology, Atlanta, GA 30332, USA; yao@gatech.edu

**Keywords:** dissolvable, polymers, composites, HTHP, oil and gas

## Abstract

The oil and gas industry has emerged as one of the largest consumers of polymer composites, with dissolvable polymers and composites representing one of the most significant technological advancements in this sector. These materials are essential for the manufacturing of high-performance tools such as hydraulic fracturing plugs, which must withstand extreme downhole conditions—temperatures of up to 250 °C and a pressure differential of up to 150 MPa—before dissolving rapidly in wellbore fluids to facilitate continuous production. Unlike traditional dissolvable polymers from the medical or consumer industries, which lack the required thermal stability, mechanical strength, and cost-effectiveness, these advanced materials must be formulated from readily available raw materials and manufactured on an industrial scale. Over the past two decades, significant progress has been made in the design and application of polymers like poly(glycolic acid), polyurethane, polyamide, epoxy, and isocyanate ester, developed through collaborative efforts between academia and industry. This review provides a comprehensive overview of the evolution of dissolvable polymer composites, covering material design, degradation mechanisms, manufacturing processes, and field applications. It concludes with insights into future development opportunities in the field.

## 1. Introduction

Traditionally, as a heavy industrial sector with products treated as commodities, the oil and gas industry relied heavily on cost-effective metal materials, primarily carbon steel, as its backbone. However, the depletion of conventional resources has pushed the industry toward increasingly challenging and unconventional environments. These include higher temperature and pressure conditions, severe corrosion environments, hydraulic fracturing operations, and offshore applications [1]. Such demanding scenarios necessitate the use of expensive corrosion-resistant alloys (CRAs) or superalloys. This shift has created a significant opportunity for polymer materials, particularly fiber-reinforced polymer composites [2]. These composites, known for their high strength (e.g., higher-pressure ratings), corrosion resistance, lightweight properties, and comparatively lower costs than certain high-end CRAs, serve as viable alternatives to traditional materials. Consequently, the demand for high-performance composite components in downhole applications is on the rise. Numerous oilfield tool manufacturers, alongside various smaller composite manufacturing companies, now supply polymer composite components for downhole operations.

**Figure 1:**
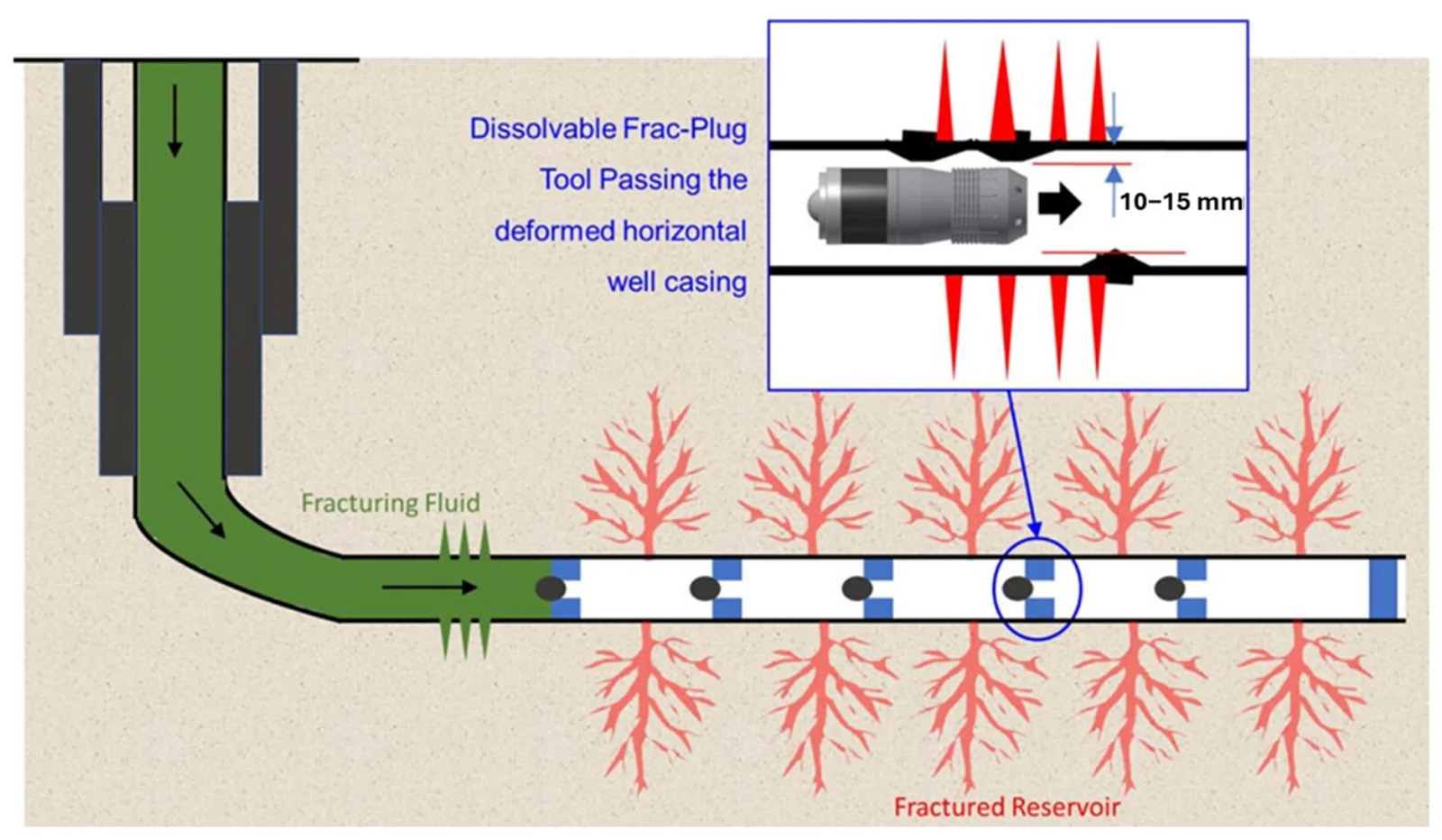
Schematic of a hydraulically fractured horizontal well [3].

A similar trend is observed with so-called “dissolvable” or “degradable” materials, a niche yet transformative innovation in the oil and gas industry. These materials, which dissolve in specific fluids after use, have significantly impacted various fields, including medical devices and sustainable materials, by enhancing functionality and reducing waste. In the upstream oil and gas sector, dissolvable materials have brought revolutionary changes, with dissolvable plugs being a prime example. Over the past two decades, unconventional resources, primarily shale oil and gas, have become a major contributor to global energy supply, marking the so-called “Energy Revolution” [3]. This revolution was made possible by hydraulic fracturing, a critical technology that transformed previously uneconomical shale oilfields with permeability orders of magnitude lower than conventional reservoirs into viable production sites. As illustrated in Figure 1, hydraulic fracturing employs an array of reciprocating positive displacement pumps to over-pressurize the reservoir near the wellbore, creating new fractures or reopening existing ones. Sand is then pumped into these fractures to establish high-permeability pathways in the shale. Enhanced permeability significantly boosts production, making previously marginal formations economically viable. During the fracturing process, zones are isolated from one another using plugs (depicted in the Figure 1 insert), which facilitate localized pressurization and are deployed downhole.

Traditional plugs were typically made of cast iron or lightweight fiberglass and required coiled tubing for milling after fracturing to re-establish communication downhole and initiate production at the wellhead [4]. A significant breakthrough came with the development of dissolvable frac plugs, which react in situ, eliminating the need for coiled tubing. This innovation not only reduced operational costs significantly but also enabled the zonal isolation of longer horizontal sections, which would otherwise be beyond the reach of coiled tubing for milling. In the early stages of dissolvable plug development, highly reactive metals like magnesium (Mg) were widely used due to their high corrosion rates in downhole environments [5]. However, after nearly a decade of widespread application, an intrinsic issue emerged: the corrosion byproduct, magnesium hydroxide (Mg(OH)_2_), has low solubility and can aggregate or recrystallize, forming hard, cement-like deposits that can completely block the well [6]. To address this problem, dissolvable polymers, particularly fiber-reinforced composites, have been developed as more effective alternatives. These materials produce byproducts that do not recrystallize, making them less likely to cause blockages [7]. High-strength dissolvable polymers and composites, such as those based on poly(glycolic acid) (PGA), cyanate esters, and epoxies, have been successfully introduced by companies like Kureha, Baker Hughes, and CNPC. Each material system offers distinct advantages and limitations, catering to different operational needs. Despite the significant progress achieved, challenges remain, and there is ample room for further innovation to enhance performance, reduce costs, and improve the overall efficiency of hydraulic fracturing operations.

There has been confusion and interchangeability in the use of the terms “dissolvable,” “degradable,” and “hydrolyzable” in the oil and gas industry, although these terms describe scientifically distinct processes. Dissolution is a physical process in which polymer chains become solvated and dispersed in a solvent without necessarily undergoing covalent bond cleavage. In contrast, degradation involves chemical changes to the polymer structure, such as main-chain or crosslink cleavage, resulting in reduced molecular weight and loss of mechanical integrity. Hydrolysis is a specific degradation mechanism in which water participates in the cleavage of susceptible chemical bonds. The term “dissolvable” became established in the oil and gas industry because magnesium alloys, the first materials widely introduced for such applications, disappear through corrosion and dissolution in downhole fluids. When polymer-based alternatives were subsequently developed, the established term “dissolvable” continued to be used for the technology and commercial products, even though most of these polymers undergo hydrolytic degradation followed by fragmentation, dispersion, or dissolution of lower-molecular-weight products. Therefore, because this review primarily focuses on polymers and composites, “degradable” is used when discussing the underlying polymer mechanism, while “dissolvable” is retained in the title and where it reflects established industrial terminology or original product descriptions.

While degradable materials and composites have been extensively reviewed in the literature, most existing studies focus on applications outside of the oil and gas industry. For example, Nair et al. systematically reviewed the development of biodegradable polymeric materials for biomedical applications, covering their synthesis, degradation mechanisms, and biomedical uses [8]. Similarly, Post et al. provided an overview of the current approaches for recycling thermoset materials, the development of inherently recyclable thermosets, and the potential applications in the recyclable materials market [9]. Although the fundamental degradation mechanisms of these materials may share similarities across different industries, the materials and technologies discussed in these reviews often fail to meet the unique demands of the oil and gas sector, particularly the extreme high-temperature and high-pressure conditions. Due to the niche nature of degradable materials in oil and gas applications—an area less explored in academic research—most R&D efforts are driven by industry rather than academia. To the best of our knowledge, no comprehensive review has been published that specifically focuses on degradable materials in oil and gas applications. This work aims to fill that gap by examining industrial-grade degradable materials and technologies tailored to the oil and gas sector, with a particular emphasis on upstream operations.

This review examines the fundamentals of degradable materials specific to the oil and gas industry (Section 2), the current state of technologies for thermoplastic materials and composites (Section 3), thermoset materials and composites (Section 4), and degradable elastomer materials (Section 5), which play a critical role as sealing components for maintaining downhole pressure. The review concludes with a summary and an outlook on future developments. Unlike previous reviews, this paper adopts an industrial perspective, emphasizing technologies and materials that are cost-effective for large-scale manufacturing and those actively promoted to the market by industrial organizations. Materials with high industrial potential, even if developed by academic researchers and not yet commercialized, are also discussed. This review aims to provide valuable insights for industry professionals seeking to improve products and processes, as well as guidance for the academic community on developing technologies that are practical for real-world industrial applications. It is also intended to serve as a resource for training and advancing research in this field.

## 2. Dissolvable/Degradable Materials Fundamentals

Understanding the degradation mechanisms and fundamental properties of materials is critical for designing and successfully implementing novel materials in practical applications. Polymer composite materials, particularly degradable composites, are relatively new to the oil and gas industry, which has traditionally focused on metals. Engineers, especially during the initial stages, often rely on their prior experience with metals to design polymer composites. This approach frequently results in complete failures. The situation is further complicated by the industry’s well-established standards for metals, while standards for polymer composites—especially in upstream applications—are either scarce or nonexistent. Additionally, there is a common misconception that degradable materials, such as biodegradable polymers, can be directly applied to oil and gas environments. However, the unique demands of these applications require entirely new material designs and formulation strategies. In this section, we will thoroughly discuss the fundamental concepts related to degradable materials, including their degradation processes and mechanisms, field requirements, material composition, and design principles.

### 2.1. Polymer Degradation Process

**Figure 2:**
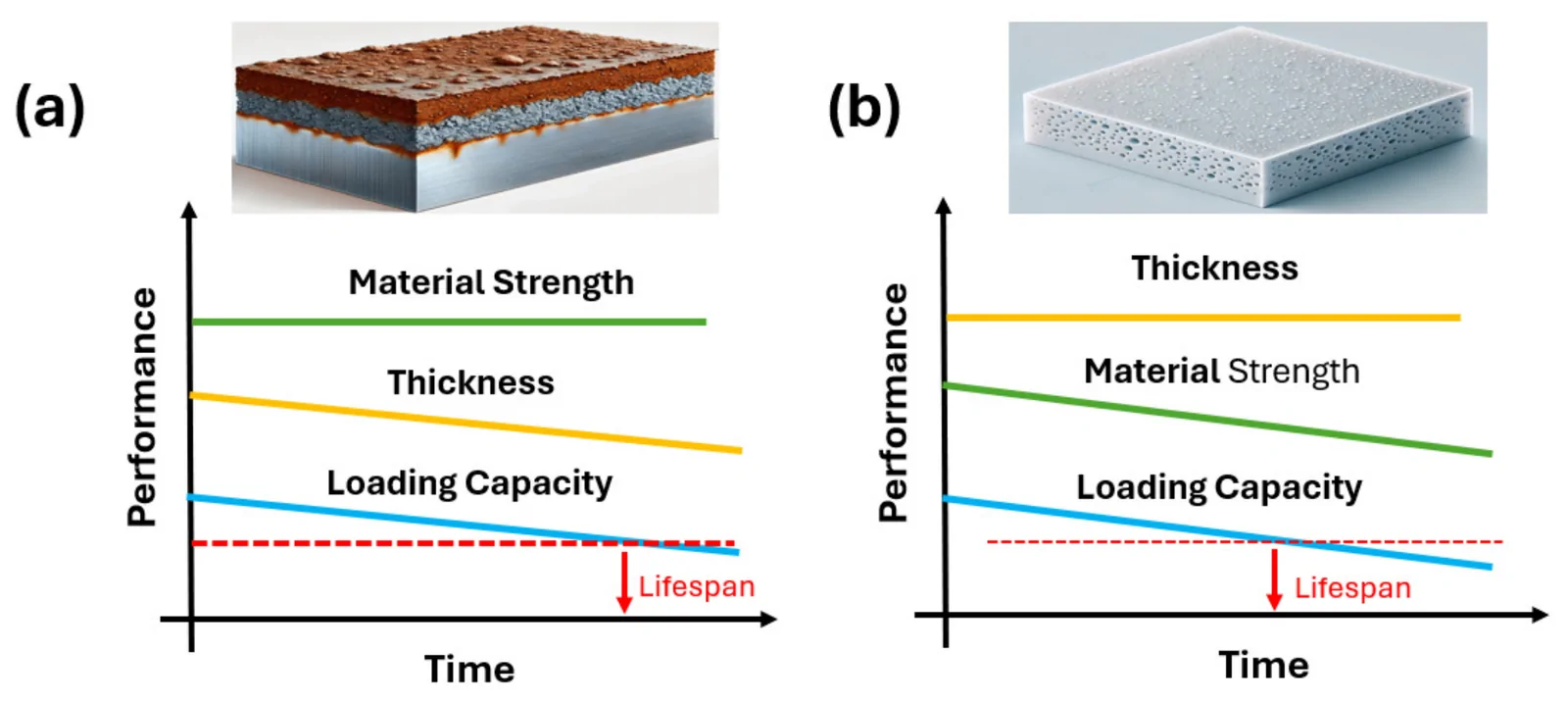
Schematic illustration of the corrosion process and lifespan assessment for metals (**a**), polymers, or fiber polymer composite (**b**).

By definition, the gradual deterioration of materials, whether metallic or non-metallic, is referred to as corrosion. However, the processes and fundamental mechanisms of material degradation differ significantly between metals and polymers. Directly applying the knowledge of metal corrosion to polymer degradation is a risky practice. In metals, the diffusion of corrosive agents—whether fluids or gases—is minimal due to the densely packed metal atoms. Consequently, corrosion typically begins at the surface and progresses by reducing the metal’s thickness, while the unaffected metal retains its mechanical properties (Figure 2a). In contrast, polymer-based materials, including fiber-reinforced composites with diverse molecular compositions and microstructures, are highly permeable to corrosive agents such as saline water, CO_2_, and H_2_S [10]. For polymers, degradation often occurs throughout the bulk material but is typically more severe near the surface due to early saturation and the transport of degradation byproducts into the surrounding fluids. As a result, these materials gradually lose mechanical strength without any measurable reduction in thickness, as shown in Figure 2b [11]. The degradation rate of polymer materials must be redefined in two aspects: the rate at which critical mechanical properties, such as strength and modulus, decline to the target level, and the rate of material loss. Notably, the loss of mechanical properties occurs much faster than the material’s physical disappearance.

In general, polymers undergo two primary types of degradation: physical degradation and chemical degradation. Physical degradation occurs when liquid molecules penetrate and diffuse between the polymer’s molecular chains, causing the material to swell [7]. This swelling is accompanied by a reduction in the polymer’s physical and mechanical properties. If the polarities of the polymer and the liquid differ, liquid absorption eventually reaches an equilibrium after a period of exposure. However, if their polarities are similar, solvation can occur [12]. In this process, liquid molecules surround the polymer chains, breaking the intermolecular bonds and leading to the physical dissolution of the polymer chains. For example, the swelling process can result in the formation or growth of voids in the polymer or contribute to the propagation of pre-existing cracks. These physical damages are irreversible. If the penetrated liquid is discharged before solvation takes place, the polymer’s physical and mechanical properties can recover to their original levels [2].

Chemical degradation in polymers occurs when materials with specific functional groups and/or bonds undergo chemical reactions under certain environmental conditions, particularly in the presence of liquids. This type of degradation involves processes such as breakage, addition, and crosslinking in the polymer chain, commonly referred to as corrosion. Examples of chemical reactions leading to degradation include oxidation, hydrolysis, transesterification, and salt formation [13]. This paper will focus primarily on hydrolytic degradation, as it is the most likely pathway for chemical degradation in downhole oil and gas applications, where the environment is rich in aqueous phases. A good selection of polymers for these applications would include those with ester bonds in the main and/or crosslinking chains, such as unsaturated polyester (UP) and epoxy resin (EP). These materials are particularly susceptible to hydrolysis under weak acid and alkaline conditions at elevated temperatures—typical downhole conditions—which leads to the scission of polymer chains.

The detailed degradation processes and mechanisms of various systems, including both thermoplastic and thermoset polymers, have been extensively studied [7,10]. These investigations primarily aim to enhance the stability and longevity of polymeric materials. However, the insights gained also benefit the development of degradable materials, as the goals and applications are fundamentally opposite. While numerous degradation mechanisms have been proposed, Tsuda has systematically categorized the general degradation processes into three distinct types [11]:

- Type 1: Surface-Reaction Type

This type occurs when corrosion products dissolve uniformly into the surrounding liquid from the material’s surface. Corrosion develops evenly across the entire surface, often involving the production of low-molecular-weight compounds from both the main polymer chains and the crosslinking structures.

- Type 2: Corrosion-Layer-Forming Type

**Figure 3:**
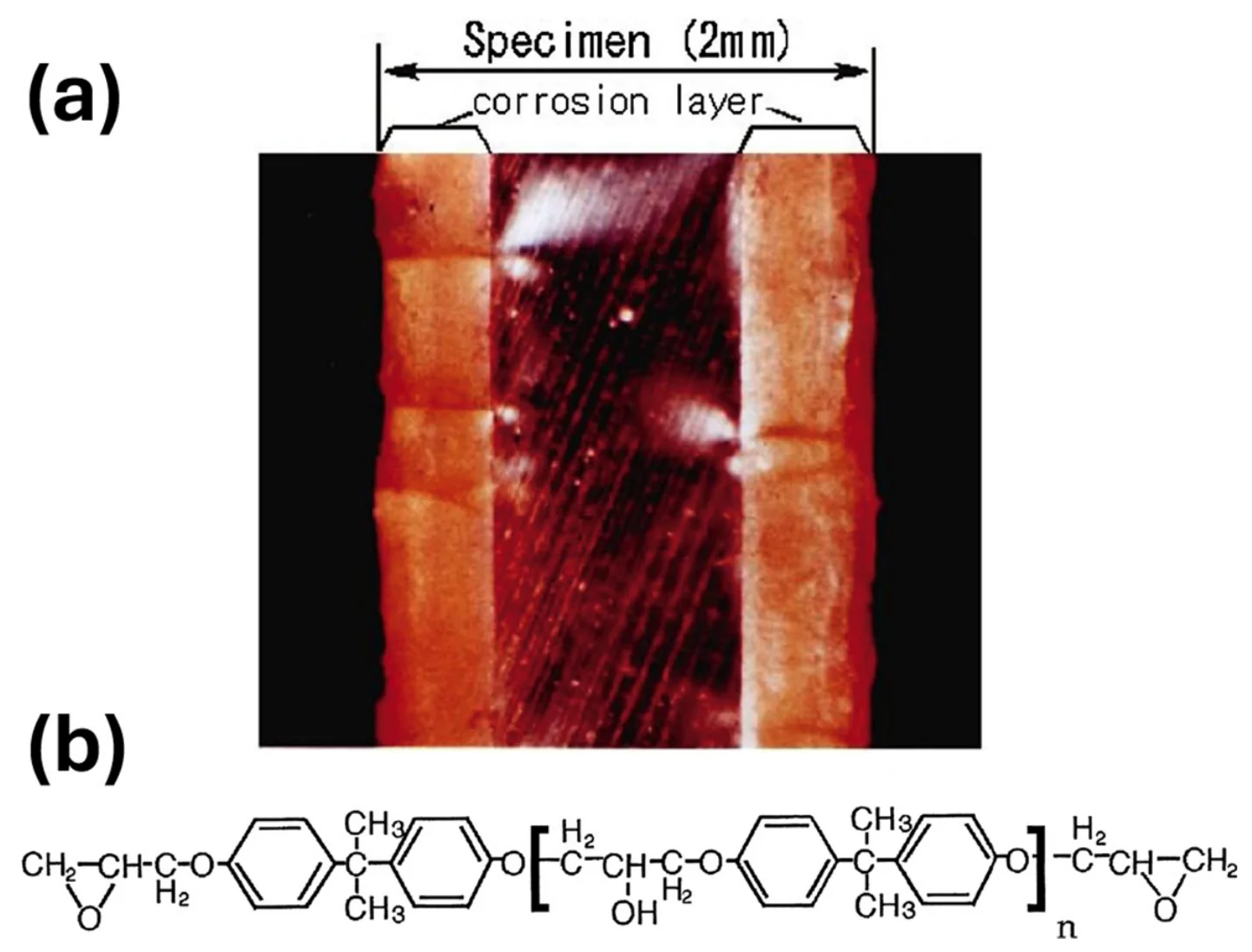
Cross-section of unsaturated specimens immersed into a 50 wt.% NaOH solution for 500 h (**a**), and chemical structure of bisphenol-A-type epoxy monomer (**b**) [11].

This type is observed when a portion of the corrosion product dissolves while the remainder forms a residual corrosion layer. A typical example (Figure 3a) involves the hydrolysis of unsaturated polyester in a NaOH system, where the ester bonds in the main chain degrade, while the styrene-based crosslinked regions remain intact due to their stable C-C bonds. The residual layer consists of high-molecular-weight chains that resist dissolution. The solubility of corrosion products depends largely on molecular weight, with the number of repeating units (n) in the main chain determining whether a surface-reaction or corrosion layer formation occurs (Figure 3b). For instance, in epoxy resin cured with phthalic anhydride, a surface reaction is observed when *n* = 0, while corrosion layer formation is noted for higher n values. This occurs due to the severe entanglement of long polymer chains, making the corrosion products less soluble.

- Type 3: Penetration Type

This type involves a two-step degradation process comprising diffusion followed by a reaction. Unlike the other types, no distinct liquid-penetrated region is observed.

These three types of corrosion are determined by the relative penetration rates of liquid by diffusion into the resin (D) and the rates of consumption of diffused liquid by chemical reaction (K). If D « K, the reactant liquid which reaches the virgin resin by diffusion is consumed immediately by a chemical reaction, and then the ‘surface-reaction type’ or ‘corrosion-layer-forming type’ occurs. The difference between these forms is caused by the solubility of the corrosion product in the liquid. If the product has high solubility, the ‘surface-reaction type’ is observed, and if it has low solubility, the ‘corrosion-layer-forming type’ is seen. If D > K, the reactant liquid penetrates the resin with a large diffusion rate, and the corrosion reaction proceeds slowly. After all reactive sites in the resin are occupied by liquid, the scission reaction occurs at all reactive sites. This behavior is the ‘penetration type’.

From the perspective of designing degradable materials, especially for applications in the oil and gas industry, the penetration-type degradation mechanism should be avoided whenever possible. The primary issue with this mechanism is its impractically slow degradation rate. More critically, the rapid penetration of solvents can cause severe physical damage to the material, such as swelling, hydrogen debonding, and other forms of structural degradation. These effects significantly compromise the mechanical strength required for the material or its components to function effectively during the operational phase, prior to the onset of degradation. In contrast, surface-reaction and corrosion-layer-forming mechanisms are preferable because the slower diffusion rate of solvents allows the material’s mechanical properties to remain largely intact until degradation is triggered. For these mechanisms, the corrosion rate is typically defined as the change in the corroded layer thickness over immersion time. By leveraging this concept and employing molecular design and formulation strategies, the residual thickness of the uncorroded material can be precisely engineered and predicted.

Among the two preferred mechanisms, surface-reaction degradation is the most advantageous. This approach produces small molecular degradation byproducts that are easily carried away by fluids, leaving minimal residuals. This characteristic is particularly beneficial for downhole applications, where large residual fragments can block wells. However, there is a trade-off between the types of degradation byproducts (or residual size) and the material’s temperature resistance. Achieving high-temperature performance typically necessitates the use of thermoset polymers with high crosslinking density, but this often results in larger residuals. To address this challenge, increasing the density of degradable functional groups—such as reducing the value of *n* in Figure 3b—can be considered. This adjustment helps lower the molecular weight of residual fragments following chain scission, thereby mitigating potential issues with large residuals. However, in practice, material selection and processing are limited by constraints such as cost, availability, and the capabilities of existing processing equipment. These limitations mean that the extent to which this strategy can be implemented is restricted, highlighting the need for further material innovation within these practical boundaries.

### 2.2. Unique Requirements from the Oil and Gas Industry

**Figure 4:**
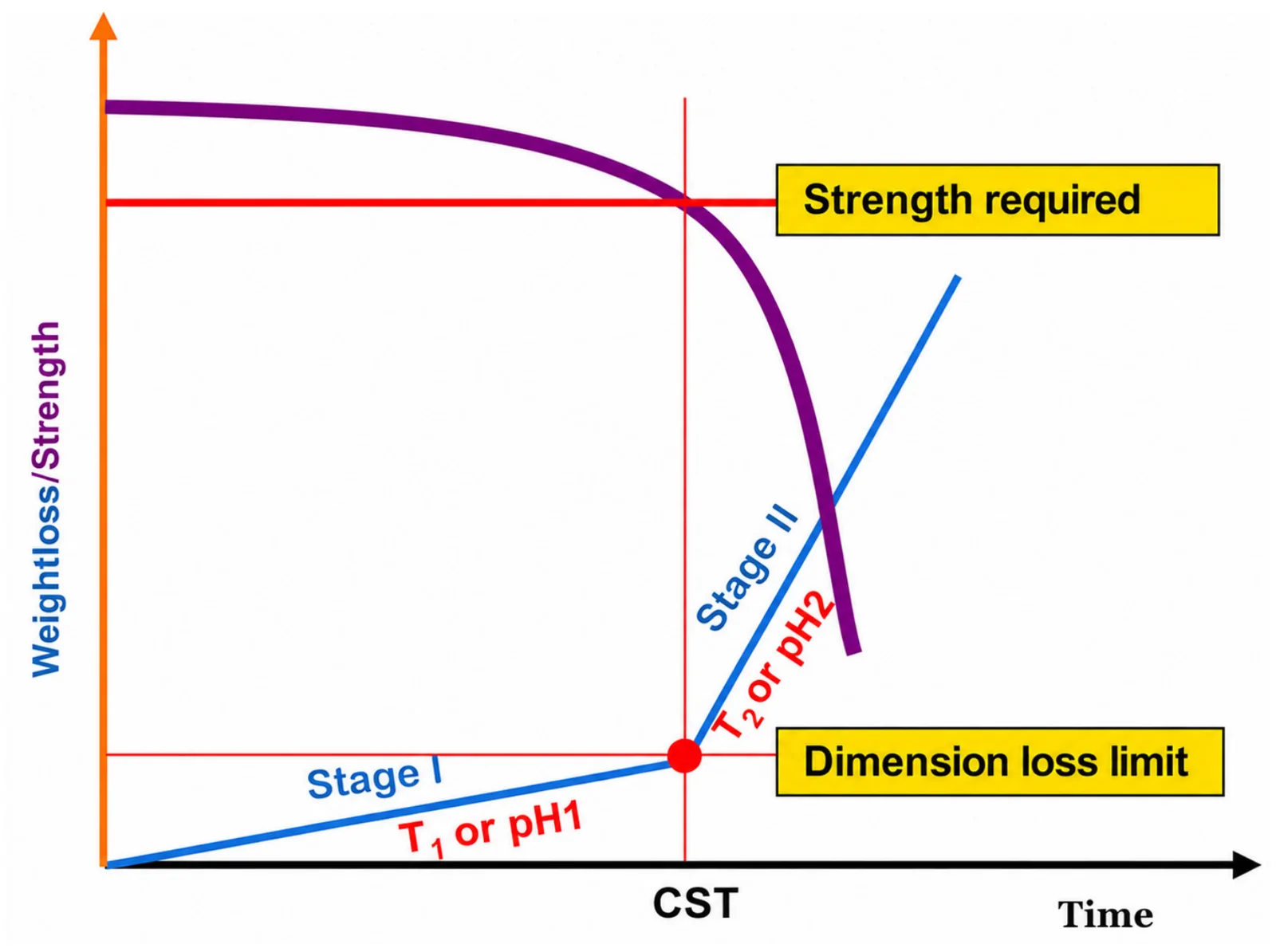
Time-dependent material properties [14].

Material design must be guided by application requirements. What makes degradable materials unique in the oil and gas industry is the combination of the harsh degradation environment and the specific physical or mechanical requirements dictated by individual applications. Degradable materials can serve various purposes; here, degradable plugs are highlighted as a primary example since they remain the most prominent use case for these advanced materials. Key property requirements for designing degradable hydraulic fracturing tools include high strength and a controlled disintegration rate. The time-dependent properties of these materials are illustrated in Figure 4. Mechanically, the material must maintain sufficient strength during the tool’s operational period, or before the critical service time (CST, typically 2 to 24 h depending on the fracturing operation and well conditions [14]), to withstand the high-pressure demands of hydraulic fracturing. Chemically, the material must resist corrosion before the CST to ensure dimensional integrity, then disintegrate rapidly afterward. The degradation process, encompassing both mechanical and dimensional loss, is highly influenced by the unique and demanding downhole corrosion environment. As the saying goes in the oilfield, “No two wells are the same”. Downhole temperatures can range from 60 °C to as high as 200 °C, pressure differentials can range from 20 MPa to 105 MPa, and fluids vary widely—from freshwater to highly saline solutions (up to 10 wt.% salt), acidic environments (up to 15% HCl), and an array of unknown chemical species. These conditions translate into stringent material requirements: exceptional mechanical strength, the ability to endure harsh conditions, and controlled dissolution across a broad temperature range. These are inherently conflicting demands, requiring a precisely engineered dissolution mechanism to achieve optimal performance. Based on the reported data, the interplay between mechanical stress and chemical degradation does not show a strong correlation in practice, despite theoretical expectations. From an engineering perspective, this interaction is not a major concern, allowing the two processes to be analyzed independently during product design and field operations. In other words, the mechanical strength loss of degradable materials can be measured and predicted separately. This enables mechanical engineers to design products, such as degradable plugs, based on these data to determine the appropriate CST.

Because degradable fracturing plugs are temporary tools and are primarily exposed to water-based treatment fluids during their critical load-bearing period, the present review emphasizes aqueous hydrolysis. Nevertheless, CO_2_ and H_2_S may influence degradation when dissolved in the aqueous phase, principally by changing fluid pH and potentially by altering fluid sorption, polymer–fiber interfaces, or polymer-specific chemical reactions. Their importance depends on gas fugacity, temperature, brine chemistry, exposure duration, and polymer composition [14]. Based on current engineering experience, temperature, water availability, pH, salinity, and catalyst formulation generally exert stronger control over the degradation time of dissolvable plugs. However, systematic high-pressure studies in CO_2_- and H_2_S-containing brines remain limited for commercial degradable-plug materials and represent an important direction for future qualification research.

So far, two mature degradable material systems have been extensively researched and have achieved commercial success: biodegradable materials and sustainable materials. While these systems have been highly referenced in R&D for oil and gas applications, they cannot be directly utilized due to the following reasons:

- Biodegradable Materials: These materials are designed to operate at low temperatures, typically below 60 °C, to align with human body compatibility or natural environmental conditions [15]. Additionally, their mechanical strength is relatively low. Common examples include thermoplastic materials such as PGA and certain thermoset materials with glass transition temperatures below 60 °C, which limit the materials’ application for high-temperature wells.
- Sustainable Materials: High-Tg thermoset materials (typically > 100 °C) are widely used in civil applications such as wind turbines, pipes, and storage tanks, often in fiber-reinforced composite forms. These materials are designed to degrade for recycling purposes, such as recovering expensive fiber reinforcements or reducing waste disposal issues. While their mechanical strength and temperature performance can meet oil and gas requirements, their degradation typically requires highly aggressive environments—such as high temperature and pressure, strong acids/bases, toxic solvents, or supercritical fluids [13,16]. These conditions are not typically present in downhole environments.

The study of prior work on dissolvable polymer composite materials highlights two primary challenges that have hindered progress in this field: (1) insufficient mechanical strength and (2) limited thermal stability at elevated temperatures. Additionally, achieving controllable degradation remains a critical area requiring improvement. From fundamental material design principles, the first step is to establish a clear connection between the desired performance criteria—such as high strength, high thermal stability, and controllable degradation—and the fundamental attributes or properties of the material. Once these links are established, suitable building blocks can be identified to create a chemical and physical structure that meets these requirements. Table 1 illustrates the mapping process from performance criteria to material properties and, ultimately, to structural design. This mapping will be elaborated on in the review of the work.

Beyond technical challenges, economic feasibility poses an equally significant, if not greater, hurdle. The pricing of degradable materials is ultimately determined by the value they add to oil and gas production, a market driven by volatile commodity prices. Dissolvable magnesium metal technology, a strong competitor, is priced around USD 20–50 per pound as of 2025. Given this competition, degradable composite materials must justify their added value; pricing above USD 100 per pound would likely render the technology unviable in the market. Therefore, degradable materials should be derived from commodity-based products or technologies. Molecular designs relying on exotic functional groups and complex synthesis processes—such as those used in the medical industry—would be impractical for this niche market. The economic feasibility is also closely tied to manufacturing scalability, as costs can be significantly reduced with increased production. While a detailed scalability analysis is beyond the scope of this review, material scientists developing new technologies for practical applications are advised to select low-cost, widely available raw materials and ensure that fabrication can be integrated into existing production lines without major modifications.

### 2.3. Degradable Polymer Matrix

Given the limited dissolvability of fiber reinforcements and the fact that the polymer matrix occupies the majority of the volume in composite materials, the degradation of the polymer matrix becomes the primary—and sometimes the only—pathway for creating a degradable composite system. Generally, there are two processes by which a material can physically disappear: (1) it can dissolve directly into the solvent (in this case, water, as relevant to our application), or (2) it can chemically degrade, breaking down large molecules or networks into smaller molecules that subsequently dissolve in water. In our application, hydrolysis is likely the primary, or even the sole, mechanism driving chemical degradation. Accordingly, this section focuses on the fundamental knowledge of dissolvable polymers and the hydrolysis process.

#### 2.3.1. Dissolvable Polymers

Water-dissolvable polymers are defined here as a class of materials that dissolve in water through a purely physical process, leaving minimal or no residue and without undergoing any chemical reactions. Examples of water-soluble polymers include polyethylene oxide (PEO), polyvinyl alcohol (PVA) and its derivatives, polyvinyl pyrrolidone (PVP), polyacrylic acid (PAA), polyacrylamide, cellulose ethers/esters (certain grades), poly-2-ethyl-2-oxazoline (PEOX), and starch and its derivatives, as well as some medium- to high-molecular-weight sugars, among others. However, a drawback of some of these polymers is their tendency to form gels and swell in water rather than fully dissolve. Among the available options, PVA, PEOX, and PEO appear to be the most promising, considering factors such as processability, cost-effectiveness, and performance. PVA is an abundant polymer widely used in water-soluble applications, including disposable medical gloves and dissolvable substrates in textile manufacturing. PEOX is a relatively new water-soluble polymer [17] that is capable of melt processing. It is marketed under the brand name Aquazol by Polymer Chemistry Innovations (PCI), Inc. (Tucson, AZ, USA). However, this amorphous polymer has a low glass transition temperature of approximately 70 °C. PEO also shows potential, but it has a low softening temperature (with a melting point of about 60 °C) and is relatively weak when processed into glue or bulk binder materials.

Based on its properties, PVA appears to be the most suitable water-soluble polymer for the development of dissolvable polymer composite materials with high mechanical performance and thermal resistance. Commercial PVA is produced through the hydrolysis of polyvinyl acetate. Fully hydrolyzed PVA has a melting point of approximately 230 °C, while partially hydrolyzed PVA has a lower melting point, typically in the range of 180–190 °C. Although the glass transition temperature (Tg) of PVA is relatively low, around 80 °C, its semicrystalline nature allows it to achieve a heat deflection temperature above 150 °C, depending on its crystallinity. However, PVA decomposes rapidly above 200 °C, necessitating the use of plasticizers to enable melt processing. The melt processing of PVA has been well documented in the literature [18,19], and certain companies, such as MonoSol LLC (Merrillville, IN, USA) and Nippon Gohsei (Osaka, Japan), have even developed injection-molding-grade PVA.

The dissolution kinetics of PVA in water depend on several factors, including the degree of hydrolysis, molecular weight, and hydrogen-bonding tendencies in aqueous solutions [20]. Fully hydrolyzed PVA can be difficult to dissolve in cold or even hot water due to strong hydrogen bonding. Partially hydrolyzed PVA, with weakened hydrogen bonding, dissolves more readily. For example, a 90% hydrolyzed PVA with a commercial molecular weight typically requires heating the PVA–water mixture to 90–100 °C to achieve complete dissolution at a 5% concentration. Higher temperatures may be required for PVA with a higher degree of hydrolysis or greater crystallinity. It is also worth noting that thermosetting polymers, due to their internal network structures, do not dissolve in solvents or form true solutions. Instead, they only swell in certain good solvents.

#### 2.3.2. Fundamentals of Hydrolysis

In conventional polymer and polymer-composite applications, hydrolysis during service is generally undesirable because most products are designed to retain their molecular integrity, dimensions, mechanical properties, and functionality over a long service life. Hydrolytic cleavage of susceptible bonds can reduce molecular weight or crosslink density and may lead to strength loss, embrittlement, swelling, cracking, delamination, or premature failure [10,11]. Degradable materials are an important exception because hydrolysis is intentionally incorporated into their design to trigger loss of mechanical integrity and subsequent disintegration after the required service period. Its mechanism has been extensively investigated under various environmental conditions to predict the material’s lifetime. An exception to this is biodegradable material, which is widely utilized in medical applications and post-consumer packaging products that eventually enter municipal waste streams. Biodegradation is, in most cases, a form of hydrolysis. As a result, biodegradable polymers typically easily incorporate hydrolysable linkages, such as esters. These polymers also tend to undergo accelerated hydrolysis in acidic or basic environments or under high processing temperatures. Compared to physical dissolution, hydrolysis can be equally or even more effective in enabling the development of dissolvable polymer composite materials. The byproducts of hydrolysis are often water-soluble, which is particularly advantageous for creating water-dissolvable polymer composite materials designed for downhole applications.

Organic materials and polymers synthesized through condensation or addition reactions using water-soluble feedstocks are typically susceptible to hydrolysis. This is particularly true for polycondensation reactions involving diacids (or triacids) and diols (or triols), where polymerization and hydrolysis compete with each other. As a result, achieving high-molecular-weight condensation polymers is often challenging. To overcome this, ring-opening anionic polymerization is frequently employed for producing high-molecular-weight polyamides and polyesters.

**Figure 5:**
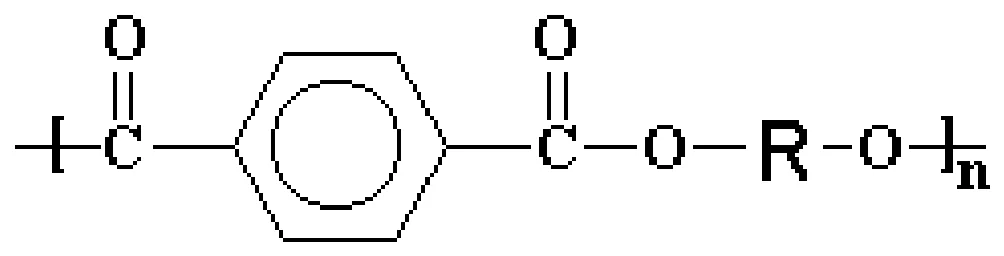
Terephthalate polyesters: polyethylene terephthalate (PET) for R = 2 (CH_2_); polytrimethylene terephthalate (PTT) for R = 3 (CH_2_); polybutylene terephthalate (PBT) for R = 4 (CH_2_).

The kinetics and rate of hydrolysis are largely influenced by processing conditions, particularly pH, temperature, hydrophobicity, morphology, degree of crystallinity, and material porosity. For polymers that degrade via bulk erosion, water uptake capacity and the water solubility of oligomers/monomers play critical roles. Polymers with high polarity and hydrophilicity are more prone to hydrolysis. For example, in the case of terephthalate esters (PET, PTT, and PBT), as illustrated in Figure 5, the expected hydrolysis rate follows the order: PET > PTT > PBT. This trend arises because PET, with its shorter aliphatic chain (containing 2 methylene groups), is less hydrophobic compared to PTT and PBT, making it more susceptible to hydrolytic degradation.

**Figure 6:**
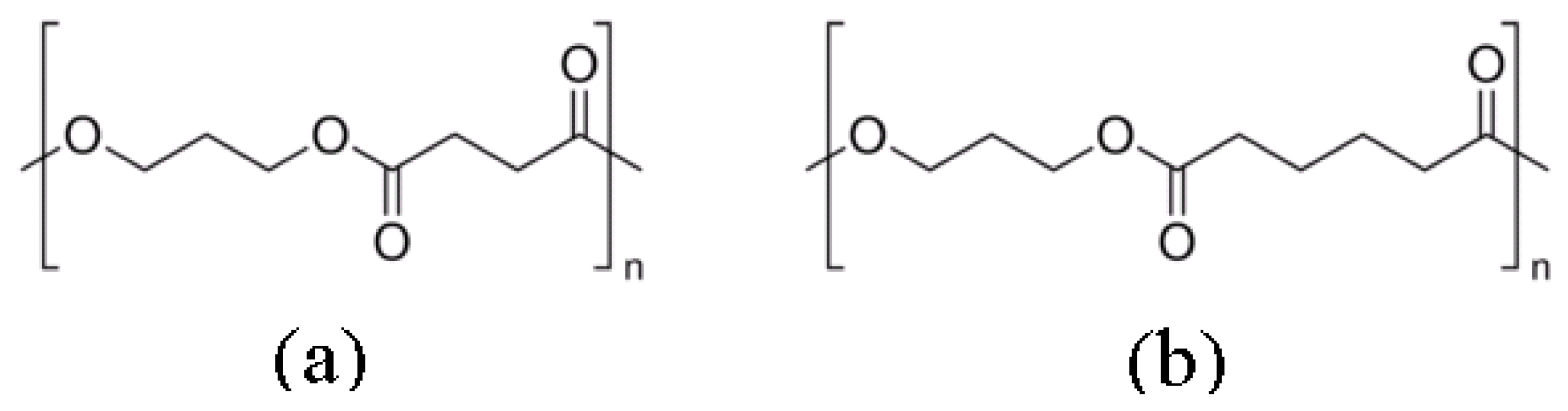
Two aliphatic polyesters: (**a**) poly(propylene succinate); (**b**) poly(propylene adipate).

For aliphatic polyesters, hydrolysis tends to increase as the length of the aliphatic segment in the polymer chain decreases. For instance, poly(propylene succinate) is expected to undergo hydrolysis more readily than poly(propylene adipate). The chemical structures of these two polymers are shown in Figure 6.

**Figure 7:**
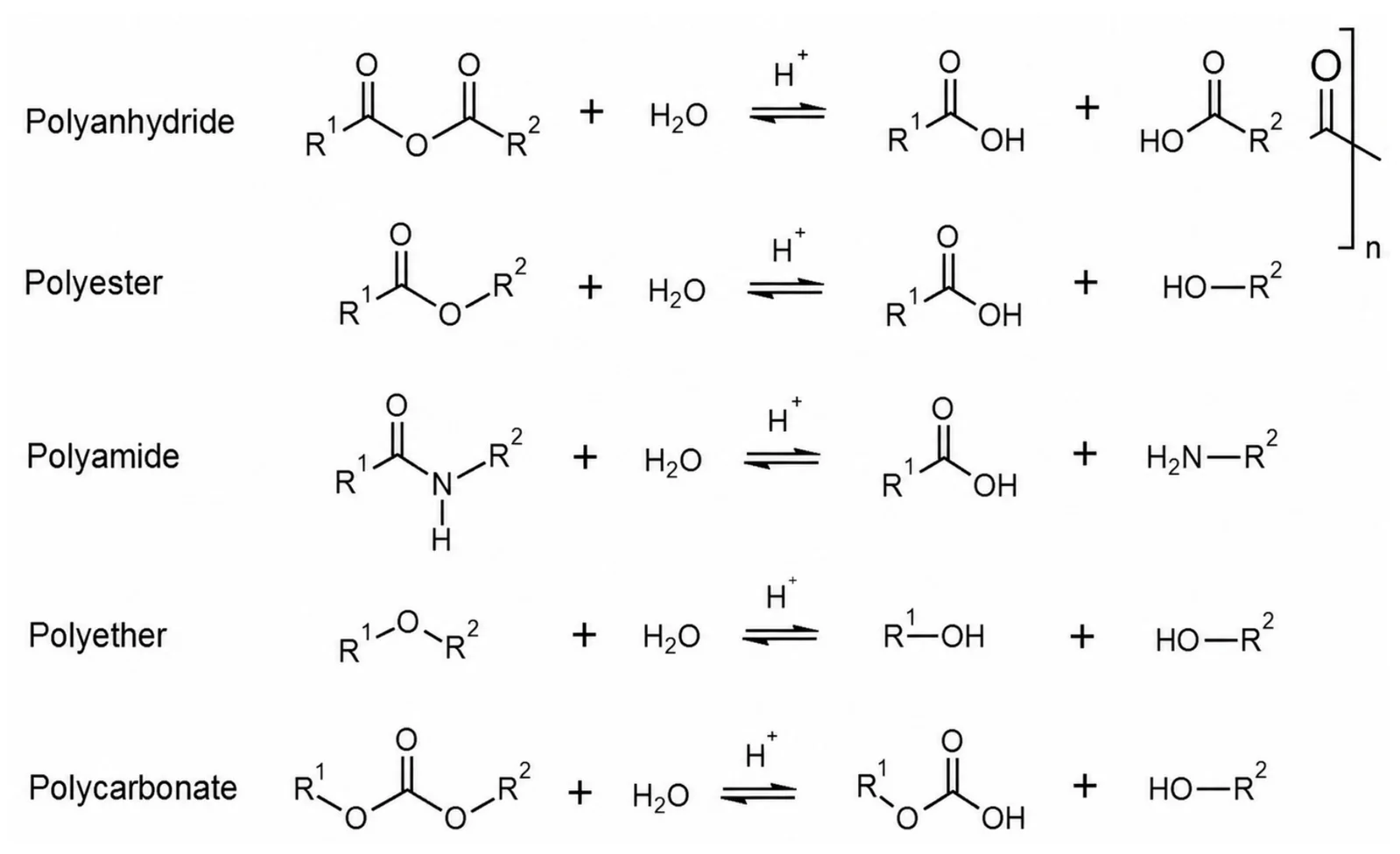
Hydrolysis of common condensation polymers [19].

In addition to polyesters, polyanhydrides, polyamides, and polyethers are commonly synthesized through addition or condensation reactions. These organic materials also typically undergo hydrolysis in aqueous environments. When the connecting midblocks are similar, the rate of hydrolysis can be qualitatively described by the following inequality: anhydride > ester > amide > ether. The basic hydrolysis reactions of these linking groups are illustrated in Figure 7 [19].

Polyesters are of great interest for applications in dissolvable polymer composites due to their widespread availability. The hydrolysis half-time in an aqueous solution is commonly modeled by [21]

$$t_{1/2}=\frac{\ln2}{K_{A}[H^{+}]+K_{N}+K_{B}[{\mathrm{OH}}^{-}]}$$

where *K_A_* is the acid-catalyzed hydrolysis rate constant, *K_N_* is the neutral hydrolysis rate constant, *K_B_* is the base-catalyzed hydrolysis rate constant, and [H^+^] and [OH^−^] are molar concentrations. For carboxylic acid esters, *K_N_* and *K_B_* increase with EWGs (electron-withdrawing groups) on R_1_ and decrease with increasing steric bulk on R_2_, referring to Table 2.

Manufacturing processes can further influence hydrolysis by changing the morphology and molecular orientation of the polymer. During injection molding, shear and elongational flow can orient macromolecular chains along the melt-flow direction, while rapid cooling near the mold wall and slower cooling in the core can produce a heterogeneous skin–core structure. Compression molding may produce less orientation than injection molding under some processing conditions, although charge flow, prior extrusion or compounding, and nonuniform cooling can still generate significant spatial variations. These processing effects can alter crystallinity, free volume, residual stress, and water-transport pathways and may consequently cause different rates of water uptake, strength loss, and disintegration in the flow, transverse, and thickness directions. This processing-induced degradation anisotropy can be particularly important in large or geometrically complex molded components. Nevertheless, based on current engineering experience with degradable oilfield tools, polymer chemistry, catalyst formulation, temperature, pH, and water availability generally exert stronger control over the overall degradation time than processing-induced orientation. Therefore, formulation remains the primary means of controlling degradation, while molding conditions and specimen orientation should be included in manufacturing control and product qualification.

### 2.4. Dissolvable/Degradable Fibers for Reinforcement

Water-degradable or dissolvable fibers hold promise for the development of dissolvable composite materials. In principle, most of the aforementioned water-soluble materials can be fabricated into fibers. However, only materials with sufficient mechanical modulus and strength are suitable as reinforcements. For instance, PLA fibers are generally weak, significantly weaker than typical nylon and PET fibers [22], making them unsuitable for further consideration. Among the reported options, PGA and PVA fibers exhibit relatively high strength. Notably, gel spinning can produce PVA fibers with strengths comparable to those of glass fibers (GFs), exceeding 3 GPa. However, commercially available PVA fibers tend to have lower strength, often below 2 GPa. Table 3 highlights the properties of Kuralon^®^ water-soluble PVA fibers, manufactured by Kuraray Co., Ltd. (Chiyoda, Japan), compared to various polymer fibers [23]. Kuralon K-II fibers achieve tensile strengths of approximately 15 cN/dtex (~1.7–1.8 GPa) for monofilaments. Similarly, PGA fibers demonstrate a strength slightly exceeding that of nylon and PET fibers, with laboratory-scale experiments achieving tensile strengths of up to 1.5 GPa. Table 4 summarizes the mechanical properties of Kuredux^®^ PGA monofilaments, produced by the Kureha Corporation [24]. In academia, Yao et al. reported a gel-spinning process for producing strong PEO fibers [25]. With appropriate process optimization, PEO fibers can achieve superior strength, stiffness, and toughness compared to nylon 6 fibers. However, this process has not yet been scaled up to pilot production. These degradable polymer fibers typically have low thermal resistance (below 100 °C), limiting their use in composites to low-temperature applications.

Water-soluble inorganic fibers could also be a potential option. However, most known water-soluble inorganic materials are either too weak or too brittle to form practical fibers. An exception worth exploring is water-soluble glass fibers. While no such product is currently available on the market, the fundamental material science of glass suggests that their development is feasible. However, given the large-scale manufacturing capabilities of fiberglass and the current market demands and price range, the industrial-scale production of water-soluble glass fibers may not yet be economically viable.

The mechanical strength of the previously discussed fibers falls significantly short of that of commercial-grade glass and carbon fibers, particularly at elevated temperatures. Given the cost considerations acceptable to the oil and gas industry, these commercial-grade fibers remain the most viable options. While continuous fibers offer superior mechanical performance, they compromise dissolvability. The excessively long fibers can hinder dissolvability by causing blockages, much like hair clogging a drain. The optimal solution, therefore, is the use of discrete glass or carbon fibers of appropriate length.

When designing high-strength composite materials, understanding the impact of fiber length and loading on mechanical properties is crucial. Longer fibers are well known to enhance toughness, strength, and stiffness, while higher fiber loading results in stiffer materials. However, both factors tend to reduce elongation. In thermoplastic processing, such as injection molding, it is common to include 30–60 wt.% glass fiber. For instance, BASF’s Ultramid^®^ nylon 66, reinforced with 60% glass fiber, achieves a tensile modulus of ~21 GPa and a strength of ~220 MPa, compared to the unreinforced nylon 66, which has a tensile strength of approximately 60 MPa. At high fiber loadings, the modulus and strength are predominantly determined by the fibers, assuming excellent fiber-to-matrix bonding. While longer fibers offer superior mechanical properties, they can complicate injection molding, necessitating alternative processing methods. A similar scenario applies to thermoset composites, where fibers of varying strengths are used to balance dissolvability and mechanical performance requirements—the details of which will be discussed later. Generally, short fibers (~1–2 mm) are typically used in thermoplastics to maintain compatibility with processing methods, while long fibers (~10–15 mm) are more suitable for thermosets, which are often produced through compression molding.

Interfacial bonding plays a crucial role in determining the mechanical properties of composite materials. If an air gap forms between the fiber and the matrix, the resulting composite will exhibit poor mechanical performance, particularly in terms of flexural strength. In aerospace engineering, epoxy is a commonly used matrix material, primarily because the industry has developed highly effective protocols for coating carbon fibers to achieve exceptional adhesion to this polymer.

### 2.5. Catalyst for Hydrolysis Process

As previously discussed, biodegradable materials generally lack the mechanical strength and temperature resistance required for demanding applications, despite their excellent degradation or dissolving capabilities. Conversely, high-strength, high-temperature materials, such as highly crosslinked thermosets, typically require exposure to corrosive fluid environments to catalyze the hydrolysis process. Such conditions are rarely present in downhole environments and cannot be feasibly introduced. The optimal solution is to incorporate catalysts directly into the polymer matrix during the manufacturing process. When the polymer is exposed to an aqueous environment, it absorbs water, causing these catalysts to dissolve or dissociate locally. This creates localized corrosive conditions that catalyze the hydrolysis process, enabling the desired dissolution rate.

The hydrolysis of ester linkages is generally used as the primary mechanism for creating water-dissolvable polymer composite materials. In this process, both acids and bases can serve as effective hydrolysis catalysts. However, several considerations must be addressed when selecting an effective and practical catalyst:

- The catalyst must not react with the polymer or prepolymer during mixing and processing.
- It must not catalyze hydrolysis during the mixing or processing stages.
- The catalyst must withstand processing conditions, including high temperatures, without degradation, vaporization, or loss of effectiveness.
- The catalyst should be water-free to avoid premature activation.

The ideal form of a catalyst is dry powder, as this allows it to be easily blended into the base resin or polymer using standard mixing operations. Many alkali metal oxides fulfill these criteria. Furthermore, these alkali metal oxides can also serve as moisture scavengers during mixing, protecting the polymer molecules from premature degradation. Acid catalysts are much less commonly used, especially in applications involving thermosets. For instance, acids typically react with epoxy—the most widely used thermoset resin for composite manufacturing—during the curing process. Additionally, the solid forms of strong acids are relatively rare.

To illustrate the catalyst selection process, the case of polyesters, specifically PET, can be used as an example. Among terephthalate polyesters, PET is the most easily degraded via hydrolysis. However, its natural hydrolysis in warm or even hot water is relatively slow. Both acids and bases are theoretically effective catalysts for PET hydrolysis, but bases are generally more favorable. The hydrophobic nature of PET plays a significant role here. While acids may theoretically catalyze hydrolysis effectively, their diffusion into PET is hindered by the material’s hydrophobicity. In contrast, bases are known to interact more effectively with hydrophobic substances. Thus, the addition of an alkali metal oxide, such as CaO, during compounding can significantly accelerate PET hydrolysis. This approach is both practical and cost-effective [27].

**Figure 8:**
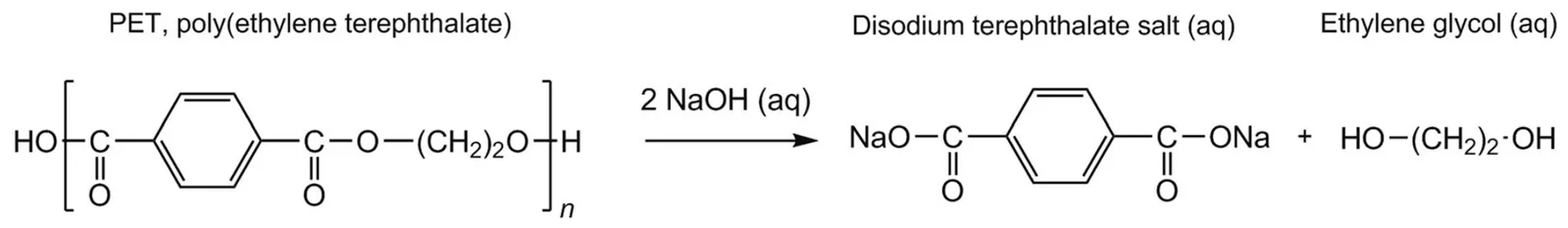
Hydrolysis of PET in NaOH basic aqueous solution [28].

In a basic NaOH solution, PET hydrolysis produces water-soluble substances, as illustrated in Figure 8. The reaction generates disodium terephthalate salt and ethylene glycol, both of which dissolve readily in water [28].

## 3. Degradable Thermoplastic Polymers

Aliphatic polyesters, as one of the most commonly used degradable thermoplastics, have garnered increasing attention from both academia and industry in recent years, particularly due to their environmental benefits [29,30]. This interest stems from their inherent susceptibility to hydrolytic degradation and biodegradation, as well as the non-toxic nature of their degradation byproducts. These polyesters are derived from three primary sources: fossil fuels, microbial fermentation, and plants. Table 5 summarizes the properties of biodegradable aliphatic polyesters as detailed in reference [31].

**Figure 9:**
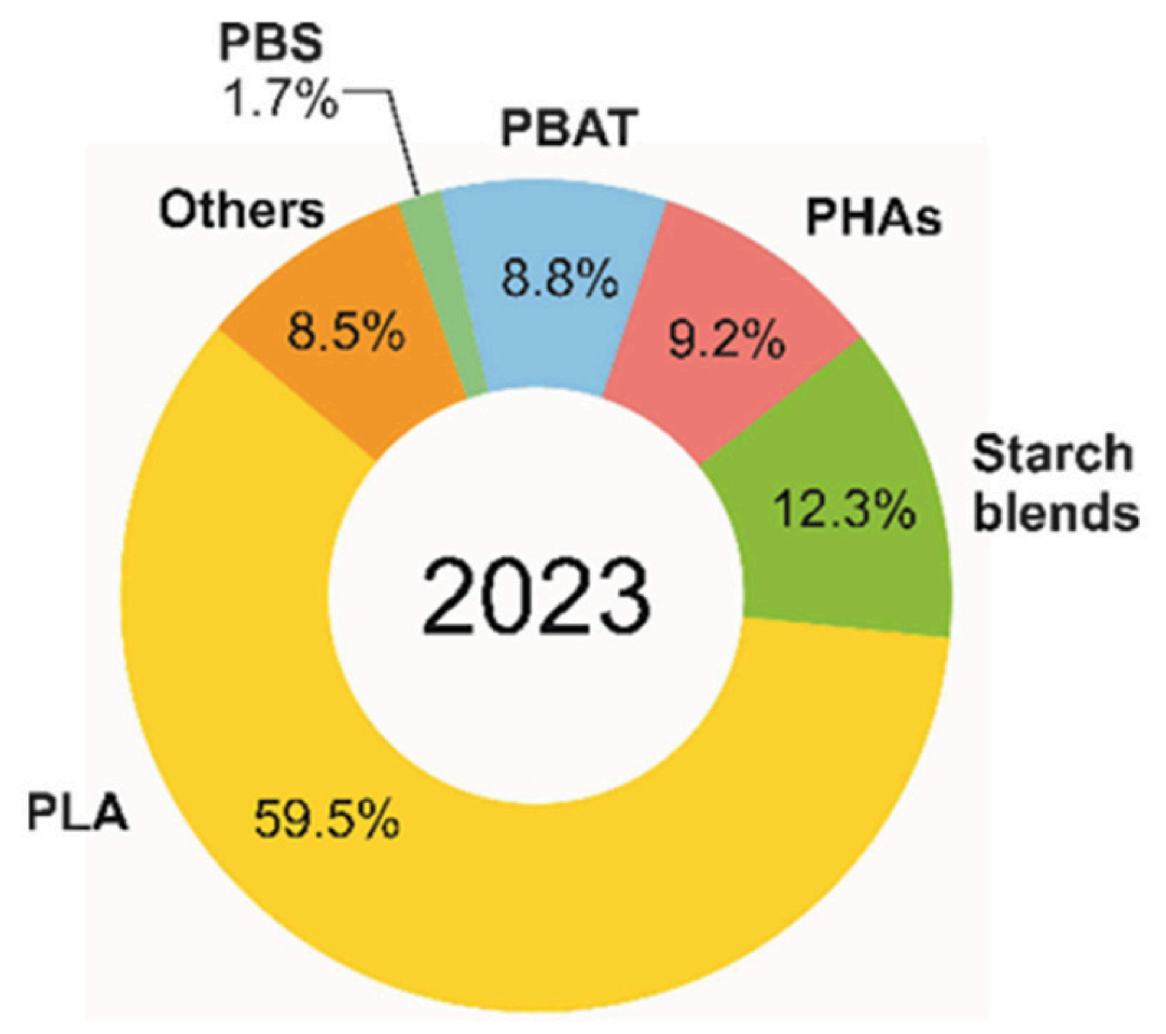
Global production capacities of biodegradable/compostable plastics by material type in 2023, adapted from the European Bioplastics Association, 2023 [30].

A common example of an aliphatic polyester produced through microbial fermentation is poly(3-hydroxybutyrate) (PHB), which is both biosynthesized and biodegradable [31]. Metabolix Inc. (now Yield10 Bioscience, Inc., Woburn, MA, USA) has developed a variety of switchgrass capable of producing substantial amounts of PHB bioplastic within its leaf tissues [32]. Due to its unique semicrystalline structure, PHB and its blends with other polymers have been extensively studied [33,34,35,36], focusing on the fundamental aspects of structure and properties. For instance, PHB has proven to be an excellent model polymer for studying crystalline nucleation, as it is free from catalyst residues and exhibits perfect tacticity [33]. Its biodegradability has led to the development of applications in packaging, medical devices, and more. Another well-known aliphatic polyester derived from plants is poly(lactic acid) (PLA). Thanks to its abundant raw material sources and favorable properties, PLA is the most widely commercialized aliphatic polyester. It is utilized in various fields, including packaging, medical applications, agriculture, and 3D printing [37]. NatureWorks LLC (Minneapolis, MN, USA) is one of the leading global manufacturers of PLA [37]. Recently, poly(butylene succinate) (PBS) has also attracted significant attention, particularly for its use in biodegradable films due to its excellent melt processability and mechanical properties. In 2023, the global production of biodegradable plastics reached approximately 1.1 million tons, with PLA accounting for around 60% of this volume, as shown in Figure 9 [30]. The production of biodegradable plastics is projected to grow by approximately 250% between 2023 and 2028.

**Figure 10:**
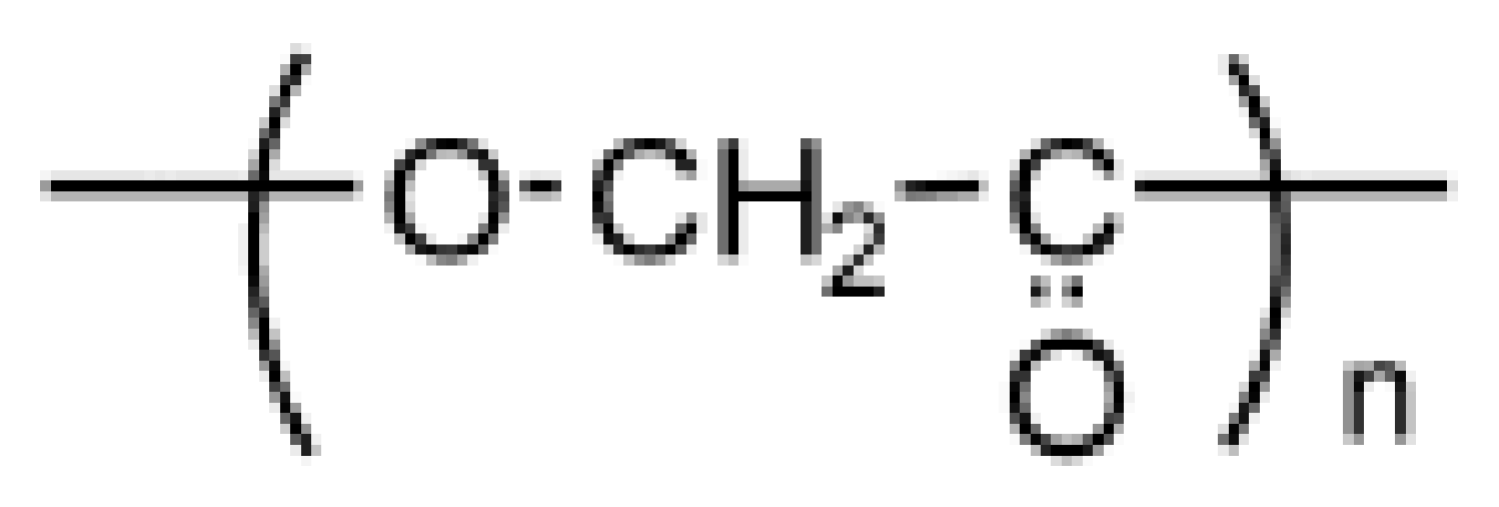
The polymer chain structure of PGA.

A typical aliphatic polyester from fossil fuel is Poly(ε-caprolactone) (PCL), which is a semi-crystalline, biodegradable polyester with a melting point of about 60 °C and a glass transition temperature of about −60 °C. PCL has been widely used in long-term implants and controlled drug release applications [29,31,38,39]. Another typical aliphatic polyester from fossil fuel is poly(glycolic acid) (PGA) (Figure 10), which is a semi-crystalline, biodegradable polyester with a melting point of around 230 °C and a glass transition temperature of about 40 °C. It can be made via polycondensation or via ring-opening polymerization, where the polycondensation method produces a low molecular weight, and the ring-opening polymerization produces a high molecular weight.

Kureha Inc. is the major manufacturer of PGA around the world [40]. PGA was marketed as a biodegradable and bioabsorbable suture thread (trade name DEXON) and widely used in medical applications. PGA sutures lose almost 50% of their strength after two weeks and 100% at four weeks, whereas PGA sutures are completely absorbed in 4–6 months. It was observed that the degree of crystallinity increased from 31% to 56% in 24 days during the significant degradation of PGA. This result validates the mechanism of hydrolytic degradation, where hydrolysis first happens in the amorphous regions over the crystalline regions. A similar degradation mechanism was also observed for biodegradable PCL [39].

**Figure 11:**
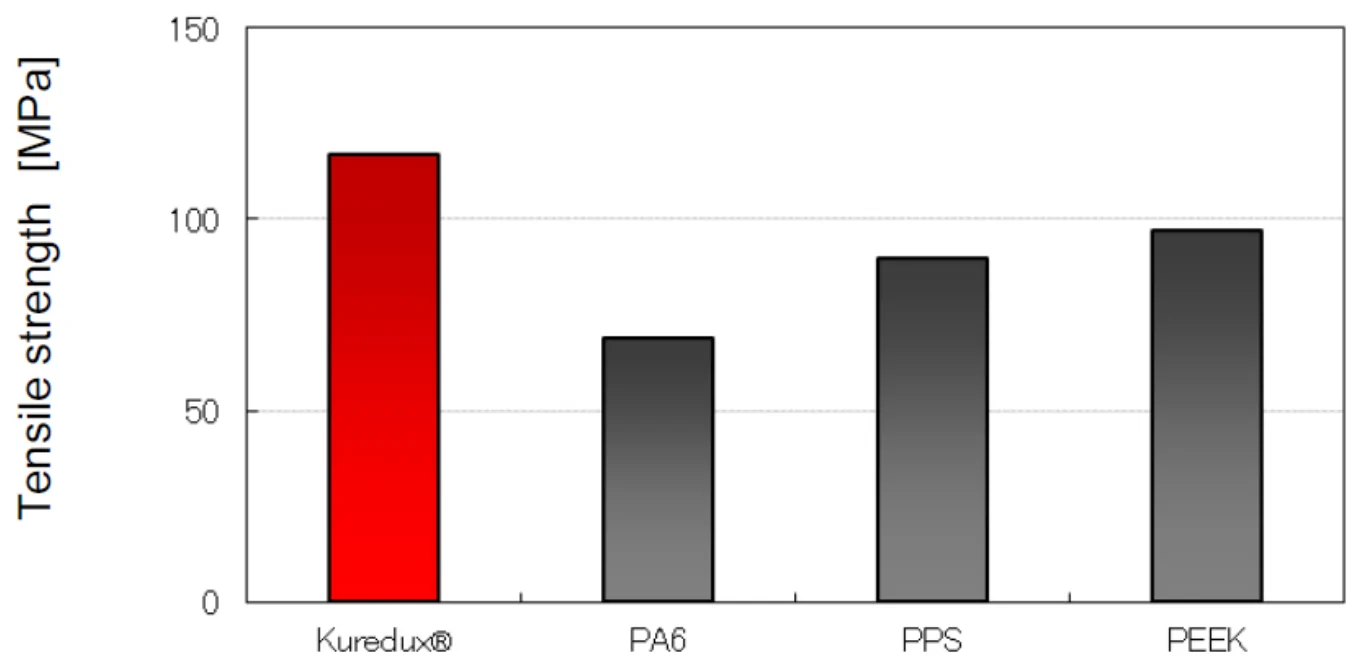
Comparison of the tensile strength of PGA and selected engineering plastics [40].

For semicrystalline PGA, however, the overall degree of crystallinity alone may not fully describe its hydrolytic behavior. Spherulite size, crystalline morphology, and processing-induced molecular orientation can influence the distribution and accessibility of crystalline and amorphous regions. Because water preferentially penetrates the more accessible amorphous regions, these morphological differences may result in spatial or directional variations in water uptake, strength loss, cracking, and disintegration. In industrial degradable-plug production, molding conditions are constrained by component geometry, fiber loading, production rate, cycle time, and cost [40,41]. Therefore, formulation and catalyst selection remain the principal practical means of adjusting degradation time, while morphology and orientation should be controlled through a defined manufacturing window and considered during component qualification. Kureha recently successfully developed an industrial production technology for high-molecular-weight PGA [41]. This mass production of high-molecular-weight PGA allows for more applications of PGA as engineering plastics with a high mechanical strength (tensile strength ~117 MPa). The mechanical properties of PGA (from Kuredux data sheet) are shown in Table 6 [40]. Its tensile strength is similar to the polyether ether ketone (PEEK) and is comparable to other engineering plastics (PPS, etc.) (Figure 11) [40]. Its flexural strength is also similar to PEEK [41].

**Figure 12:**
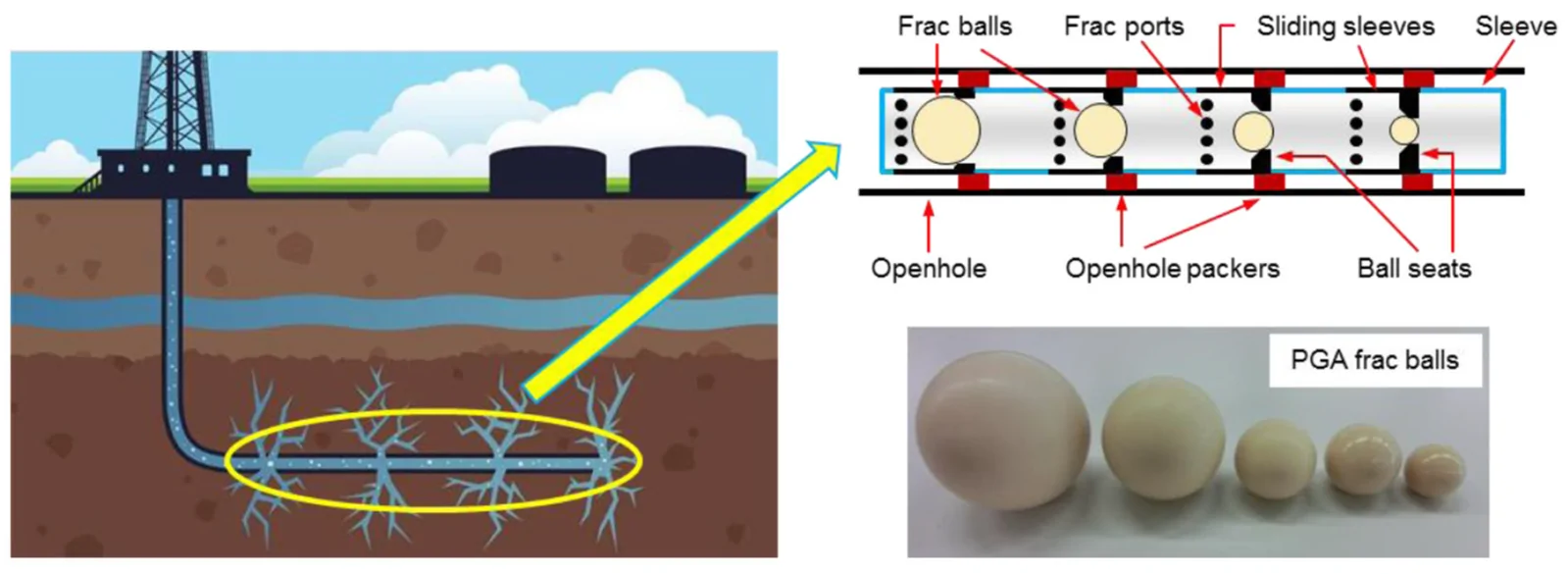
Schematic of multi-stage fracturing with PGA frac balls [49].

**Figure 13:**
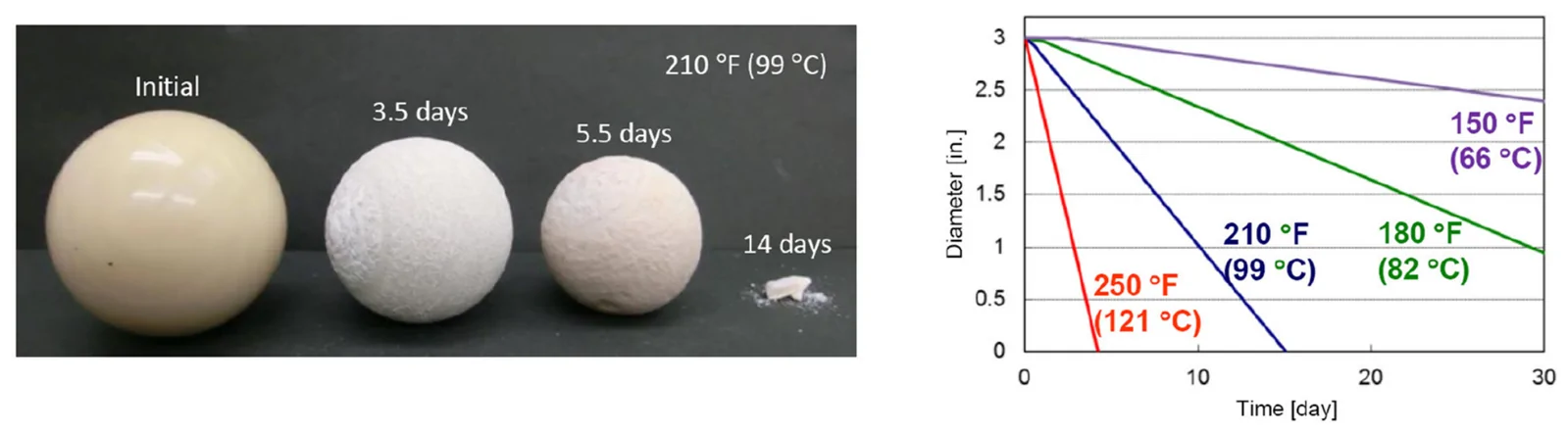
Change in the diameter of PGA frac balls in water over time [49].

The multi-stage fracturing in shale oil and gas formations has become common and popular, which also makes the application of PGA more practical due to the high mechanical properties of PGA (high molecular weight for extrusion and molding) and the mass production of PGA by Kureha [40]. Kureha Energy Solution has successfully developed frac balls and dissolvable frac plugs [49,50,51] with PGA material. The schematic of multi-stage fracturing with PGA frac balls is shown in Figure 12, in which the smallest ball is inserted first for fracturing. Then, the ball with a slightly larger diameter is inserted in the following order after each stage of fracturing. The PGA frac balls degraded and their diameter gradually reduced in water at selected downhole temperatures (Figure 13) [49].

**Figure 14:**
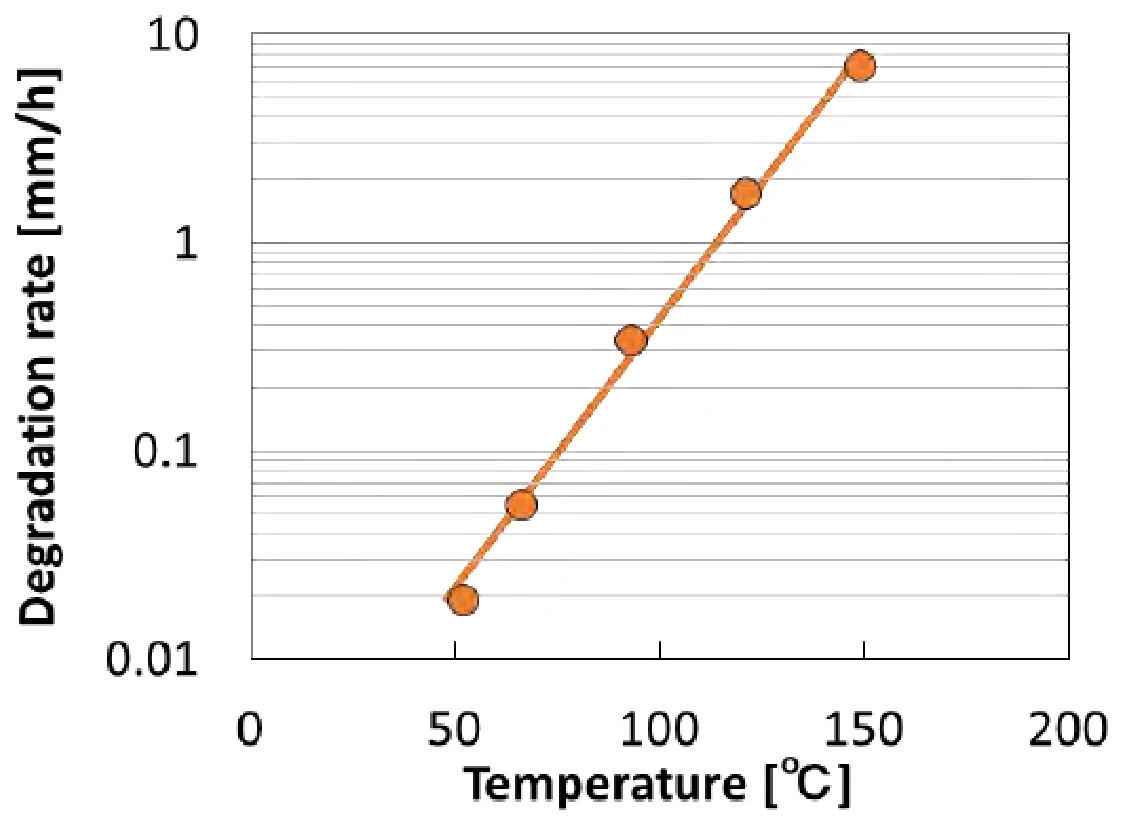
Temperature dependence of the degradation rate of PGA in 0.05% KCl solution [51], Reprinted with permission from URTEC.

Takahashi et al. studied the temperature dependence of the degradation rate of PGA in freshwater conditions (0.05% KCl solution) (Figure 14) [51]. The degradation of PGA occurred via hydrolysis, and the reaction rate of hydrolysis simply increased with the temperature. Thus, the degradation rate of PGA increased exponentially with temperature and can be expressed by an Arrhenius equation [49]. This makes the degradation of PGA predictable.

**Figure 15:**
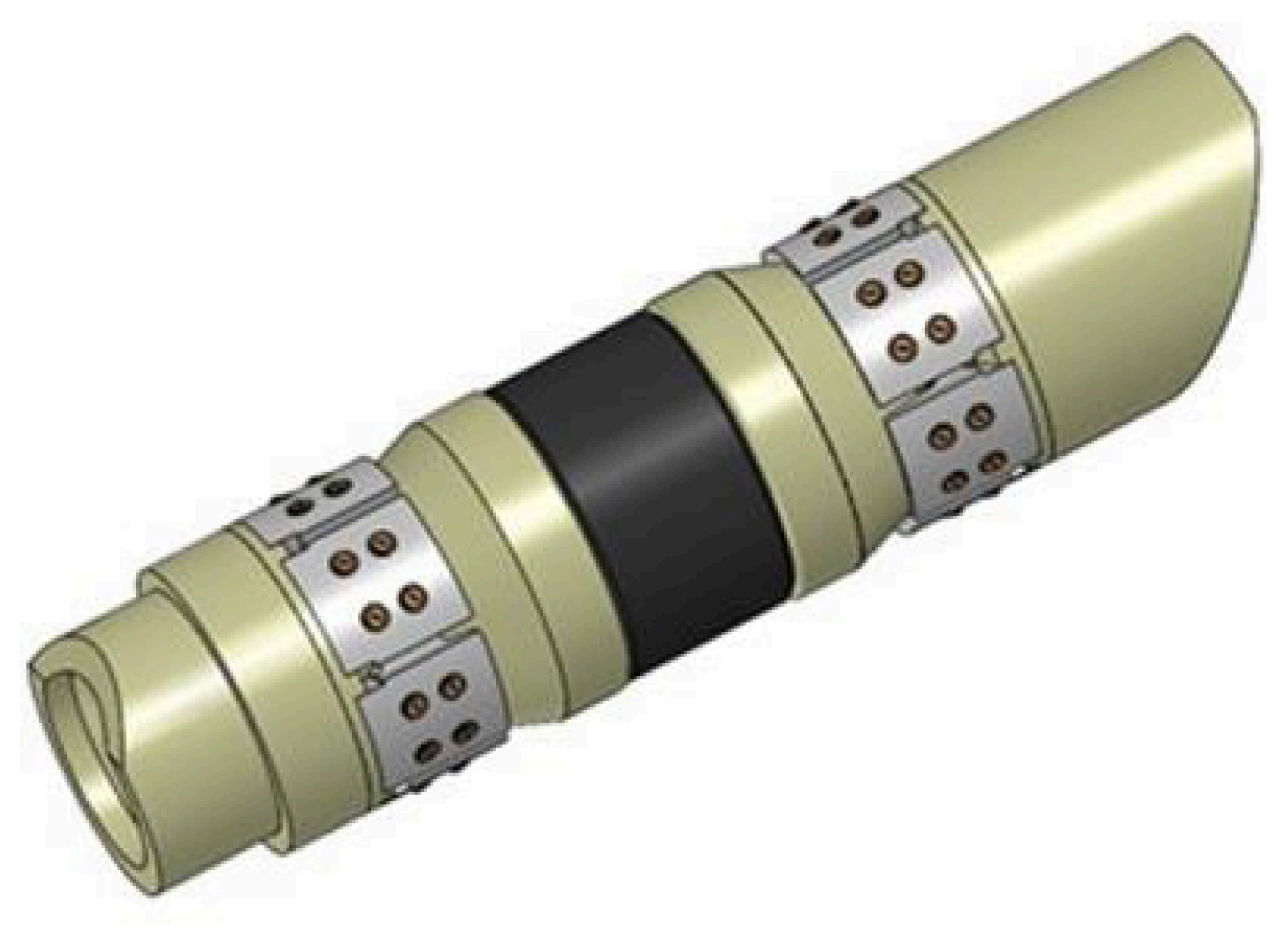
Kureha Degradable Frac Plug with PGA major parts [40,50]. The plug design includes the degradable PGA (yellow color) and dissolvable sealing elements (black color).

Kureha Energy Solution (Kureha Inc., Houston, TX, USA) [40,49,51] has successfully developed degradable plugs using PGA as the primary component (Figure 15). The Kureha Degradable Frac Plug has a hybrid design, which includes the degradable polymer (polyglycolic acid, PGA) and dissolvable sealing element that predictably degrade to provide a clear wellbore for production without intervention and assist in reaching longer laterals that coil tubing cannot reach. PGA is a solely temperature-dependent degradable polymer, eliminating the need for chlorides, which can be highly dependent on wellbore conditions. This makes this plug more predictable. They also mentioned that the plug can be used within broad temperature ranges of 125 °F to 325 °F (52 °C to 163 °C) and up to a 10,000 psi (69 MPa) frac pressure. The Kureha Degradable Frac Plugs were successfully and extensively used in the fracturing of the oilfields in North America by replacing millable polymer composite plugs.

**Figure 16:**
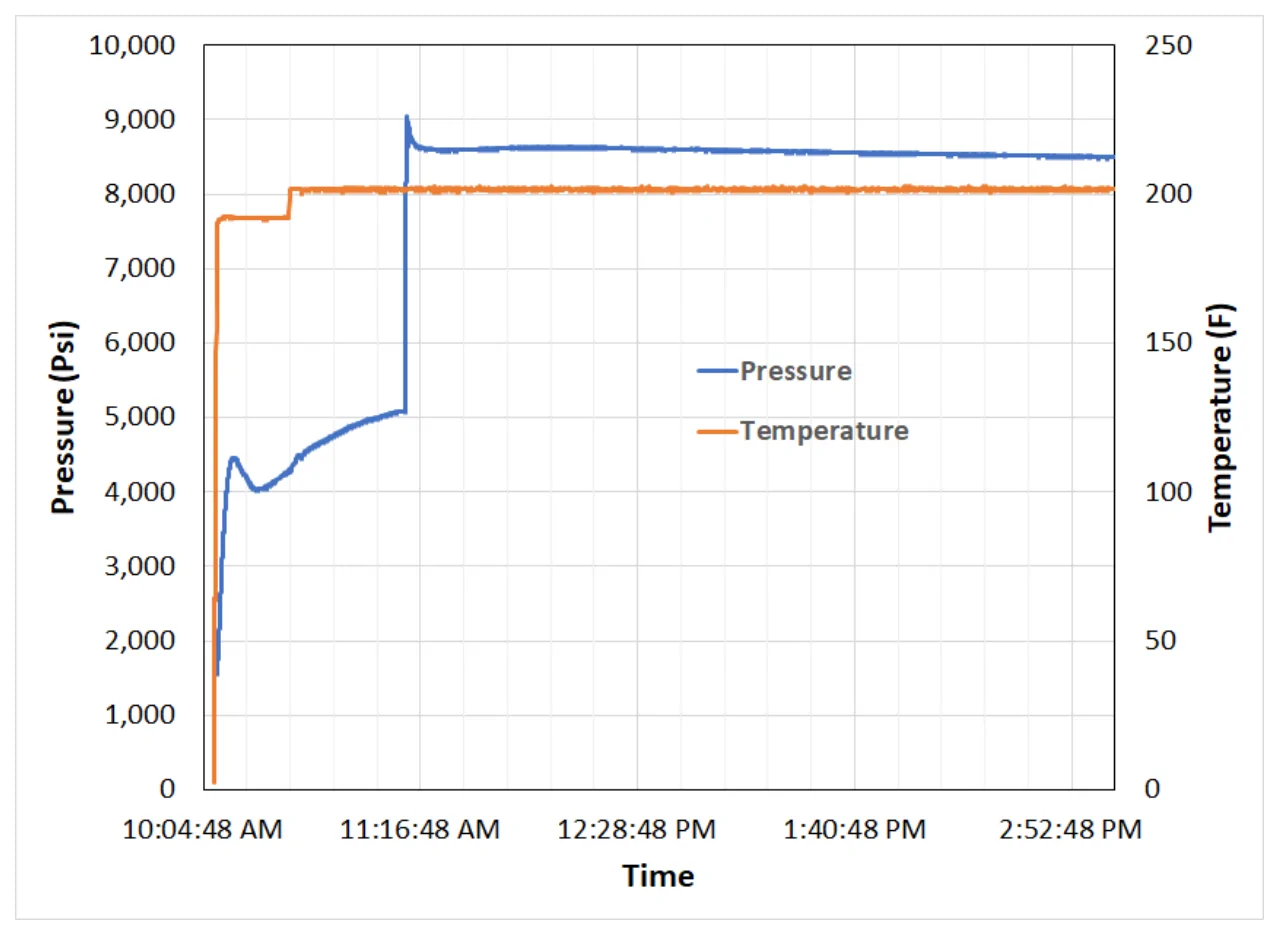
The testing profiles of the pressure holding of the degradable frac plug at 93 °C and 60 MPa [52].

**Figure 17:**
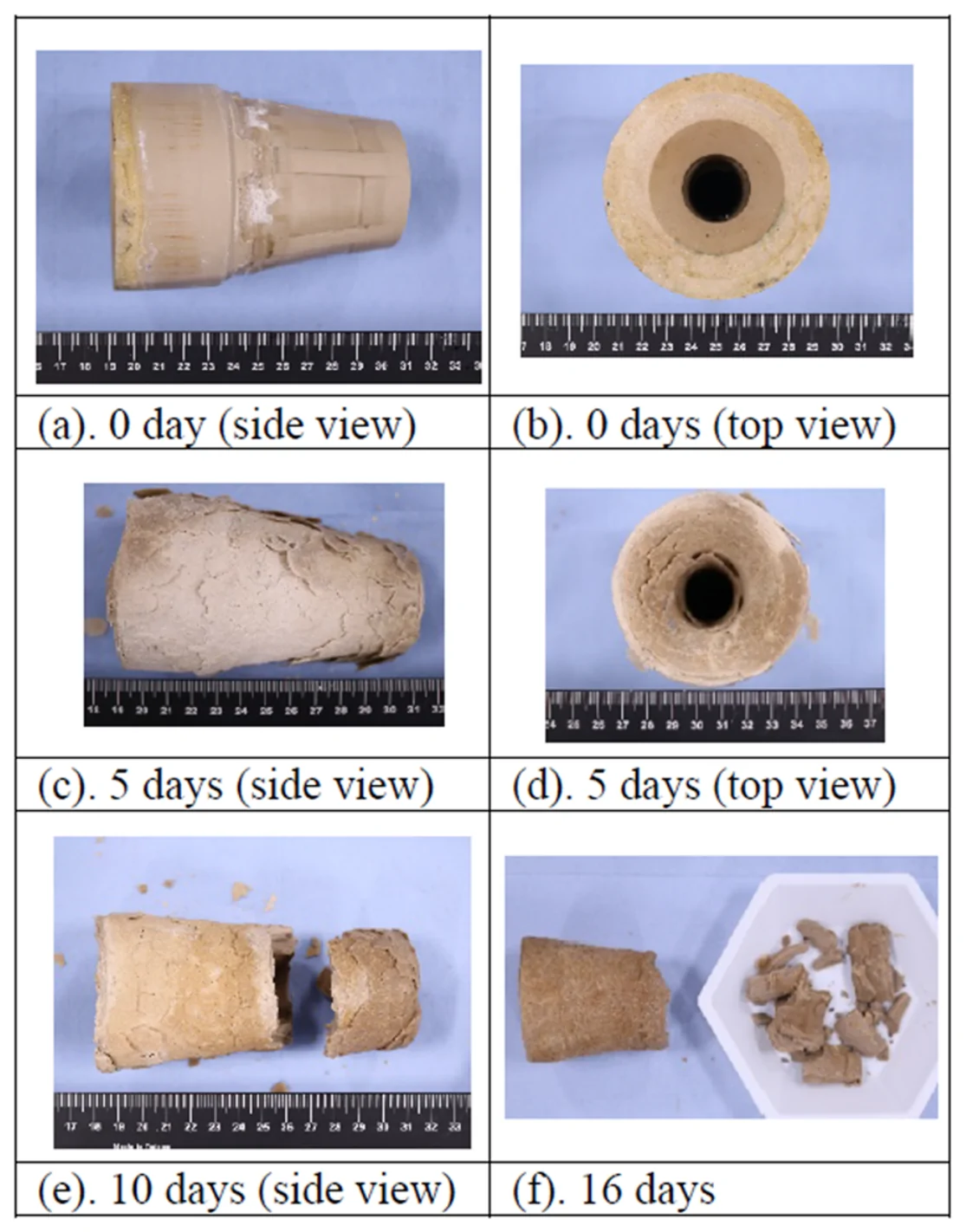
The photos of the PGA-based ramp cone at different degradation times at 80 °C with the KCl solution [52].

Xing et al. successfully developed a degradable frac plug utilizing a PGA-based material and a ramp design [52]. The hybrid degradable plug features a ramp cone made from a PGA-based degradable material, a dissolvable rubber seal, and dissolvable magnesium slips. It successfully passed a pressure-holding test at 93 °C and 60 MPa (Figure 16 [52]). Figure 17 demonstrates the degradation behavior of the PGA-based ramp cone from the hybrid plug at different intervals during exposure to an 80 °C KCl solution. Photographs of the ramp cone at various degradation times in the KCl solution at 80 °C are also provided [52].

## 4. Degradable Thermosetting Polymers

As discussed previously, the development of degradable thermoset polymers benefits from advancements in two key industries: the recyclable thermoset industry, also known as green chemistry [53], and the biodegradable materials industry. Given the niche market in the oil and gas sector, many ideas and technologies in this area are drawn from these fields. Considering the successful application of PGA thermoplastic materials, as mentioned in earlier sections, the primary market for thermosets will likely be in high-temperature applications (typically > 120 °C), owing to their highly crosslinked network structure. However, this structure often results in poor solubility. Therefore, a careful balance between temperature resistance and solubility is necessary, or innovative solutions will be required to improve both properties simultaneously. This section will provide a systematic review of the development of degradable thermoset composites for downhole conditions, focusing on those that have been successfully commercialized. Technologies with potential for commercialization in the oil and gas industry will also be discussed. While some technologies relying on complex or high-cost chemistry may be technically promising, they are better suited for other high-end industries, such as medical or military sectors, where higher costs can be justified. These technologies will not be covered in this review due to their limited potential for commercialization in the oil and gas industry.

### 4.1. Cyanate Ester

**Figure 18:**
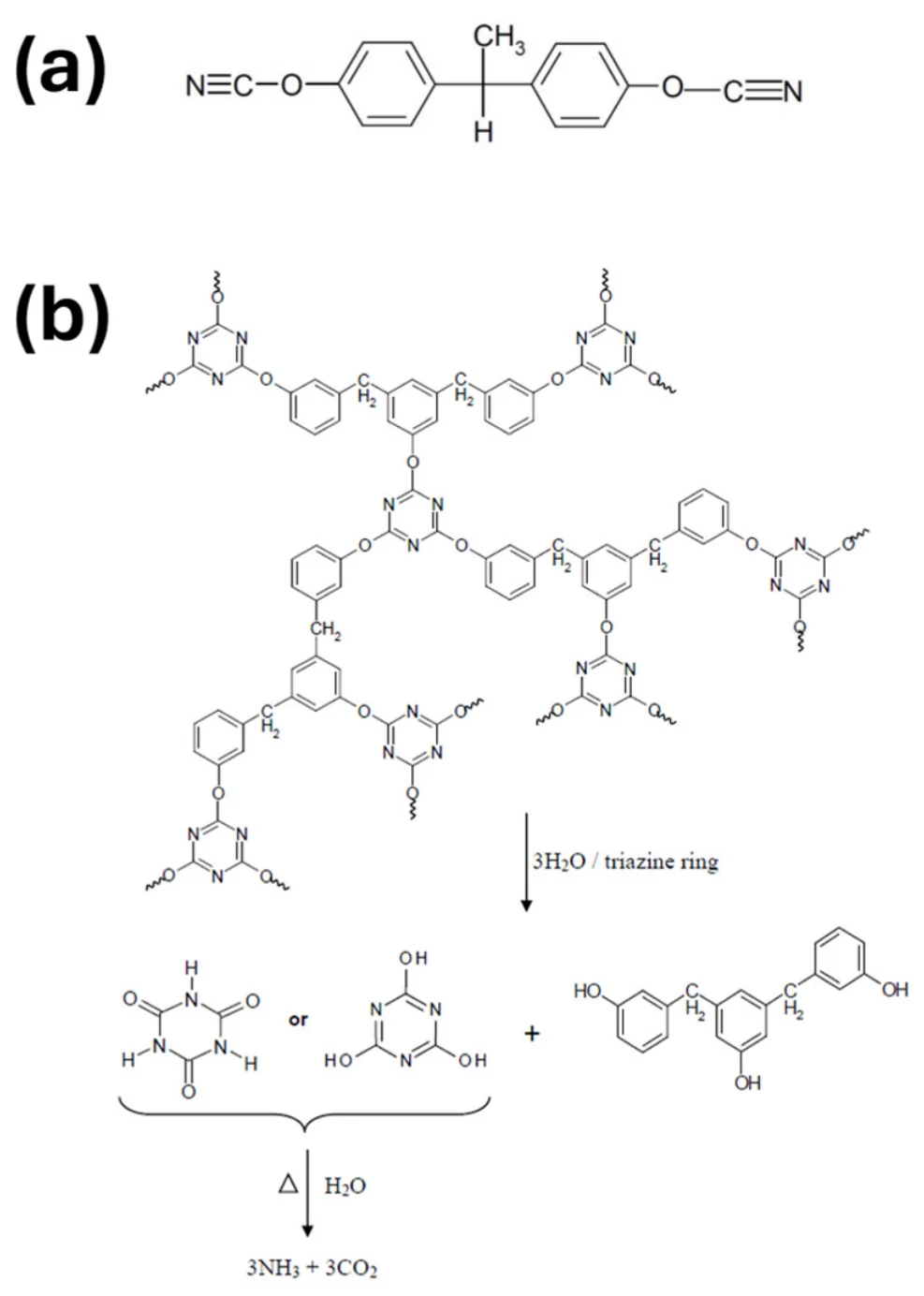
Molecular structure of bisphenol E cyanate ester monomer (**a**), and the hydrolysis reaction process (**b**) [55].

Cyanate ester resins are advanced thermosetting polymers highly regarded for their exceptional thermal and mechanical properties, with their molecular structure depicted in Figure 18a. These resins feature extremely high glass transition temperatures (Tg) and dense crosslinking networks, which contribute to their excellent dimensional stability, low thermal expansion, and superior performance in extreme environments [54]. These characteristics make cyanate ester resin a preferred material for high-temperature applications in critical industries such as aerospace, defense, and electronics, where reliability under thermal and mechanical stress is essential. They are commonly used in structural components for aircraft, spacecraft, and missiles, as well as in high-performance electronic devices, thanks to their low dielectric constants and minimal loss factors.

Despite their many advantages, cyanate ester resins have a notable drawback: sensitivity to moisture [56]. The resin is prone to hydrolytic degradation under humid conditions, which can lead to water absorption and the chemical breakdown of the polymer network (Figure 18b). This issue is especially problematic in electronic applications, such as printed circuit boards (PCBs) for integrated circuits (ICs), where prolonged exposure to moisture can result in blistering and delamination. These failures undermine the reliability of electronic components and shorten the lifespan of devices. However, this moisture sensitivity presents a unique opportunity in the niche market for dissolvable plug applications in the oil and gas industry, where high Tg and strength are required, along with the ability to dissolve in water.

This material was first introduced by scientists at Baker Hughes, a major oil services company [55]. They developed a new system to address the limitations of commercially available polymeric frac balls and other downhole tools made from polyglycolic acid (PGA), a thermoplastic with a low glass transition temperature (Tg) of approximately 104 °F and a high cost. While cyanate esters also exhibit hydrolytic properties, their reaction rate is too slow for practical use as dissolvable materials. The key innovation involves adding a catalyst to accelerate the dissolving process, enabling complete dissolution within one week. This catalyst, known as a proprietary additive developed by Baker Hughes, represents the core intellectual property (IP) of these technologies. To overcome the brittleness of cyanate esters, short fibers are incorporated into the composite, enhancing its mechanical properties without the risk of blocking the wellbore after the resin dissolves.

According to their publication [55], the degradable composite based on cyanate ester resin achieves a glass transition temperature (Tg) of 280 °C. Through systematic tuning and the optimization of the molding process, the composite reinforced with quarter-inch chopped glass fibers exhibits impressive mechanical properties, including a compressive strength of 289 MPa, a tensile strength of 109 MPa, a tensile modulus of 25 GPa, and an elongation at break of 0.48%. The composite degrades in neutral brine water at temperatures of 95 °C and above, with the degradation rate increasing as the temperature rises. The first, and possibly the only, commercial product from Baker Hughes to date is the frac ball. This product stands out for its ability to degrade without consuming excessive amounts of water or blocking the wellbore after dissolution, addressing a key limitation of dissolvable metals, which often face these challenges.

**Figure 19:**
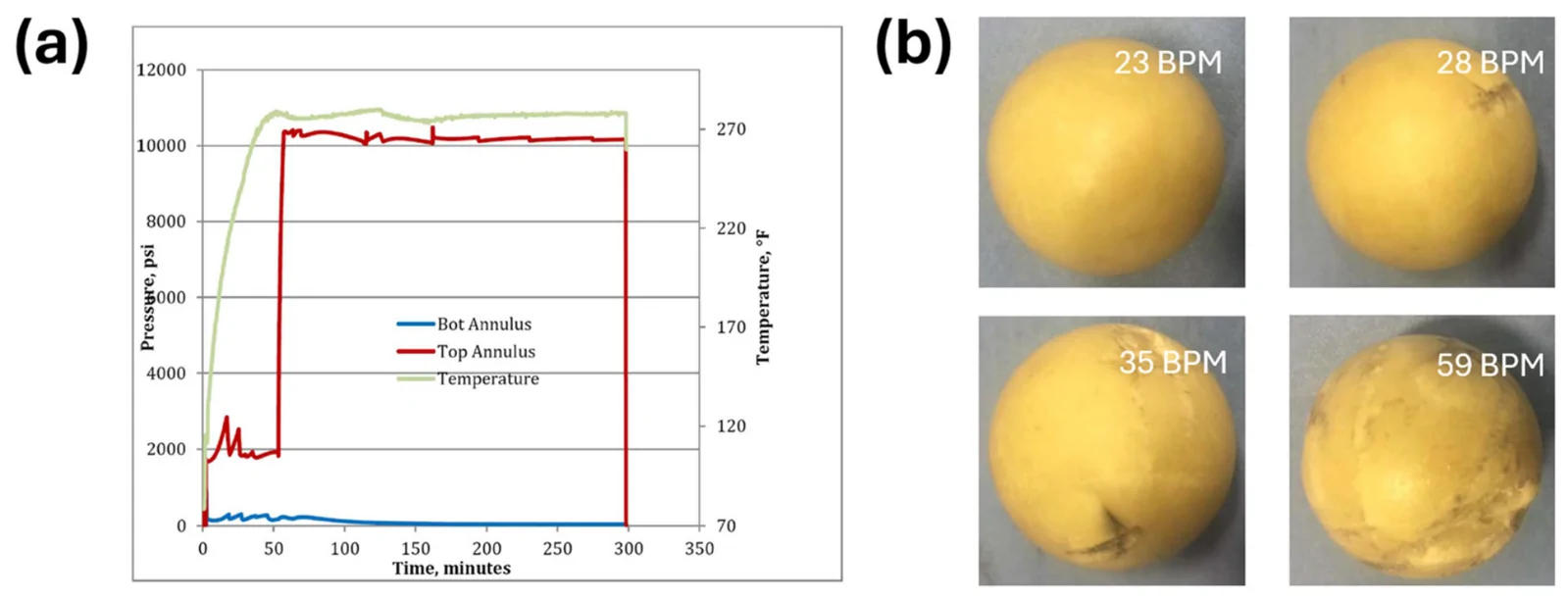
(**a**) Pressure testing of a polymer ball seated on a conical surface, maintaining a differential pressure of 10,000 psi at 200 °F for 4 h. (**b**) Post-impact images of the ball after simulation tests conducted through a pump at varying flow rates of 23, 28, 35, and 59 BPM (barrels per minute) [57].

**Figure 20:**
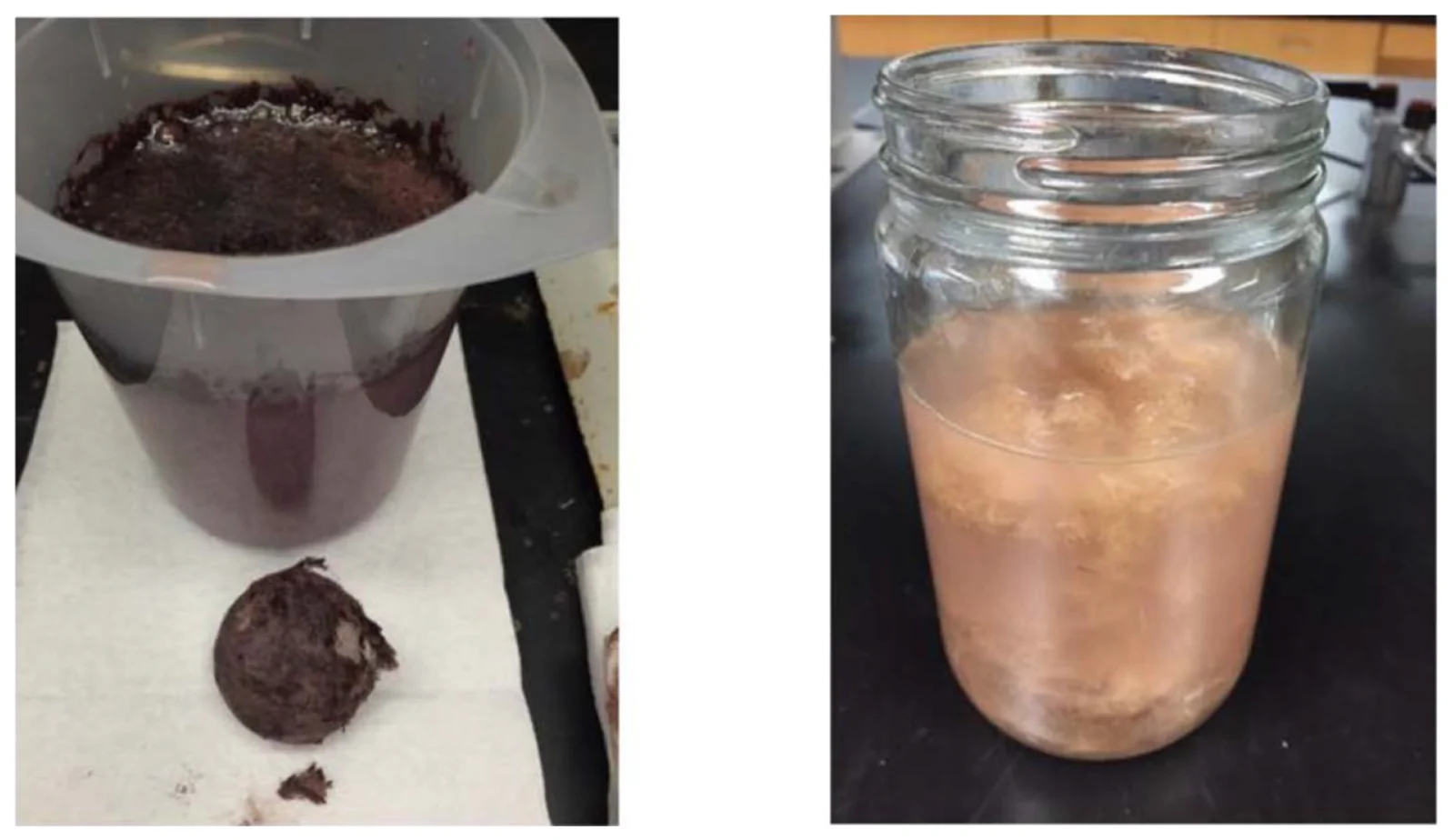
Degraded ball removed from the reactor after degradation (**left**) and after hand shaking in a container (**right**) [55].

The pressure testing of the high-Tg degradable polymeric balls demonstrated their ability to successfully withstand a differential pressure of 69 MPa for four hours at both 95 °C and 135 °C (Figure 19a). Impact test results showed that the balls could endure trips to the ball seat at velocities of 30 bbl/min (Figure 19b), fully meeting field performance requirements [57]. At 95 °C, the resin component of the ball fully dissolves within 14 days, while at temperatures above 120 °C, dissolution takes less than 7 days. The dissolution rate increases with the temperature. After dissolving, the polymer resin degrades into a brown floating residue in water (Figure 20 Left). This residue also coats the outer surface of the remaining ball, which then appears as a porous, hair-like structure. When placed in a container of water and gently shaken, the residual ball unfurls into individual chopped glass fibers (Figure 20 Right).

The high Tg and exceptional compressive strength of this degradable polymer make it well suited for multi-stage hydraulic fracturing and plug-and-perf operations. The primary limitation of this technology lies in the relatively high cost of cyanate ester resin compared to other commodity thermoset resins, such as polyester and epoxy. However, its cost remains justifiable for downhole applications, given its unique performance advantages.

### 4.2. Epoxy

Epoxy is one of the most versatile thermosetting systems, thanks to the high reactivity of the epoxide group with various functional groups, including amines, acids (and acid anhydrides), phenols, alcohols, and thiols. It offers relatively good mechanical properties, excellent thermal stability, and strong bonding with glass and carbon fibers, particularly when compatible sizing agents are used. Furthermore, epoxy is likely the most cost-effective option among high-performance thermoset materials suitable for high-temperature applications. These outstanding attributes make epoxy a preferred choice for high-performance structural applications.

From a theoretical perspective, degradable epoxy resins can be formulated by incorporating crosslinkers and extenders sensitive to heat and/or aqueous conditions. When these crosslinkers and extenders degrade, the epoxy material reverts to its corresponding prepolymers. For water-degradable epoxy, it is advantageous to use a water-soluble prepolymer system, ensuring that the material dissolves completely in water after crosslink degradation. Effectively degradable crosslinkers may include thermally labile bonds, such as esters, urethanes, carbonates, and acetals, as well as specialized dynamic linkages like disulfide (e.g., dithioaniline) [58], Diels–Alder reactions (diene + alkene) [59], or advanced click chemistry approaches currently at the forefront of polymer science. To further enhance degradation, epoxy prepolymers that are inherently biodegradable, such as glycerol diglycidyl ether, can be employed.

**Figure 21:**
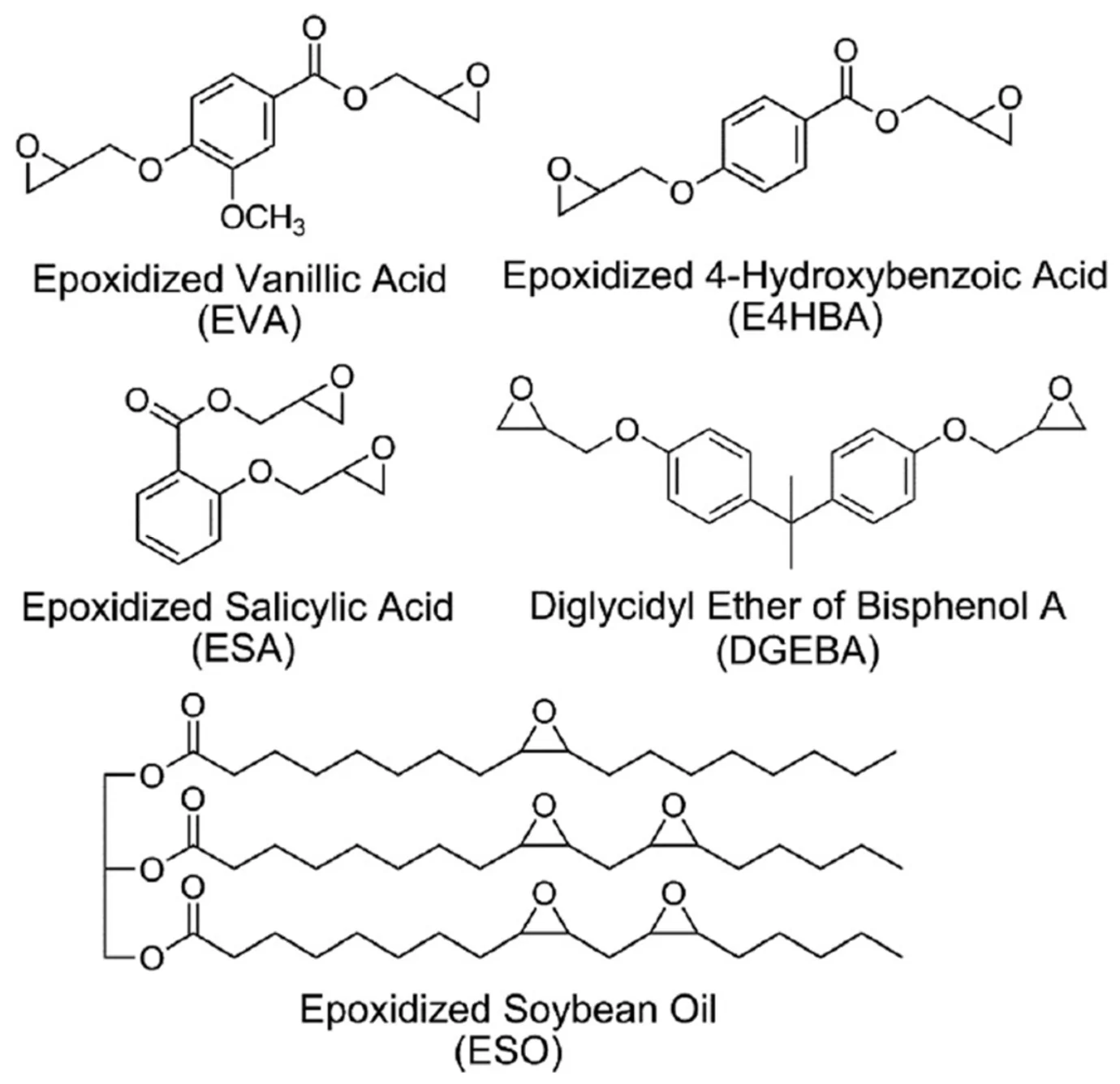
Chemical structures of epoxidized vanillic acid (EVA), epoxidized salicylic acid (ESA), epoxidized 4-hydroxybenzoic acid (E4HBA), epoxidized salicylic acid (ESA), epoxidized 4-hydroxybenzoic acid (E4HBA), epoxidized soybean oil (ESO), and diglycidyl ether of bisphenol A (DGEBA) [15].

The literature offers numerous examples of biodegradable epoxy thermosets. For instance, Shen et al. developed biodegradable epoxy resins containing ester linkages by replacing conventional bisphenol A epoxy monomers with epoxidized soybean oil (ESO) [15]. The molecular structures of ESO and conventional petrochemical-derived epoxy are shown in Figure 21. ESO’s high degradability is attributed to its long, flexible aliphatic polymer chains, which minimize steric hindrance, allowing water molecules to diffuse more freely and facilitating hydrolysis. However, this flexibility also limits the resin’s temperature resistance due to its low crosslinking density and lack of aromatic groups, as seen in bisphenol A epoxy (Figure 21). In addition to these academic examples, commercially available water-soluble epoxy resins exist. One well-known system is the Durcupan resin, composed entirely of water-soluble components. Produced by Fluka (Buchs, Switzerland), a subsidiary of Sigma-Aldrich (St. Louis, MO, USA), this resin is used for embedding electron microscope samples in plastic. For the water-soluble version, aliphatic polyepoxide replaces the non-water-soluble Modified Araldite M resin (a bisphenol-A-based resin). Despite these innovations, such technologies are unsuitable for the oil and gas industry due to their high cost and inadequate temperature ratings (<100 °C), as discussed in Section 2.

**Figure 22:**
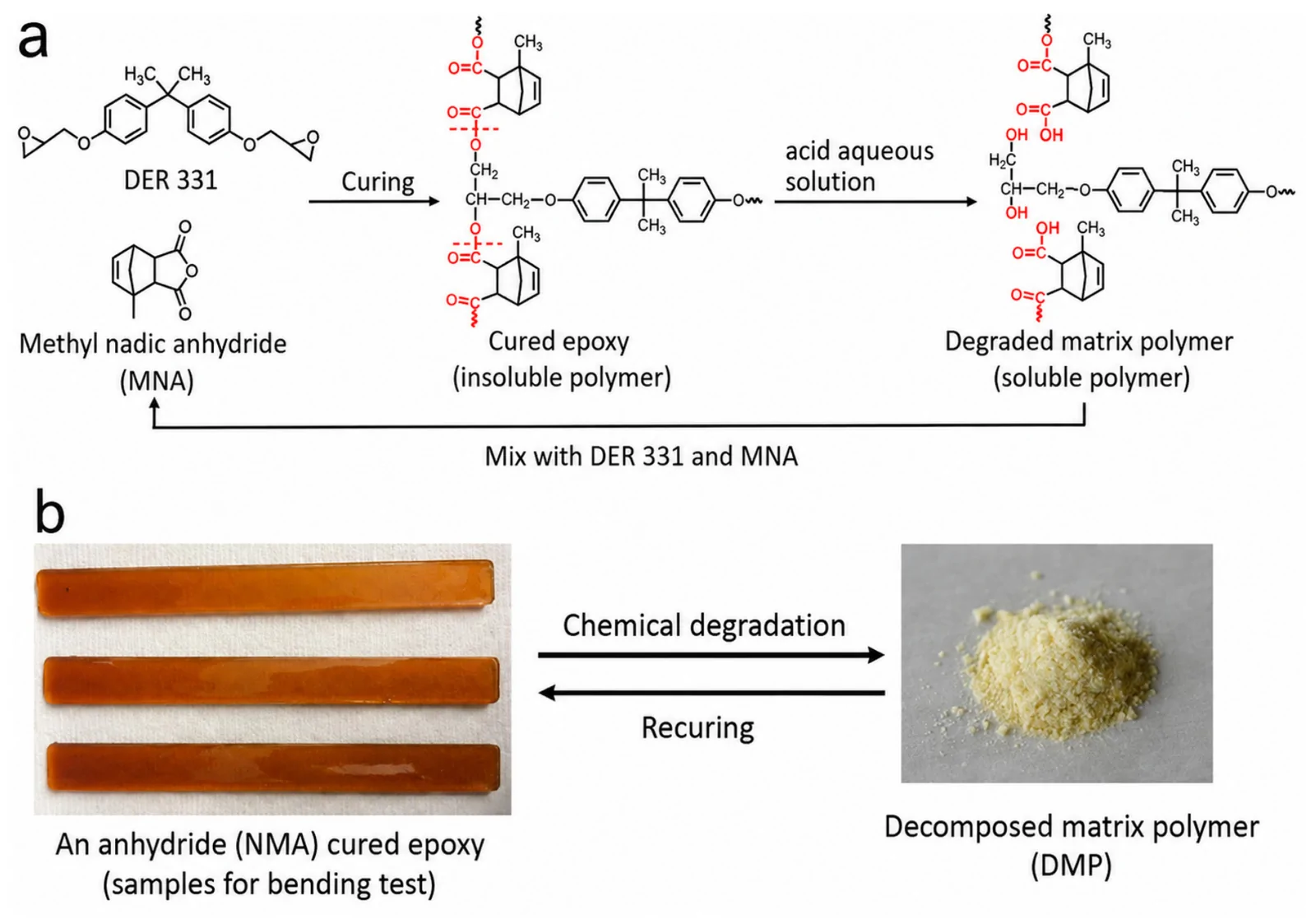
(**a**) Chemical structure of DER 331 epoxy resin and nadic methyl anhydride (NMA) and their cured system; (**b**) digital photos of an anhydride (NMA)-cured epoxy and decomposed matrix polymer (DMP) [16].

Structural engineering epoxy resins and composites are predominantly derived from the petrochemical industry, with a significant portion based on bisphenol epoxies. Higher-end industries, such as aerospace and chemical processing, often use multifunctional epoxy systems for superior performance. The growing adoption of these materials in various industries, replacing traditional metals—such as in wind turbines made from fiber-reinforced epoxy composites—has raised concerns about their environmental impact. The increasing waste from such materials poses a significant challenge for environmental protection. In response, the green industry has invested heavily in fundamental research and engineering solutions to develop degradable alternatives and recycling methods. These efforts aim to reduce landfill waste while recovering valuable fiber reinforcements, such as carbon fiber, to lower overall costs. A comprehensive review on this topic is available [13]. Among the many studies in this field, Zhang et al. provide an excellent example of chemical degradation for anhydride-cured epoxy resins. They developed an efficient method using an environmentally friendly phosphotungstic acid (HPW) aqueous solution as a catalyst under mild reaction conditions at 190 °C (Figure 22) [16]. During the reaction, the ester bonds in the crosslinked structure were selectively cleaved, fully converting the thermosetting polymer into oligomers containing multifunctional reactive groups.

Significant progress has been made in the green chemistry industry, yet high-temperature degradable epoxy systems often require extreme conditions, such as high temperature, high pressure, strong acids or bases, toxic solvents, or supercritical fluids, to achieve degradation [13]. These challenges stem not only from the inherent strength of epoxy bonds but also from two primary factors: strong steric effects and low solvent diffusion rates. The strong steric effects arise from functional groups surrounding the degradable bonds within the polymer network. To enhance the glass transition temperature and thermal stability, various functional groups, such as benzene rings, are introduced into the epoxy and hardener molecules. While these groups increase thermal performance, they also limit the mobility of linkage bonds and neighboring molecular chains, making it difficult for degradable ester bonds to undergo hydrolysis, as water molecules struggle to access them. Low solvent diffusion rates are another significant barrier, caused by the highly condensed and rigid polymer network. In thermosetting materials, molecular chain rotation and relative movement occur only above the glass transition temperature. At such elevated temperatures, spaces between molecular chains form channels that facilitate water molecule diffusion. Below the glass transition temperature, however, the molecular chains “freeze,” drastically reducing water uptake and diffusion, thereby impeding the degradation process [7].

Highly corrosive environments are essential for degradation testing and are typically provided in facilities using autoclaves or degradation tanks. However, such extreme conditions are not present in actual downhole environments. Developing a degradable polymer composite system with a high-temperature rating that can degrade readily in mild aqueous conditions at low temperatures presents a significant challenge due to the conflicting nature of these requirements based on currently available techniques. To address this issue and enable large-scale industrial applications, Zhao et al. selected industrial-grade epoxy resins and other readily available raw materials [7]. They devised a novel chemistry and microstructure to create a high-temperature (>130 °C) degradable epoxy polymer composite that efficiently degrades in near-neutral aqueous environments at lower temperatures (≤95 °C).

**Figure 23:**
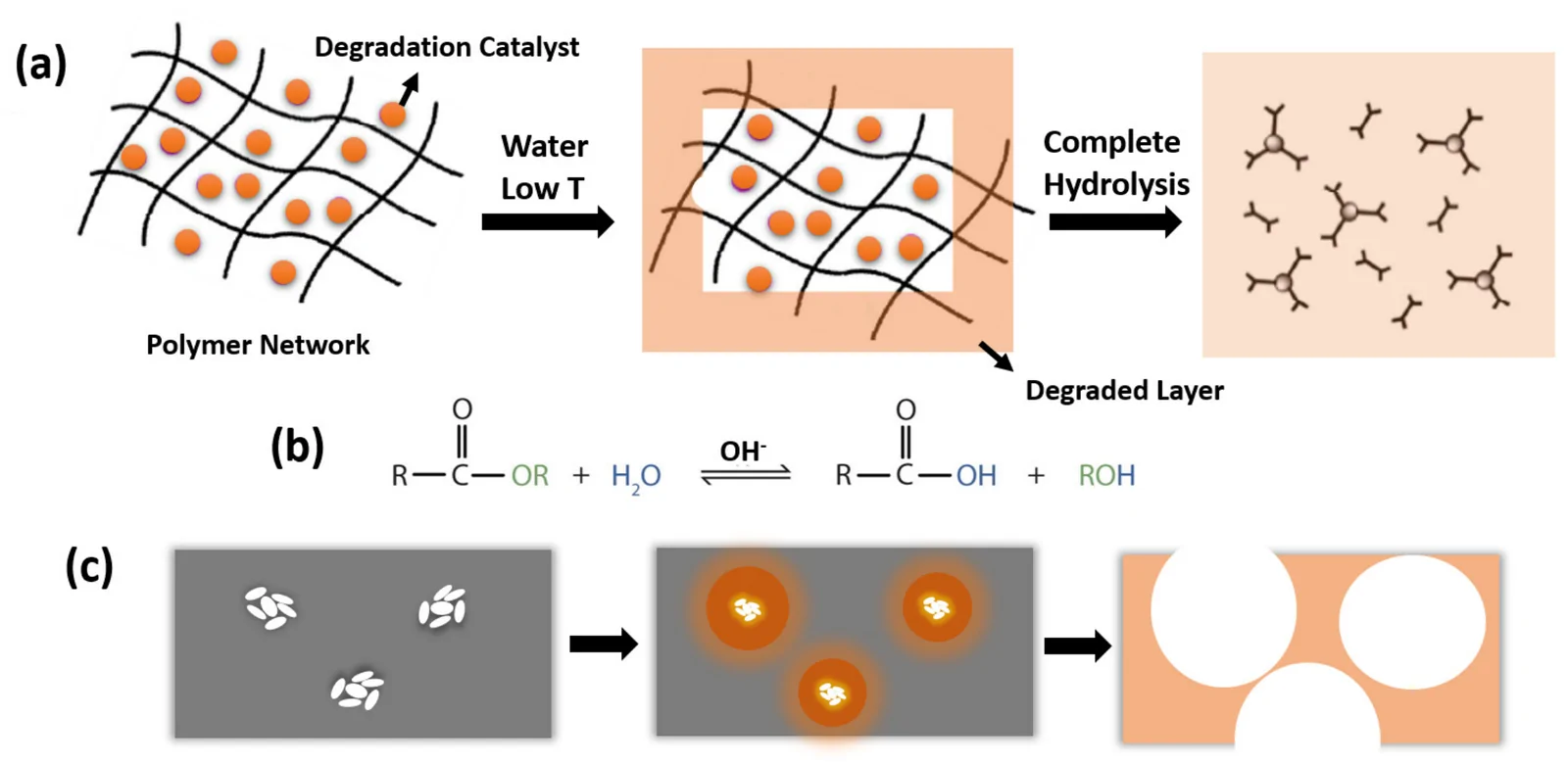
Schematic illustration of degradable epoxy hydrolysis process (**a**), base-catalyzed hydrolysis reaction (**b**), and explanation of the foam microstructure formation mechanism (**c**) [7].

In this study, ester bonds were selected due to the well-established epoxy/anhydride systems used in industrial manufacturing and their successful application in high-temperature epoxy composites, particularly within the oil and gas industries. A degradation catalyst was introduced into the polymer matrix system, with optimal effectiveness achieved by positioning it near the ester linkage bonds (Figure 23a, left). This arrangement creates a highly catalytic local environment around the bonds, significantly accelerating the hydrolysis process (Figure 23a). Since solid acids tend to react with unreacted epoxy groups at elevated temperatures, the fine powders of the solid bases—such as Ca(OH)_2_, CaO, NaOH, and Mg(OH)_2_—were identified as suitable, cost-effective degradation catalysts. The degradation mechanism follows a base-catalyzed hydrolysis reaction, as illustrated in Figure 23b. To further explain the process, this study proposes a mechanism shown in Figure 23c. The incompatibility between the organic epoxy resin and the ionic inorganic catalyst powders results in the formation of small segregates, uniformly dispersed within the resin matrix (Figure 23c, left). As water permeates the system, these segregates dissolve gradually, creating localized, highly alkaline microenvironments that accelerate the hydrolysis of epoxy ester linkages, leading to degradation. Simultaneously, the hydrolysis products dissolve and form hollow bubbles that expand during the degradation process (Figure 23c, middle). As these bubbles merge, they form continuous pathways to the surrounding aqueous environment, resulting in the complete dissolution and significant dilution or removal of the catalyst by the water. This loss of catalyst slows the degradation process, leaving behind an undegraded skeleton and producing a porous foam structure (Figure 23c, right). These findings highlight the importance of achieving uniform and finely dispersed catalyst powder throughout the polymer matrix to ensure effective and consistent degradation.

**Figure 24:**
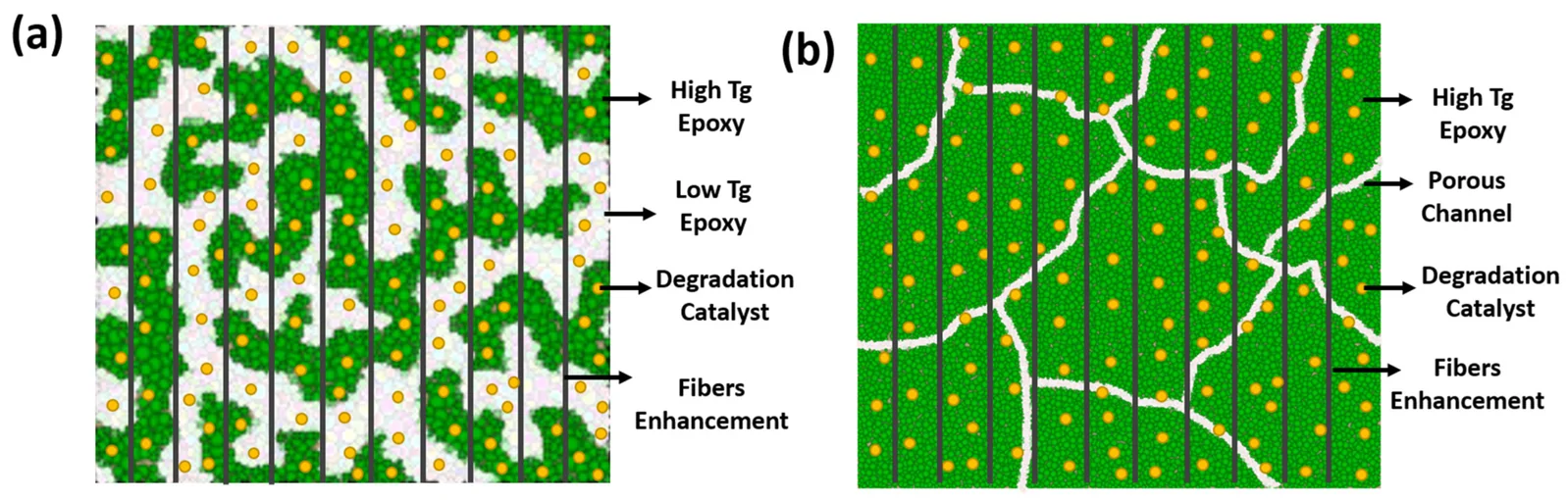
Schematic illustration of degradable epoxy composite with a bi-continuous microstructure (**a**) and with an open-pore microstructure (**b**).

Having successfully achieved effective ester hydrolysis in the studied high-temperature system through the introduction of a degradation catalyst, the primary bottleneck restricting the degradation rate lies in the diffusion rate of water molecules into the highly crosslinked and dense epoxy matrix. Therefore, new strategies are required to provide an effective water pathway or “highway” for the material. In response to this challenge, two microstructures have been evaluated: the bi-continuous microstructure and the open-pore microstructure. The bi-continuous microstructure (Figure 24a) is made by blending compatible high-Tg epoxy with lower-Tg epoxy in a carefully chosen ratio. In this design, the high-Tg epoxy forms the backbone of the bulk material, serving as the continuous and dominant phase that provides excellent mechanical strength at elevated temperatures. Meanwhile, the low-Tg epoxy acts as the facilitating phase, contributing to an accelerated degradation rate at lower temperatures. As the degradation progresses and the low-Tg epoxy dissolves, it creates increased open channels or pores within the material. These open channels provide a “highway” for water molecules to diffuse, enabling efficient penetration throughout the material and accelerating the degradation process of the bulk material. This strategy is found not very effective, because it is challenging to maintain a high T_g_ in the overall system with an increased loading of the low-Tg component to form effective and connected open channels or pores. In the open-pore microstructure design (Figure 24b), a rigid and highly condensed high-Tg epoxy network is deliberately maintained to preserve its high-temperature rating, while open pores or channels are intentionally introduced to facilitate high water diffusion. Similarly to the previous strategy, short glass fibers are also incorporated to enhance mechanical strength, and a degradation catalyst is introduced into the polymer matrix to enable its degradation property. This strategy has been proven successful, and the key technology is creating these micropores or channels during the material fabrication process; thus, the IP of this technology is the initial formation and processing.

**Figure 25:**
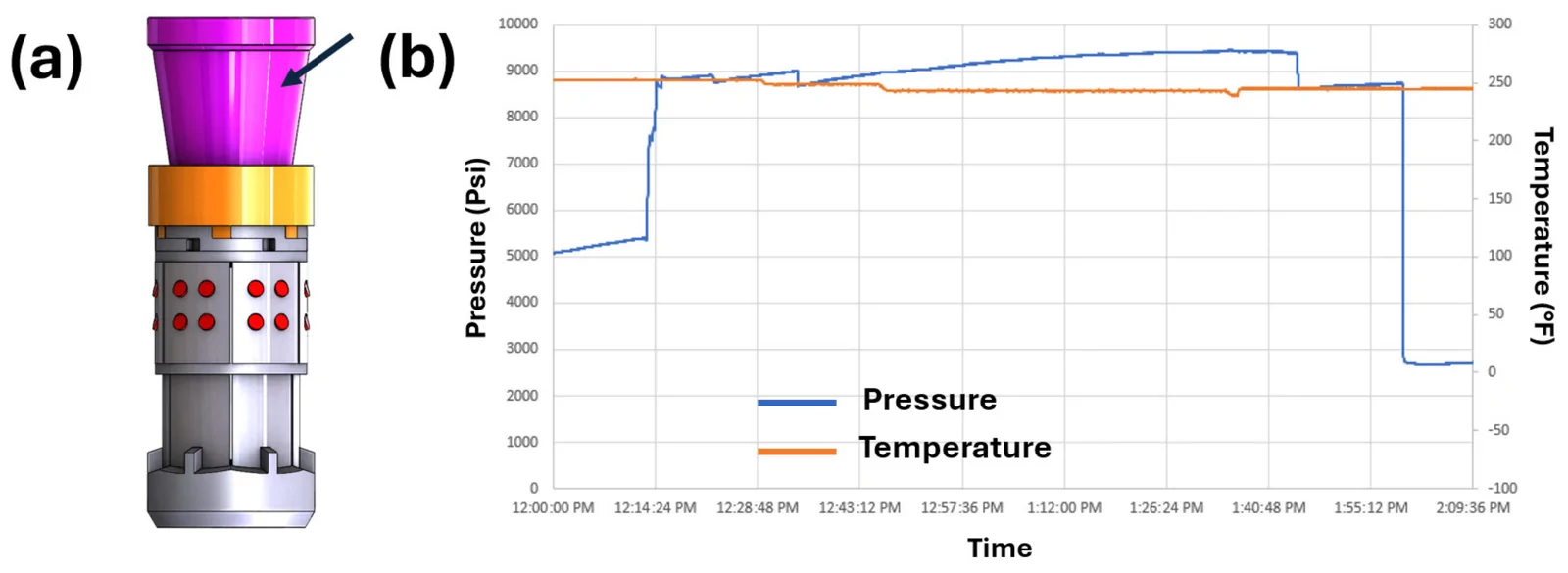
Image of hybrid acid frac plug with the degradable composite component indicated by the arrow (**a**), and its pressure test curve at 120 °C (248 °F) (**b**) [6].

This technology enables the production of a final composite with a temperature rating exceeding 150 °C, owing to its highly crosslinked molecular structure. The material exhibits extremely high tensile strength, ranging from 138 to 207 MPa. It is used in the cone design of dissolvable plugs for acid-fracturing applications, as indicated by the black arrow in Figure 25a. The composite can withstand pressures above 62 MPa at 120 °C and has the potential to perform at temperatures exceeding 150 °C, thanks to the high glass transition temperature (Tg) of the thermoset polymer composite (Figure 25b). This hybrid plug fully dissolves or degrades within two weeks, leaving only fine glass fiber residue, which does not interfere with production.

### 4.3. Polyester

Polyester is a broad category that includes both thermoplastic and thermoset materials. Thermoplastic degradable polyesters have seen successful applications in various industries and have been commercialized in the oil and gas sector, as discussed in the previous section. This section, however, focuses on thermoset composites. Thermoset polyester makes up a significant portion of current composite material fabrication due to its low material cost and fast curing process. It is generally considered to be of lower grade than epoxy composites, primarily due to its lower glass transition temperature (Tg) and chemical resistance, which result from a lower crosslink density and a higher number of ester groups that are more prone to hydrolysis. So far, thermoset polyester has been mainly used in lower-temperature applications or less corrosive environments, such as water tanks and marine boats. However, these characteristics could be advantageous when thermoset polyester is used as a degradable material, as its low cost and ease of degradability make it an attractive alternative to epoxy.

**Figure 26:**
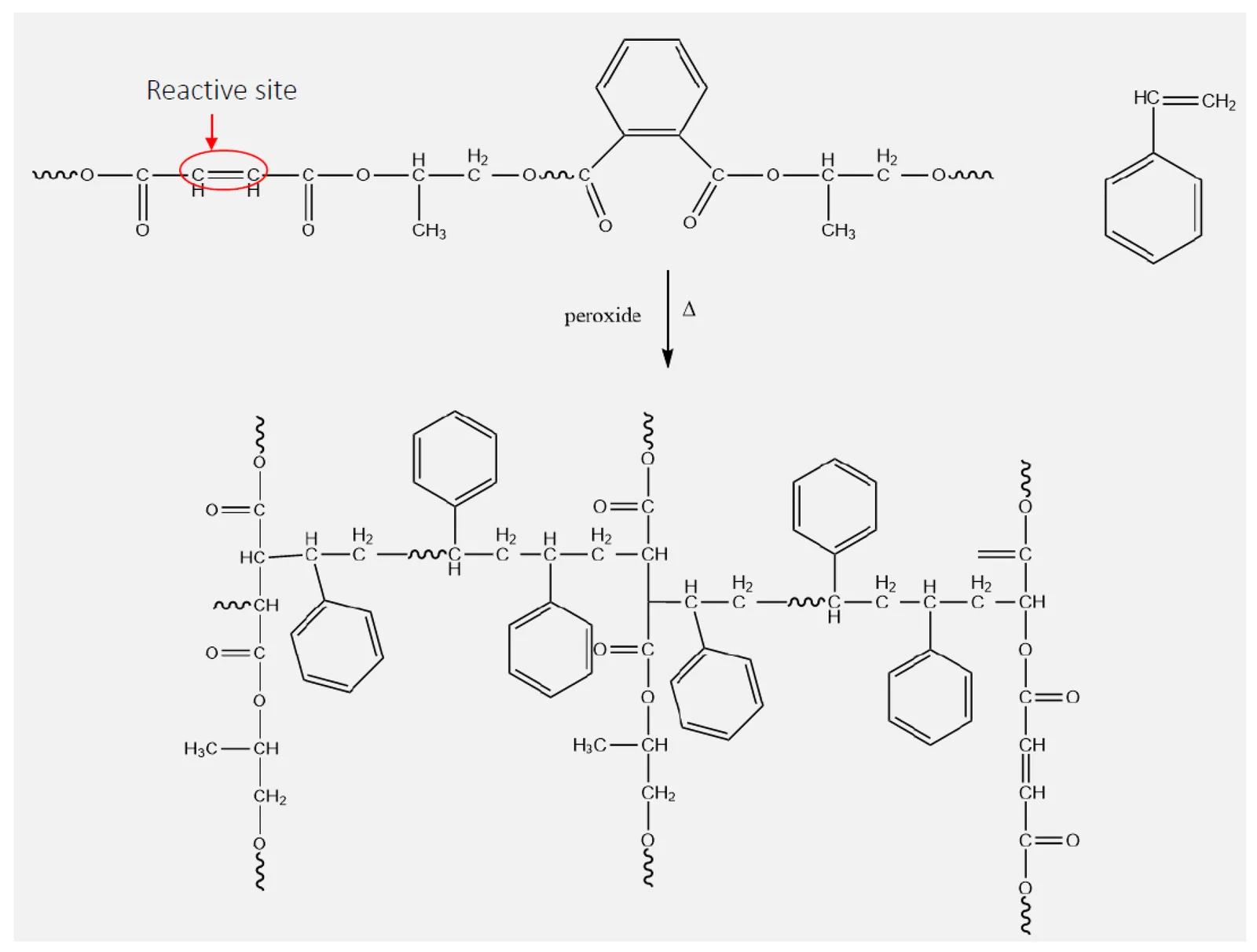
Crosslinking chemistry of unsaturated polyester.

For polyester thermosets, unsaturated polymers such as vinyl ester resins are the workhorses of the composite industry. A typical reaction process is shown in Figure 26. Compared to epoxy, vinyl ester resins have a high density of ester groups in the polymer chains and relatively large gaps between molecules due to the styrene linkage. This structure allows water molecules to diffuse into the material, facilitating degradation. These characteristics make vinyl ester resins ideal for biodegradable material applications. However, the low-crosslinking design results in a relatively low glass transition temperature (typically < 120 °C), which limits their use in high-temperature applications, such as those in HTHP (high-temperature, high-pressure) wells. For lower-temperature wells, vinyl ester resins do not offer significant cost or performance advantages over polyester thermoplastic materials. This likely explains why there are no commercially available products based on vinyl ester resins for oil and gas downhole applications.

Continuous research on this material still holds potential. For thermoplastic polyesters, it is relatively challenging to form continuous fiber-reinforced composites due to processing difficulties, such as the reaction between polyester and moisture at elevated temperatures. Currently, most products use very short chopped fibers (1–2 mm) to enhance mechanical strength, as they are compatible with traditional extrusion processes. However, due to its ease of processing, unsaturated polyester is an ideal system for producing continuous fiber-reinforced composites or composites molded with long chopped fiber (>50 mm), which offer significantly enhanced mechanical properties. There are two potential market opportunities for this material: (1) it can be used in low-temperature applications with much higher fiber loading requirements, and (2) its molecular structure can be engineered to increase the glass transition temperature (Tg) above 150 °C, while maintaining its high degradability.

**Figure 27:**
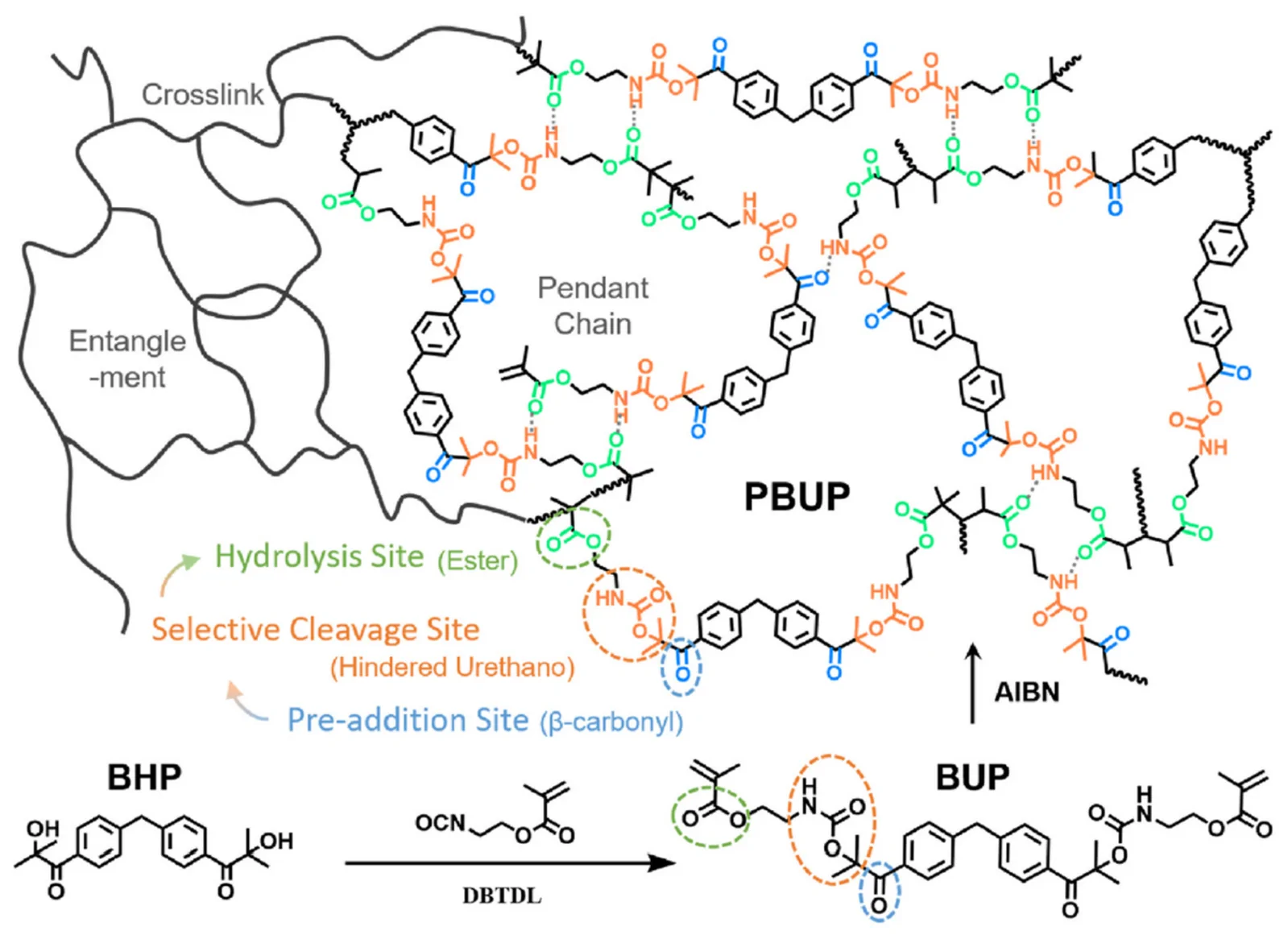
Synthetic route of degradable unsaturated polyester resins as well as their degradation mechanism [60].

Several studies in the academic literature have reported promising results for using unsaturated polyester in the oil and gas industry. For example, Huang et al. designed a degradable unsaturated polyester resin using a self-polymerizable dimethacrylate monomer containing a cleavable unit, β-carbonyl hindered urethane (Figure 27) [60]. This new resin system demonstrated a flexible strength of 79 MPa and a glass transition temperature (Tg) of 119.7 °C, making it very promising for mid-temperature well applications. Moreover, through the hydrolysis of the ester group, which was previously activated by the selective cleavage of the hindered C–O bond in the urethane, the resin could be degraded directly in an aqueous solution (a 4/1 M ratio of hydrazine and NaOH, with a total concentration of 0.04 mol/mL) at 80 °C within 4 h, a relatively mild condition. The degraded products could be easily separated by solubility. However, this technology has not yet been commercialized, and there is limited information available regarding its economic viability. Additionally, whether the degradation conditions can be replicated in downhole environments remains uncertain. Similarly to epoxy systems, a degradation catalyst might also be required.

### 4.4. Acetal Linkages

**Figure 28:**
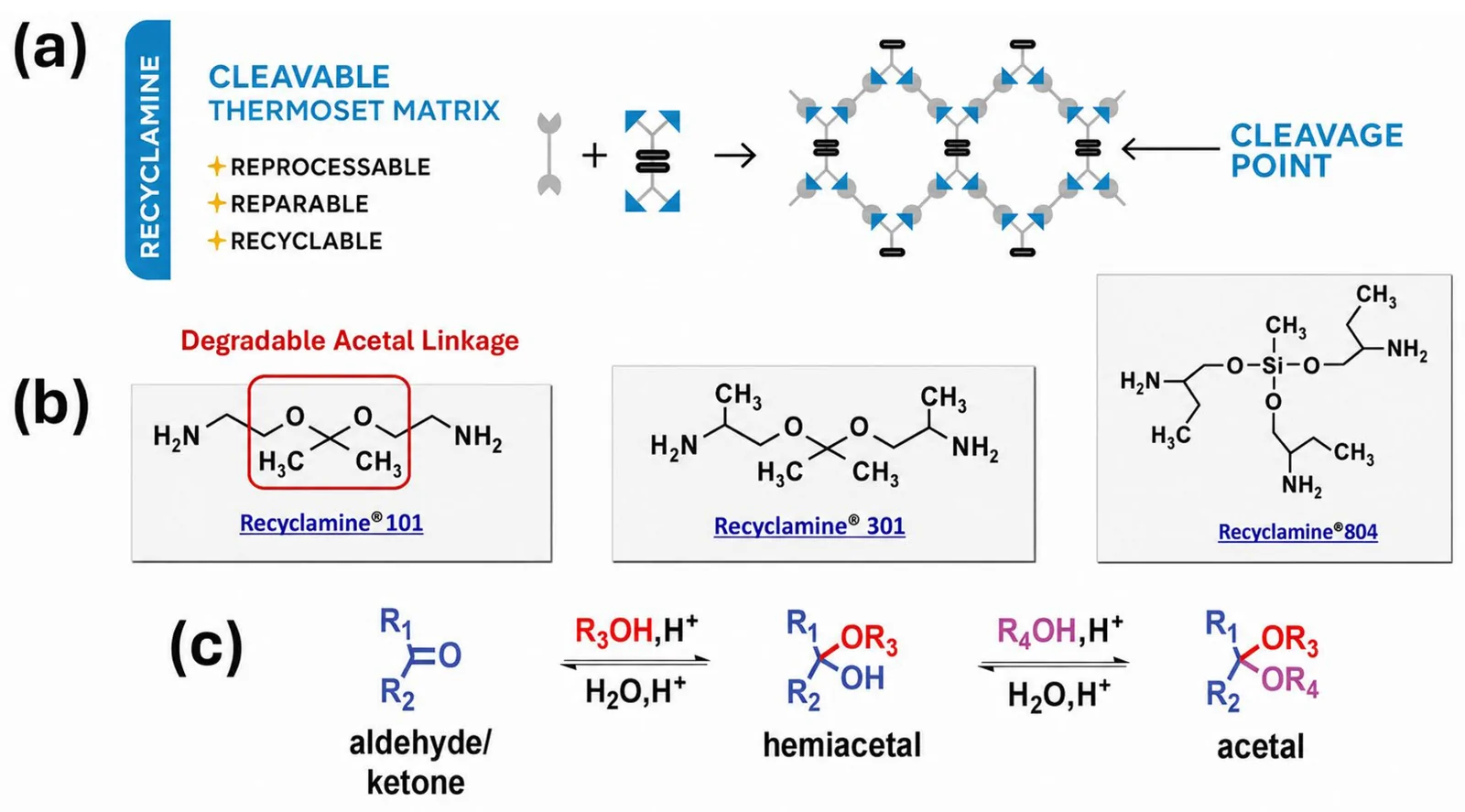
Schematic of chemical degradation method that incorporates a chemical cleavage linkage (**a**) and chemical structures of various Recyclamine^®^ hardeners grades (**b**), and the reversible synthetic route of acetal (**c**) [63].

The acetal structure is commonly used to protect the carbonyl group in organic synthesis due to its reversible nature under acidic conditions (as shown in Figure 28c) and its sufficient stability in the absence of acids [53]. Leveraging this feature, acetal linkages have been incorporated into thermosetting resins to achieve degradability. In 1996, researchers at IBM introduced the concept of diepoxies containing an acetal linkage to enable the degradation of epoxy thermosets [61]. Following this, various academic research groups and companies developed their own molecular structures or methods for producing degradable thermoset resins and composites. Epoxy thermosets based on these curing agents, as displayed on these companies’ websites, are highly durable, with tensile moduli ranging from 3.3 to 8.1 GPa and tensile strengths between 61.1 and 70.3 MPa, making them comparable to commercial epoxy thermosets.

Another common chemical approach is to selectively degrade specific linkages introduced during the curing process of the network. This method will be the focus of this section. Recyclamine^®^ hardeners were first developed and patented by Connora Technologies (Hayward, CA, USA) [62] and are now commercially available through Aditya Birla. The concept behind these hardeners is to enable the cleavage of a specific linkage within the epoxy network, facilitating post-processing. Figure 28a illustrates an epoxy network cured with a Recyclamine^®^ hardener. Conventional thermoset materials are often difficult to recycle or reuse. However, the inclusion of a chemically cleavable moiety could potentially allow for the reprocessing, repairing, or recycling of crosslinked networks. Recyclamine^®^ hardeners are commercially available in various chemistries [63], with some of the commonly used options being Recyclamine^®^ 101 and Recyclamine^®^ 301, as shown in Figure 28b.

**Figure 29:**
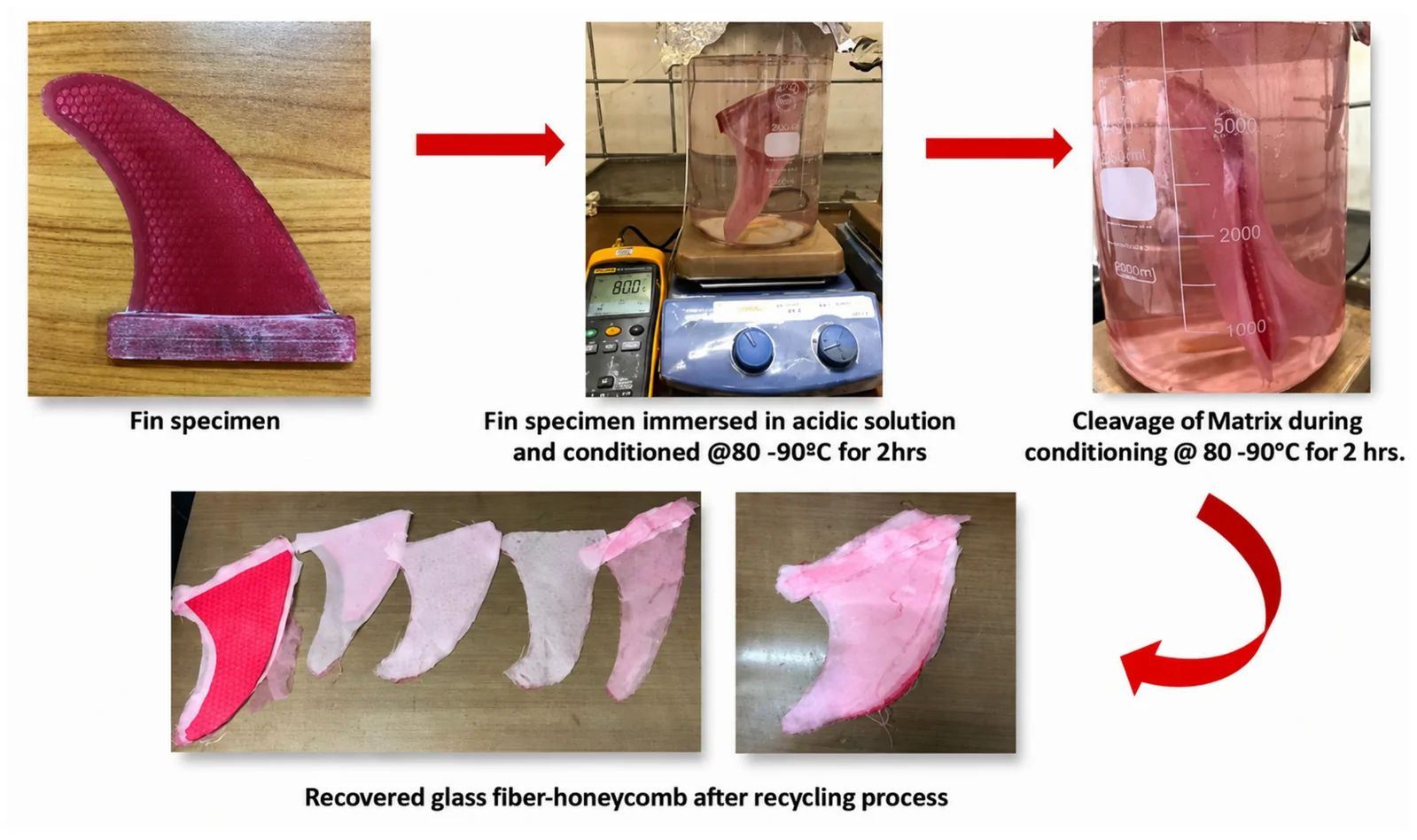
Recycling process for composites containing Recyclamine^®^ hardeners [63].

The Recyclamine^®^ hardeners are primary amines, meaning they contain the -NH_2_ group, which makes them highly reactive. Formulated epoxies using these hardeners have a good pot life at 25 °C, allowing them to be used in a variety of composite manufacturing processes, such as wet lay-up, resin infusion molding, vacuum-assisted resin transfer molding (VARTM), and other composite techniques. Recyclamine^®^ hardeners have low viscosity, and when combined with typical DGEBA epoxy resins, they achieve mid-range glass transition temperatures (60–120 °C). A recyclable RTM formulation was used to produce glass fabric-reinforced surfboard fins. In this process, a red-pigmented RTM resin formulation was injected into the surfboard-fin mold at room temperature. After curing, the composite fins were rinsed in an acetic acid solution at 80 °C for 2 h. The resin extraction process is shown in Figure 29. Within 1 h of immersion, the resin-infused composite began to soften due to the cleavage of the Recyclamine^®^ hardener linkages. After two hours of immersion, the epoxy resin matrix was completely cleaved and dissolved in the acetic acid solution.

This chemistry holds significant promise for downhole applications but has not yet been reported for dissolvable plug use, likely due to degradation concerns. As reported, the degradation process occurs in an acidic solution, which cannot be continuously maintained in downhole conditions. A potential solution could be the addition of an acidic catalyst; however, this presents challenges due to the high reactivity of the catalyst with the amine group.

### 4.5. Other Potential Chemistries

#### 4.5.1. Urea–Formaldehyde Resins

**Figure 30:**
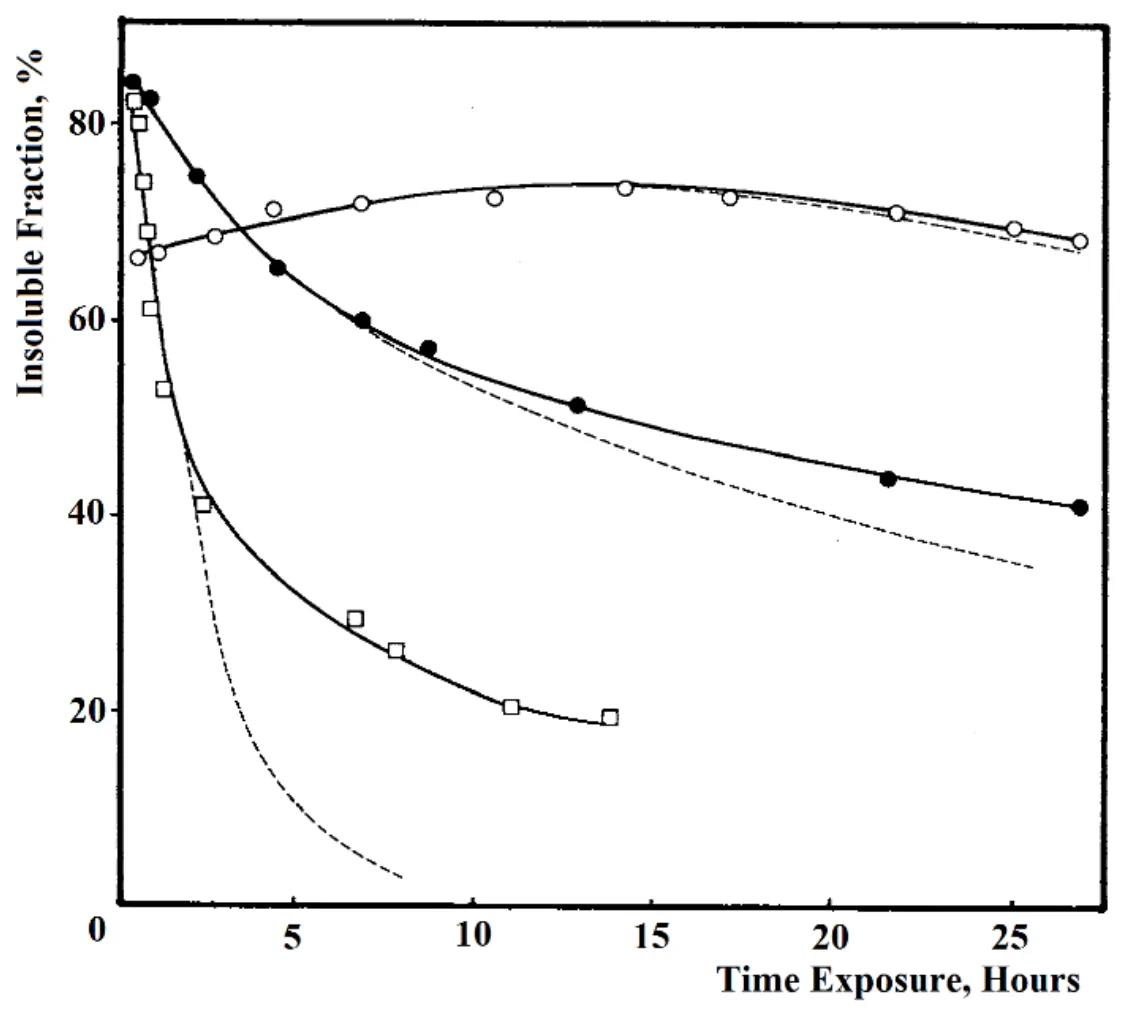
A comparison of the experimental results and the theoretical values (dotted lines) for the amount of the insoluble fraction in cured UF resin suspended in water in acid conditions (pH 4.0) and heated at temperatures of 47 °C (o), 80 °C (•), and 97 °C (□) for various periods of time [65].

Urea reacting with formaldehyde forms the classic urea–formaldehyde (UF) polymer [64], a crosslinked material that degrades over time. This degradation can be accelerated by incorporating a catalyst into the polymer matrix. UF resin, despite its favorable mechanical properties (high strength and modulus), is relatively brittle compared to other resins like epoxy. It has been widely used as a matrix material in wood fiber composites. However, its tendency to degrade has led to its ban in insulation foam applications in many countries due to environmental and health concerns. On the other hand, the inherent instability of urea–formaldehyde linkages could be advantageous for developing degradable polymers for downhole applications, where environmental or health concerns are less critical. Dutkiewicz [64] studied the hydrolytic degradation of cured UF resin, demonstrating relatively rapid hydrolysis in acidic solutions (Figure 30).

Urea itself is a fascinating feedstock for degradable polymers. As an industrial byproduct and a cost-effective fertilizer that supplies nitrogen to the soil, it represents a valuable resource for innovative materials development. The conversion of urea into value-added degradable polymers is an emerging field of interest. Recently, urea ester polymers have been reported, offering improved degradability and higher melting temperatures compared to corresponding aliphatic polyesters [25].

#### 4.5.2. Schiff Base Bonds

**Figure 31:**
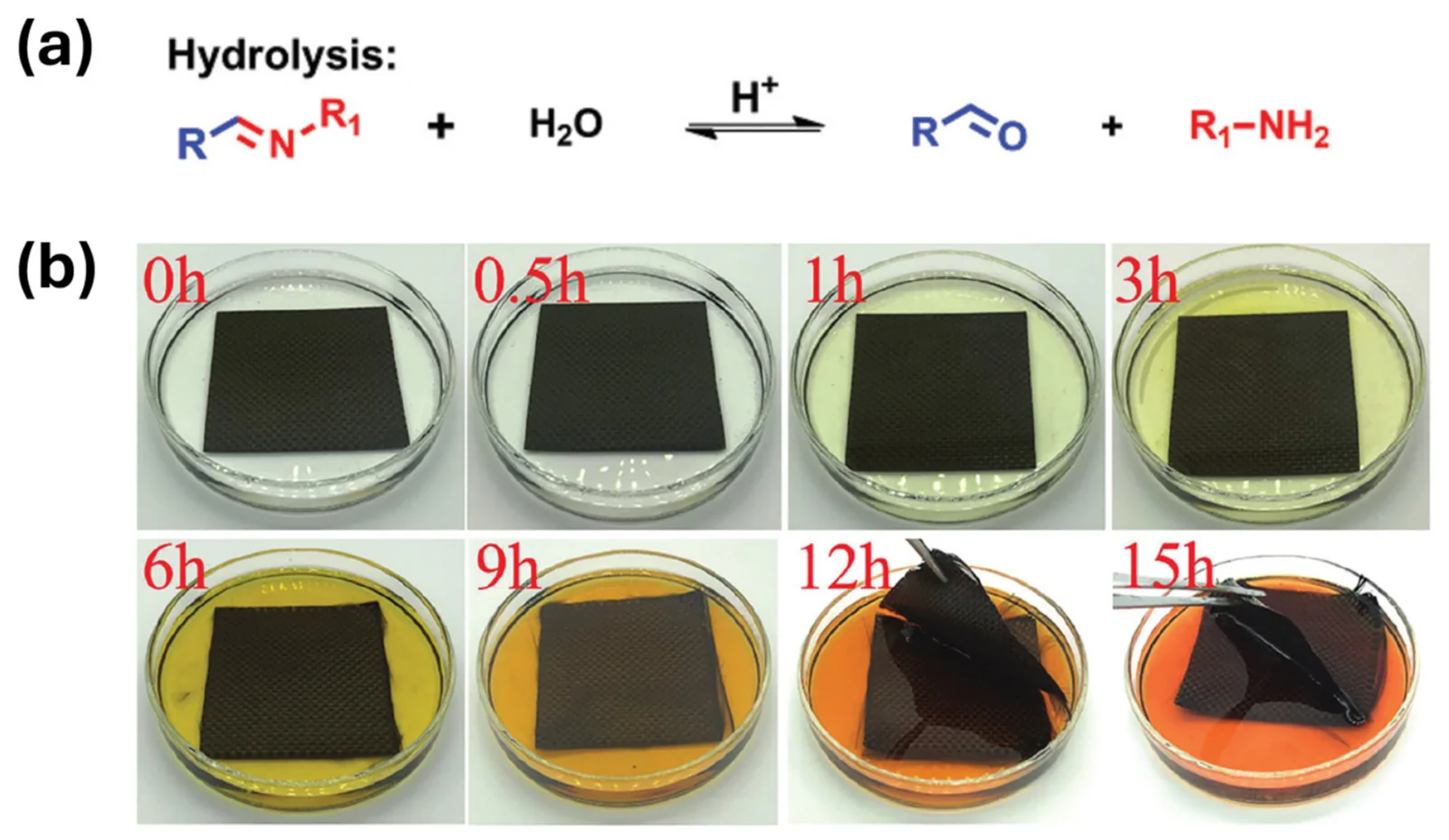
Reversible reactions of the Schiff base linkage (**a**); degradation process of the Schiff base epoxy CFRP with dimensions of 10 cm × 10 cm × 0.45 mm in 0.1 M HCl solution (methanol/H_2_O = 8/2, *v*/*v*) at room temperature (**b**) [66].

The Schiff base, named after Hugo Schiff, is characterized by its imine bond (C=N). It can be synthesized through the condensation of amines with aldehydes or ketones, either with or without an acidic catalyst. This reversible covalent bond can hydrolyze back into aldehydes (or ketones) and amines under acidic conditions and undergo exchange reactions like transimination and imine metathesis, as shown in Figure 31a.

Schiff base epoxy thermosets demonstrate impressive properties due to the π-conjugated structures and associated hydrogen bonding. They exhibit a glass transition temperature (Tg) of 172 °C, a tensile strength of 81 MPa, a tensile modulus of 2112 MPa, a thermal degradation temperature (Td5%) of 323 °C, and an elongation at break of 15%. These properties are comparable to or exceed those of high-performance bisphenol A epoxy resins like Dow Chemical’s DER331 [66]. Carbon fiber-reinforced polymers (CFRPs) made from this resin have a tensile strength of 763 MPa, a tensile modulus of 35.3 GPa, and an elongation at break of 3.02%, matching the performance of DER331-based CFRPs. Additionally, this resin can completely degrade at room temperature under mild acidic conditions (0.1 M HCl in a methanol/H_2_O solution, 8/2 *v*/*v*), as shown in Figure 31b.

Owing to its simple preparation process, excellent performance, and good stability, the Schiff base resin has been commercialized by companies like Mallinda (Denver, CO, USA). However, its degradation behavior under downhole conditions has not been tested, and the cost-effectiveness at an industrial scale remains unclear. This material shows promise as a technical route for downhole dissolvable composite applications, warranting further investigation.

#### 4.5.3. Glycerol-Based Thermosetting Polymers

**Figure 32:**
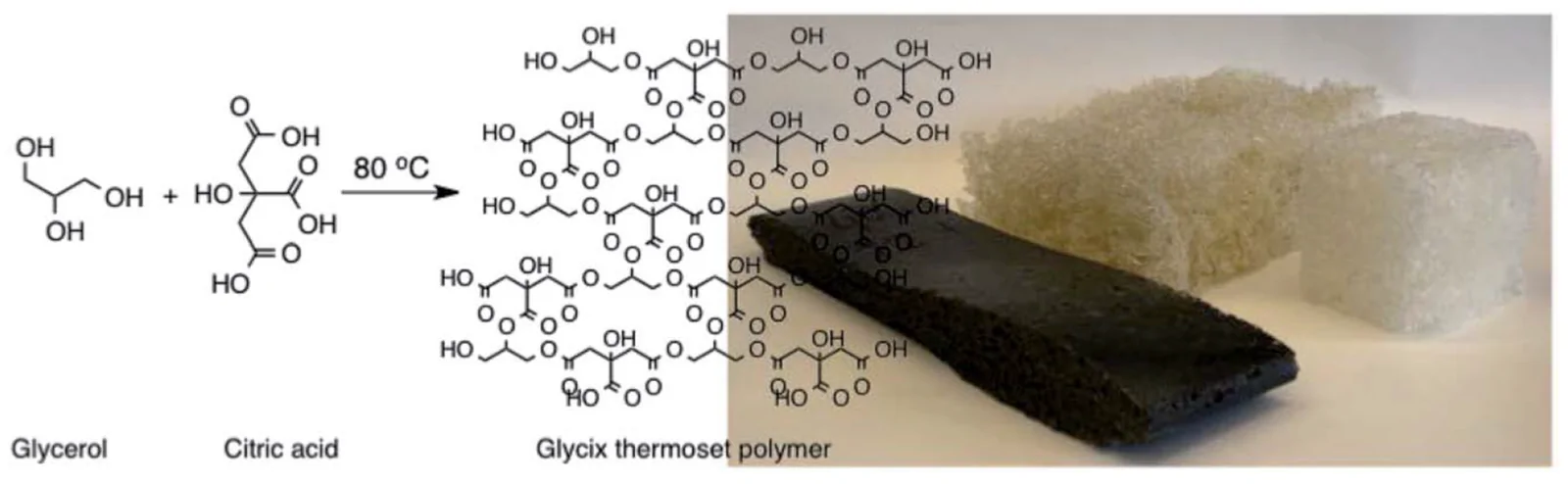
The chemical reaction between glycerol and citric acid gives a 3D thermoset network polymer [69].

Glycerol is a polyol with three hydroxyl groups (Figure 32), and in the form of glyceride—a tri-ester derived from condensation with a carboxylic acid—it is prone to hydrolysis. Due to its tri-functionality, glycerol can be engineered into a crosslinker for diacids, polyanhydrides, and polyurethanes. One example is the crosslinked polyester formed by the condensation of glycerol with citric acid (Figure 32) [67]. This reaction has been known for some time, but a team in the Netherlands recently worked to promote this crosslinked polyester for commercial applications under the name “Glycix” [68].

Drs. Rothenberg and Alberts from the University of Amsterdam, the inventors of Glycix, claimed it to be “the only thermoset plastic that is both biobased and biodegradable”. They suggested that all inflexible plastic items used in homes and buildings could be made from this polymer, such as computer and telephone casings, insulation foam, trays, tables, and lamps. They also stated that Glycix could be injection-molded, although very limited information is available from related web searches. Before the term “Glycix” was coined, other groups, including one from Oregon State University, had studied a similar polyester [68]. In a master’s thesis, Trenkel-Amoroso examined the synthesis, degradation, and potential applications of this crosslinked polyester. Notably, it degrades rapidly in water, especially at high temperatures. For instance, degradation at 110 °C in water can occur within a few hours or days, depending on the crosslinking density of the polymer. However, a major challenge in processing such a crosslinked polyester is water management. Water is produced during condensation, and the temperature for effective condensation is well above the boiling point of water. As a result, foamed materials are often produced, and multi-stage synthesis may be needed for better water management. However, little information is available in the open literature, and detailed thermal and mechanical properties are not well documented. Similarly to the dilemma discussed in the epoxy system, no aromatic groups exist in the polymer chain in these technologies. Regardless of the extent of crosslinking, the glass transition temperature (T_g_) is still limited. The reported work on this material generally falls below 100 °C [67], restricting its use to low-temperature applications.

Glycerol is widely available as a major byproduct of biodiesel production. The continued growth of the biofuel industry is expected to maintain a substantial supply of glycerol; global biodiesel production is projected to reach approximately 79 billion liters by 2033 [70]. Moreover, crude glycerol often contains significant amounts of toxic chemicals, such as methanol. Therefore, this material could potentially serve as a highly cost-efficient raw material for developing degradable materials for use in downhole conditions, where toxicity is not a concern.

In addition to the three chemistries discussed earlier, several other novel linkages demonstrate potential for hydrolysis and degradability, including hexahydrotriazine linkage bonds [71], boronic ester linkage bonds [72], and disulfide bonds [73]. These unique chemistries offer intriguing possibilities for engineering degradable materials. However, these technologies remain in the academic stage, with limited information available regarding key factors such as cost, scalability, and potential market size. The lack of detailed data makes it challenging to assess their commercial viability or readiness for industrial deployment. Further research and development are necessary to explore the full potential of these chemistries, optimize their properties, and adapt them for specific applications, including downhole use in the oil and gas industry. This could unlock new opportunities to leverage their unique degradation mechanisms in demanding operational environments.

Unlike thermoplastic polymers, thermoset polymers are typically brittle, especially those with a high crosslinking density designed for high-temperature applications. To mitigate this issue, the industry has invested significant resources in thermal molecular design, incorporating toughening agents, using active diluents, and introducing solid phases in various forms such as particles, spheres, and short or continuous fibers. These strategies can significantly enhance mechanical performance; however, their impact on dissolvability is theoretically evident but has not been extensively studied. In fact, for the epoxy system discussed earlier, the raw material was selected from a commercially available epoxy grade that already included a proprietary toughening agent [7]. In other words, the base epoxy and toughening agent were considered as raw materials, with the catalyst added being based on overall performance requirements. Therefore, further research on toughening agents is necessary if current commercial thermoset grades fail to meet future demands.

## 5. Dissolvable/Degradable Rubbers

The water-dissolvable elastomer sealing element is an essential component of the dissolvable tools [74]. The dissolvable elastomer needs to keep a good elasticity for a certain time period, such as more than 12 h, to complete the fracturing operation. On the other hand, the dissolvable elastomer needs to be dissolved as fast as possible after performing the fracturing operation. It is very challenging to develop the dissolvable rubbers to satisfy both requirements. The polymer backbones for the traditional rubbers, such as natural rubbers, nitrile rubber, fluororubbers, etc., are not water-soluble. Even though the dissolvable plug has been used in the industry for more than 15 years, there are still limited publications on the development of dissolvable rubbers. Scientists have tried different methods to develop dissolvable rubbers.

### 5.1. Development of Dissolvable Rubbers

At the early stage, researchers developed methods to compound dissolvable polymers with traditional rubber, such as nitrile butadiene rubber (NBR) or hydrogenated nitrile butadiene rubber (HNBR). Sherman invented a dissolvable rubber sealing material by blending dissolvable polymers, such as PGA, poly(vinyl alcohol) (PVA), polyethylene glycol (PEG), poly(lactic acid) (PLA), etc., with traditional elastomers such as natural rubber, silicone, NBR, etc. [75]. It is quite challenging to control traditional rubber as an isolated phase in the dissolvable polymer matrix after vulcanization.

**Figure 33:**
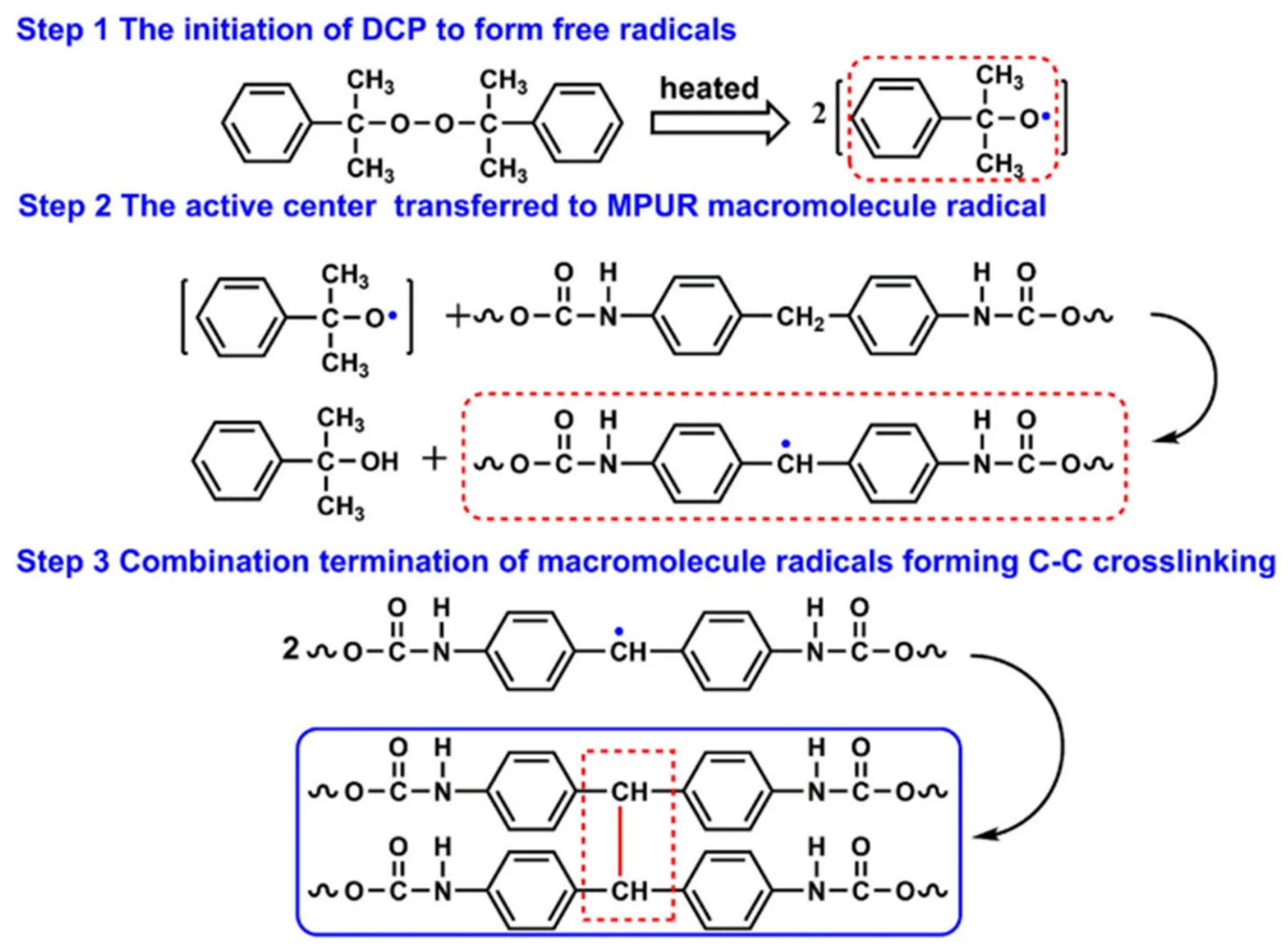
Schematic of MPUR crosslinking process [76].

Cheng et al. designed a dissolvable sealing material by blending millable polyurethane rubber (MUPR) and HNBR (Figure 33). MUPR has good tensile strength, tear strength, and wear resistance compared with traditional rubber. However, when it is used in the high-temperature water medium, its groups such as urea formate, biuret, ester, ether, and so on are easily hydrolyzed, which easily causes the quick failure of processed seals when the fracturing process is not completed. Therefore, the researcher tried to control the dissolution rate of MUPR by blending HNBR.

**Figure 34:**
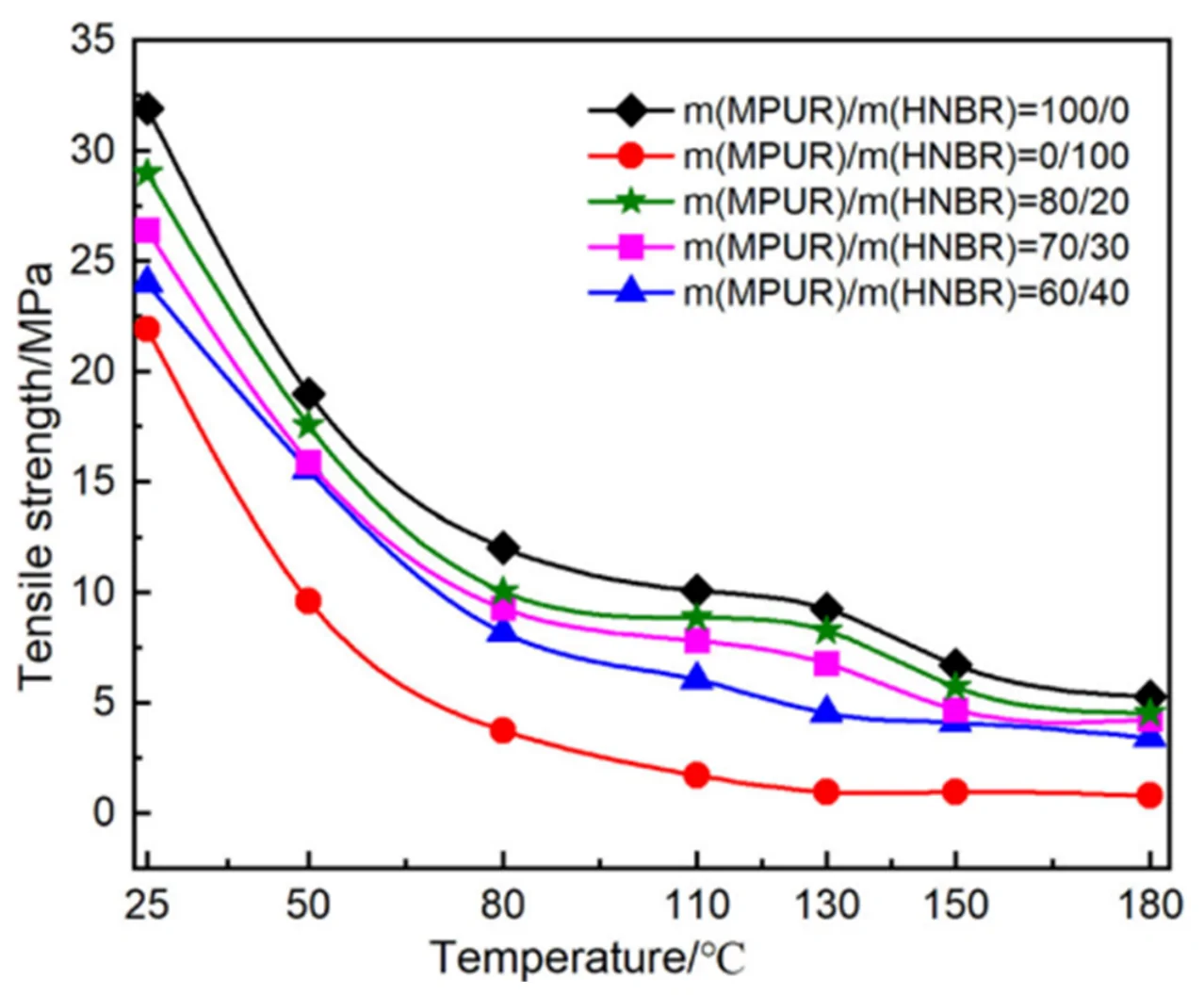
The effect of temperature on tensile strength for degradable rubber composites [76].

Dicumyl peroxide (DCP) was used as a crosslinking agent. DCP decomposes thermally and produces a free radical. The free radicals attack polymer molecular chains to capture a-H and form macro-molecular radicals, while a couple of free radicals will further form the crosslinking bond. The effect of HNBR content on the mechanical properties of MPUR/HNBR composites is shown in Table 7. It was found that by increasing the HNBR amount, the tensile strength of MPUR/HNBR composites slightly decreased, while the 100% modulus increased slightly. The effect of the temperature on tensile strength for MPUR/HNBR composites is shown in Figure 34. It was found that with the increasing temperature, the tensile strength of all materials decreased significantly.

**Figure 35:**
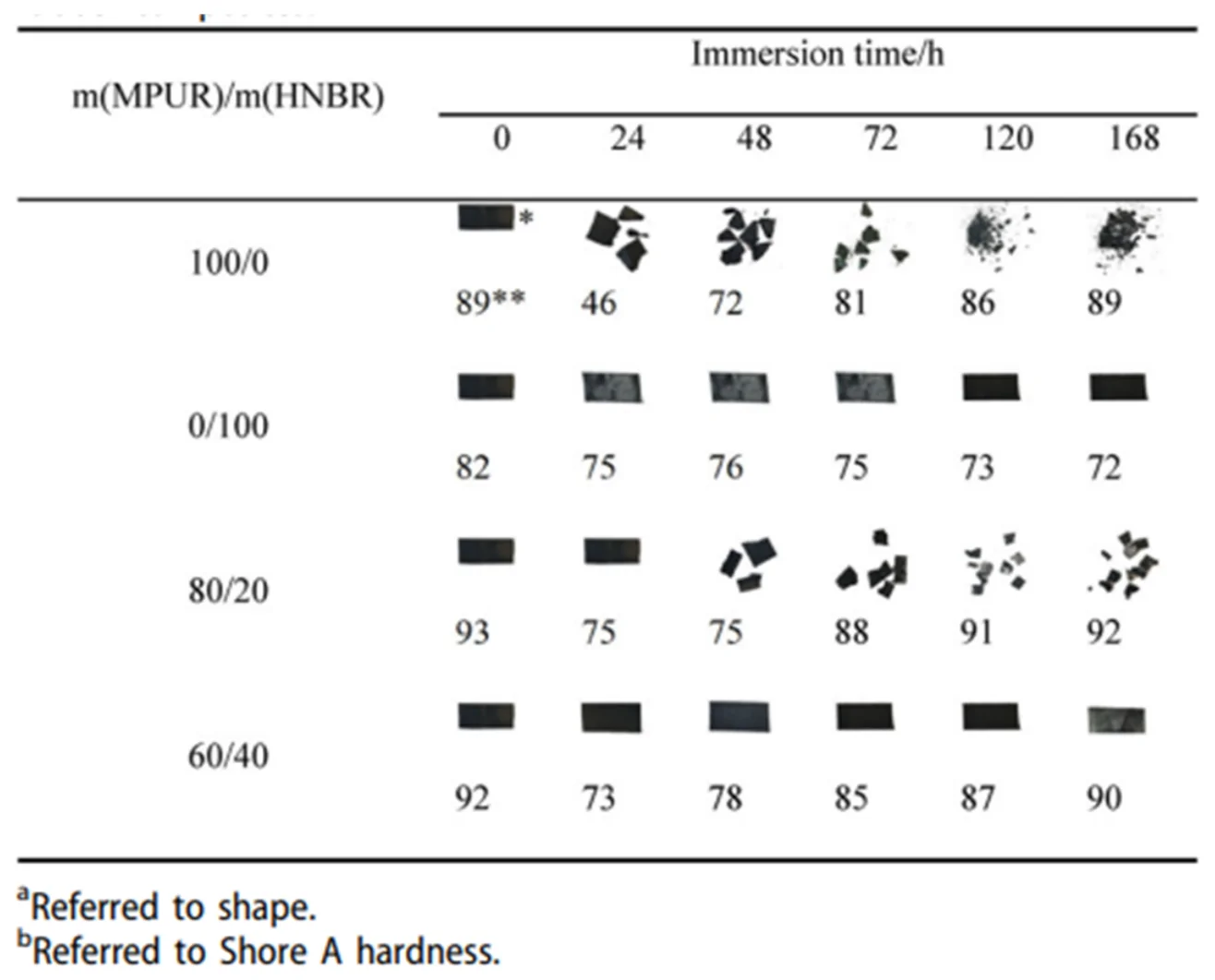
Effect of immersion time on the shape and hardness of degradable rubber composites [76]. Note: a = *; b = **.

The dissolution properties of the MPUR/HNBR composites were studied by comparing the mechanical properties before and after immersion in water at 100 °C. The dissolution of the composite was mainly due to the hydrolysis of the MUPR material. Based on the experimental results studied above, it can clearly be seen that the soft segment domains of MPUR undergo a hydrolysis reaction, which plays a vital role in the degradation of degradable rubber materials. It is concluded that the degradation of MPUR materials in a 100 °C water medium will degrade the soft segment domain and dissolve a small amount of the hard segment domain. The effect of immersion time on the shape and hardness of MPUR/HNBR composites is shown in Figure 35. The hardness of the pure MPUR composite first decreased rapidly and then slowly increased to its initial hardness; meanwhile, the composite was broken into lumps after being immersed for 24 h and broken into granules after being immersed for 72 h. The hardness of the MPUR/HNBR composite material reaches the lowest point after being immersed for 24 h, and the composite starts to break after being immersed for 72 h.

**Figure 36:**
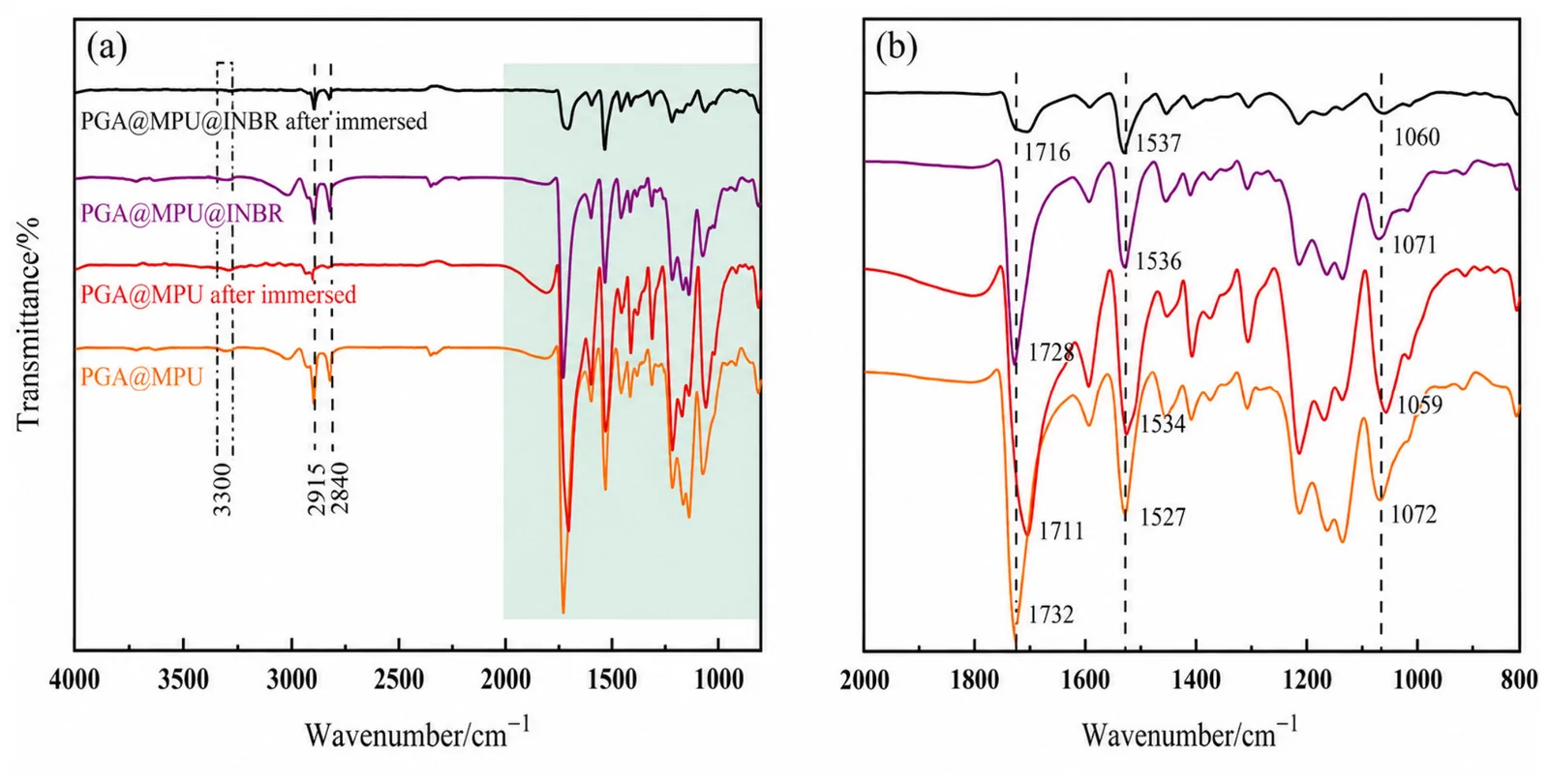
FT-IR spectra. (**a**) Infrared spectra of the PGA@MPU/HNBR composite materials before and after immersion; (**b**) partial enlargement [77].

Cheng et al. also added PGA to the MPUR/HNBR compound to increase the dissolution rate [77]. It can be found from the experimental results that the tensile strength of the PGA@MPU composite material significantly dropped when compared with the blank MPU material without adding PGA. This was because the PGA with a low molecular weight was a brittle material, which was partially compatible with the soft segment of the MPU. PGA contributed to enhanced hydrolytic degradation and a reduction in material hardness. Hydrolysis primarily affected the ester groups in both PGA and MPU, with the complete degradation of MPU ester groups within 24 h and PGA ester groups degrading over approximately 72 h. The FT-IR of the PGA@MPU composite material and PGA@MPU/HNBR composite material before and after degradation in water at 100 °C is shown in Figure 36. The absorption peak at 1732 cm^−1^ was the stretching vibration of C=O in the ester group. After immersion and degradation, part of the urethane underwent a hydrolysis reaction. The position of the C=O vibration was blue-shifted, the PGA@MPU composite material was reduced from 1728 cm^−1^ to 1716 cm^−1^, and the PGA@MPU/HNBR composite material was changed from 1732 cm^−1^ to 1711 cm^−1^. However, no publication has reported on the pressure rating and dissolution performance of the sealing element made of either MPUR/HNBR or PGA@MPU composites yet.

Modified polyurethane has been reported as a dissolvable rubber. By grafting polyacrylamide hydrophilic chains [78], or by grafting dihydroxymethylpropionic acid chains [79], the polyurethane main chain possesses a certain degree of rubber-like elasticity and strength, while the grafted polyacrylamide chains or grafted dihydroxymethylpropionic acid chains exhibit water-soluble properties. Dissolvable rubbers with different temperature ratings have been reported for various formations in the world.

### 5.2. Low-Temperature Dissolvable Rubbers

**Figure 37:**
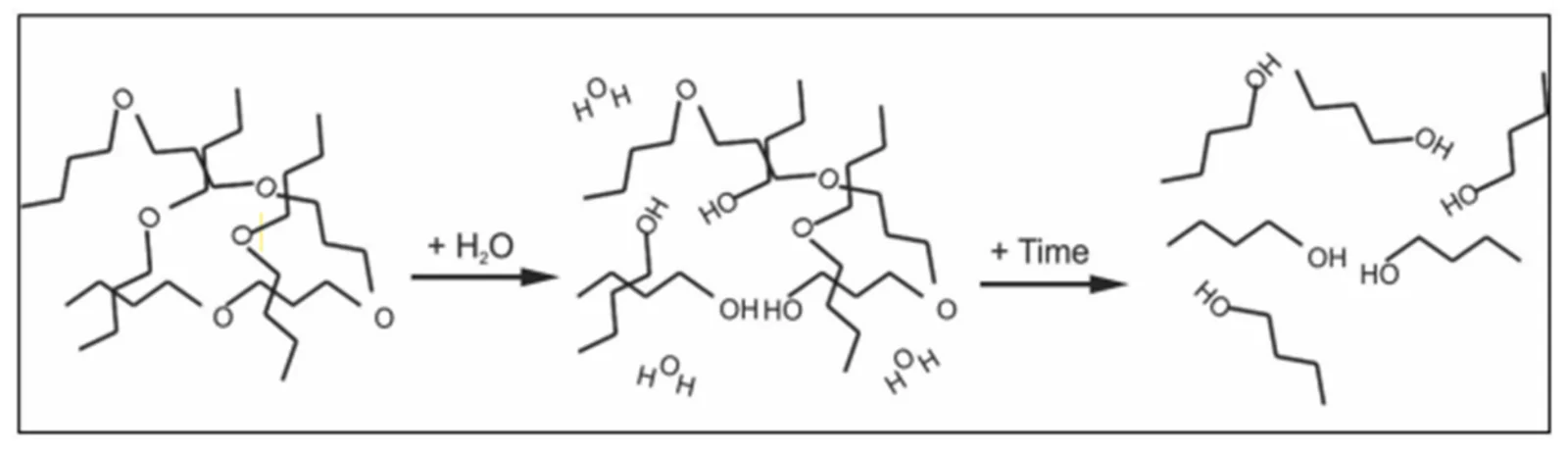
Water breaks the bonds at active groups in the polymer chains, causing the elastomer to weaken and fall apart [81].

**Figure 38:**
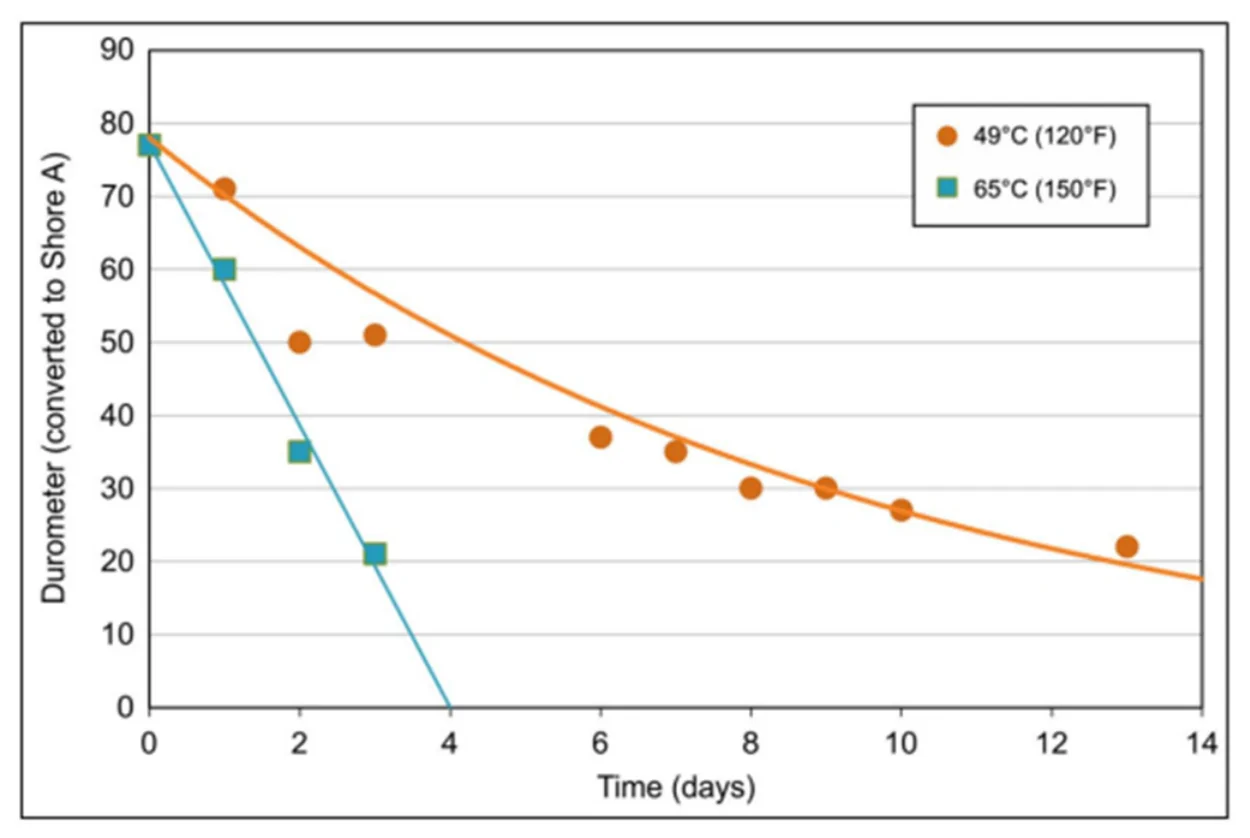
Degradation of low-temperature elements with a medium concentration of crystalline accelerant at 65 and 49 °C (150 and 120 °F) [81].

The Permian Basin in West Texas produces approximately 2 million b/d and accounts for over 20% of US crude production. [80] The formation temperature is low for the Permian Basin and some other US gas shale reservoirs, such as Marcellus and Fayetteville. The average downhole temperature for these formations ranges from 100 to 150 °F (38 to 66 °C). The dissolution process slows in lower-temperature shale formations, and the elastomer often ceases degradation at these lower temperatures. Fripp et al. designed a low-temperature dissolvable rubber for low-temperature wellbores [81]. For the development of a dissolvable elastomer, water-sensitive functional groups were added to the elastomer to aid hydrolytic degradation. A low-temperature elastomer was developed by adding a crystalline accelerant into the base degradable polymer. The crystalline accelerant combines swellability and water absorption into the elastomer. During hydrolytic degradation, the accelerants physically rip apart the polymer. The crystalline accelerant had minimal interference with the curing process but reduced the tear strength of the low-temperature elastomer (Figure 37). The degradation rate is approximately an Arrhenius-type temperature dependence and is a function of temperature. Decreasing the temperature leads to a decrease in the degradation rate of the low-temperature degradable elastomer (Figure 38).

**Figure 39:**
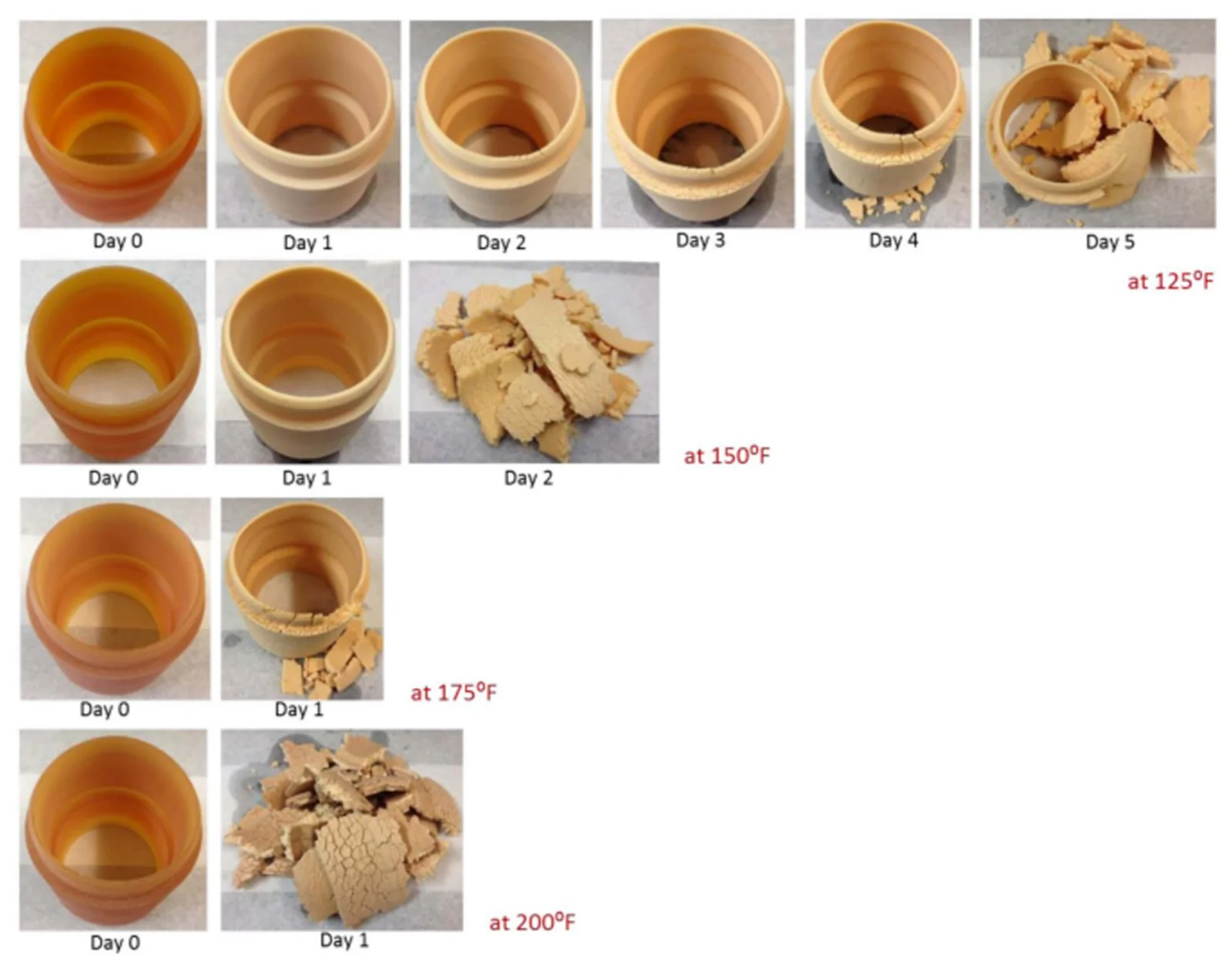
Degradation of low-temperature packing element in the light brine [80].

Duan et al. developed a low-temperature dissolvable rubber sealing element [80]. The element is aged in the light brines at different temperatures and for different durations (Figure 39). At 125 °F, the element begins to show signs of cracking within two days. The degradable frac plug effectively maintains a 10,000 psi pressure differential for approximately seven hours—two hours at 150 °F, four hours at 200 °F, and about one hour during the temperature transition from 150 °F to 200 °F.

### 5.3. Medium-Temperature Dissolvable Rubbers

**Figure 40:**
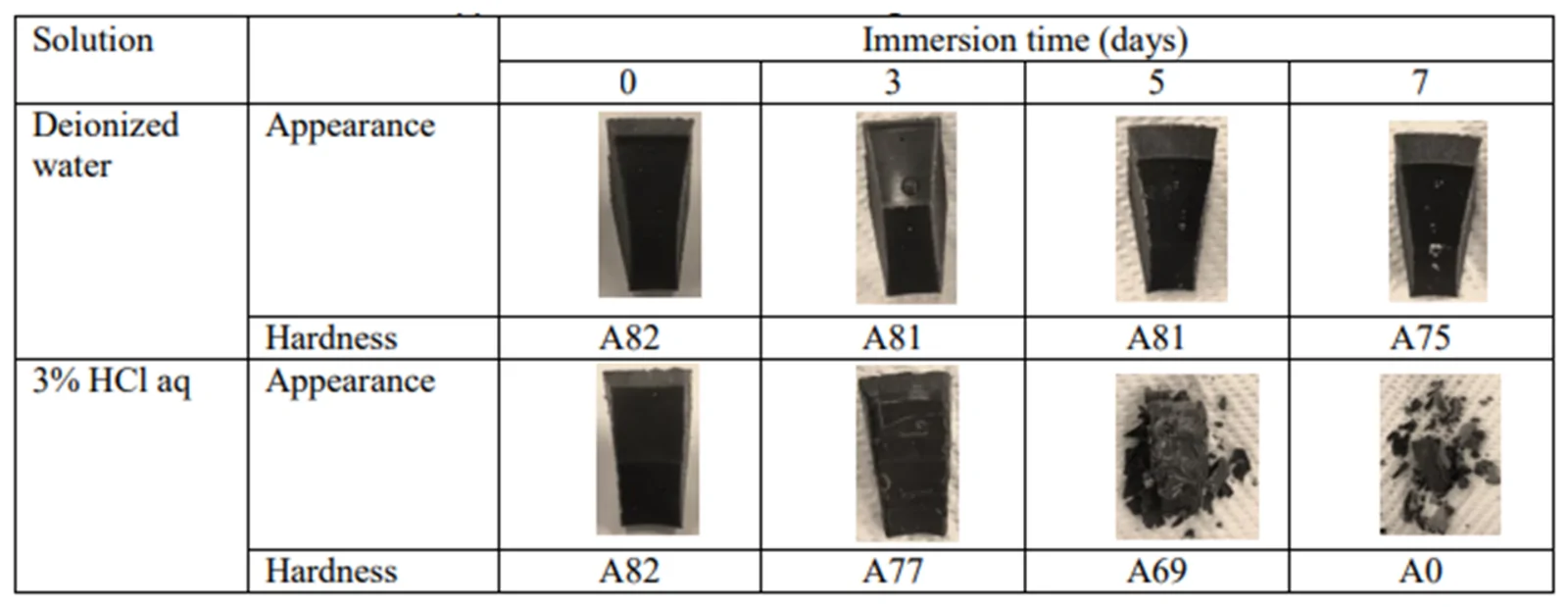
Appearance and hardness of degradable rubber at 176 °F [74].

The temperature is in the medium range, 80 to 120 °C, for the formations such as the Barnett basin in the US and Changning in China. Takahashi et al. reported a degradable rubber with the mechanical strength comparable to that of conventional nitrile butadiene rubber (NBR) [74]. Figure 40 shows the changes in appearance and the durometer hardness of the degradable rubber that has an initial hardness of A82 during immersion in DI water and in aqueous 3% HCl at 176 °F. Although the hardness of the degradable rubbers decreases with immersion time in DI water at this temperature, the rate is slow. In 3% HCl, the degradation proceeds faster than in DI water.

**Figure 41:**
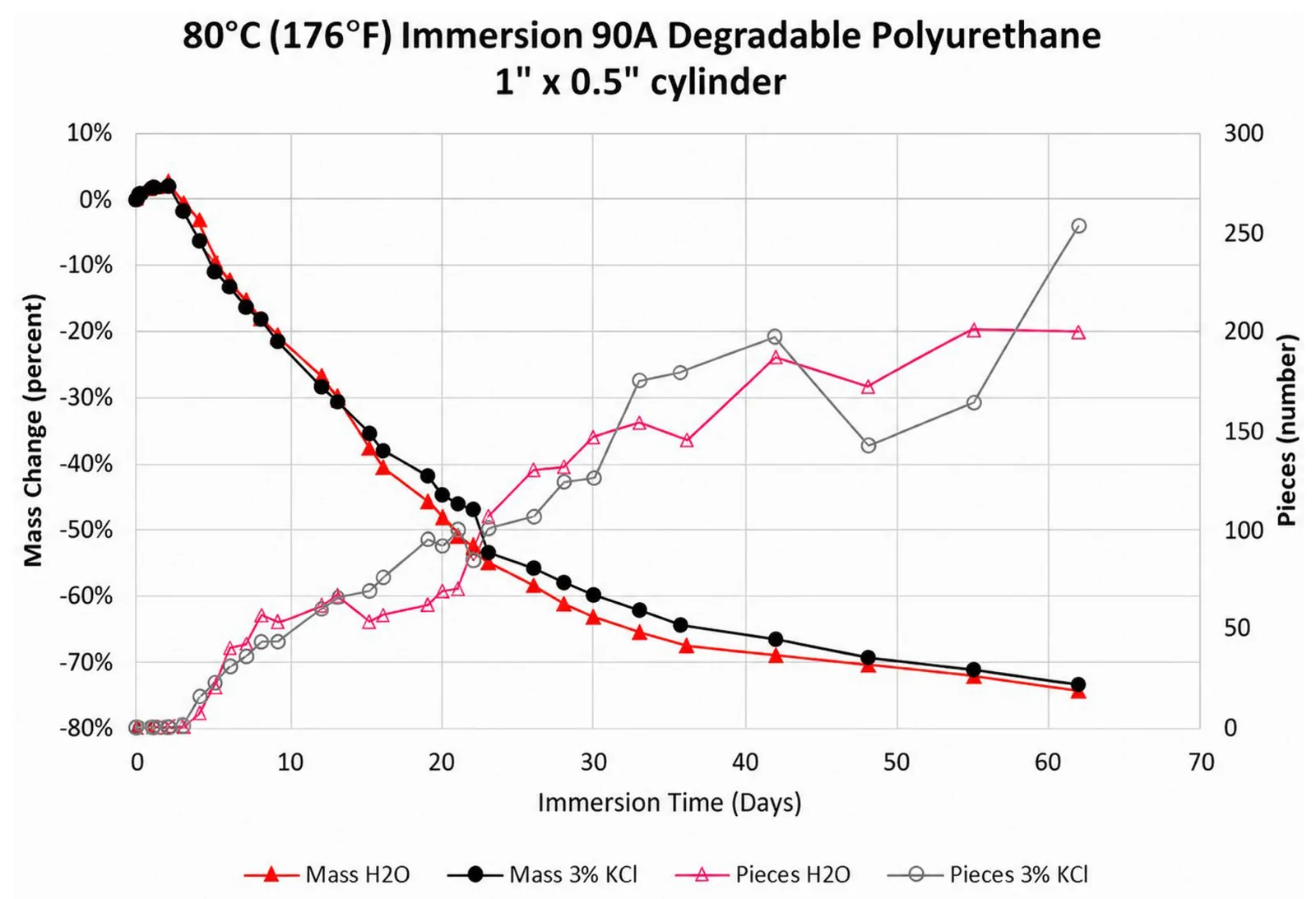
Degradation profile aqueous immersion at 80 °C (176 °F) [82].

Burdzy developed polyurethane elastomers for medium temperatures [82]. The compression modulus decreased 12 to 55 percent depending on the curative after 6 h immersion in 3% KCl at 80 °C (176 °F). Figure 41 shows the mass change as a function of immersion time. In total, 40% of mass was lost after 20 days. It seems that the dissolution rate is low.

Xu et al. reported the testing results on a water-dissolvable rubber element based on thermosetting polyester polyurethane [83]. The sealing element, based on a slow dissolvable rubber and a fast dissolvable rubber, passed a pressure testing of 70 MPa at 120 °C in a heat transfer oil medium. The fast dissolvable rubber element was completely dissolved into small pieces in 7 days in 1% KCl at 90 °C.

**Figure 42:**
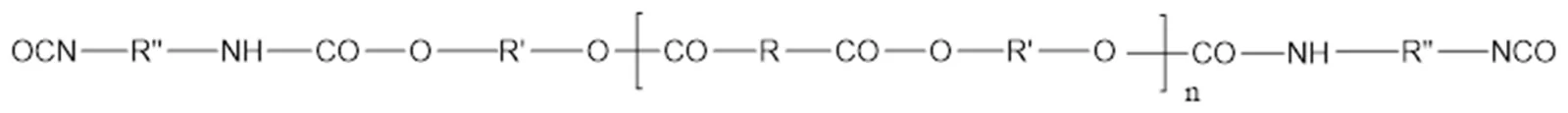
Molecular structure of polyester polyurethane.

Ren et al. developed high-strength fast dissolvable medium-temperature dissolvable rubbers [84]. The dissolvable rubber is based on a polyester polyurethane rubber as shown in Figure 42. The soft segment, hard segment, and crosslinker were designed to make the dissolvable rubber have high strength, high elongation, and high tear strength, as well as good dissolution properties.

**Figure 43:**
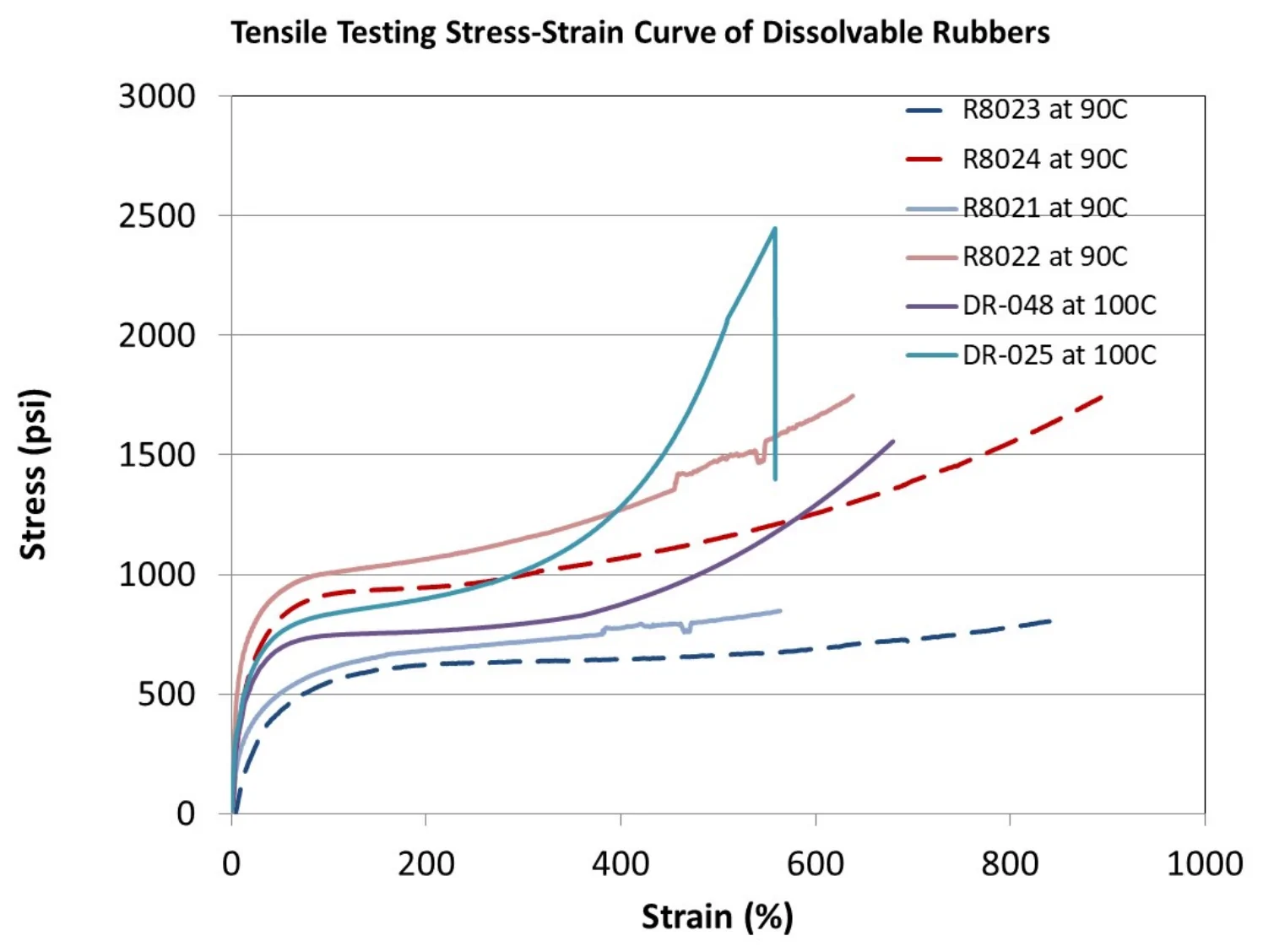
Tensile properties of dissolvable rubbers at 90 °C and 100 °C [84].

**Figure 44:**
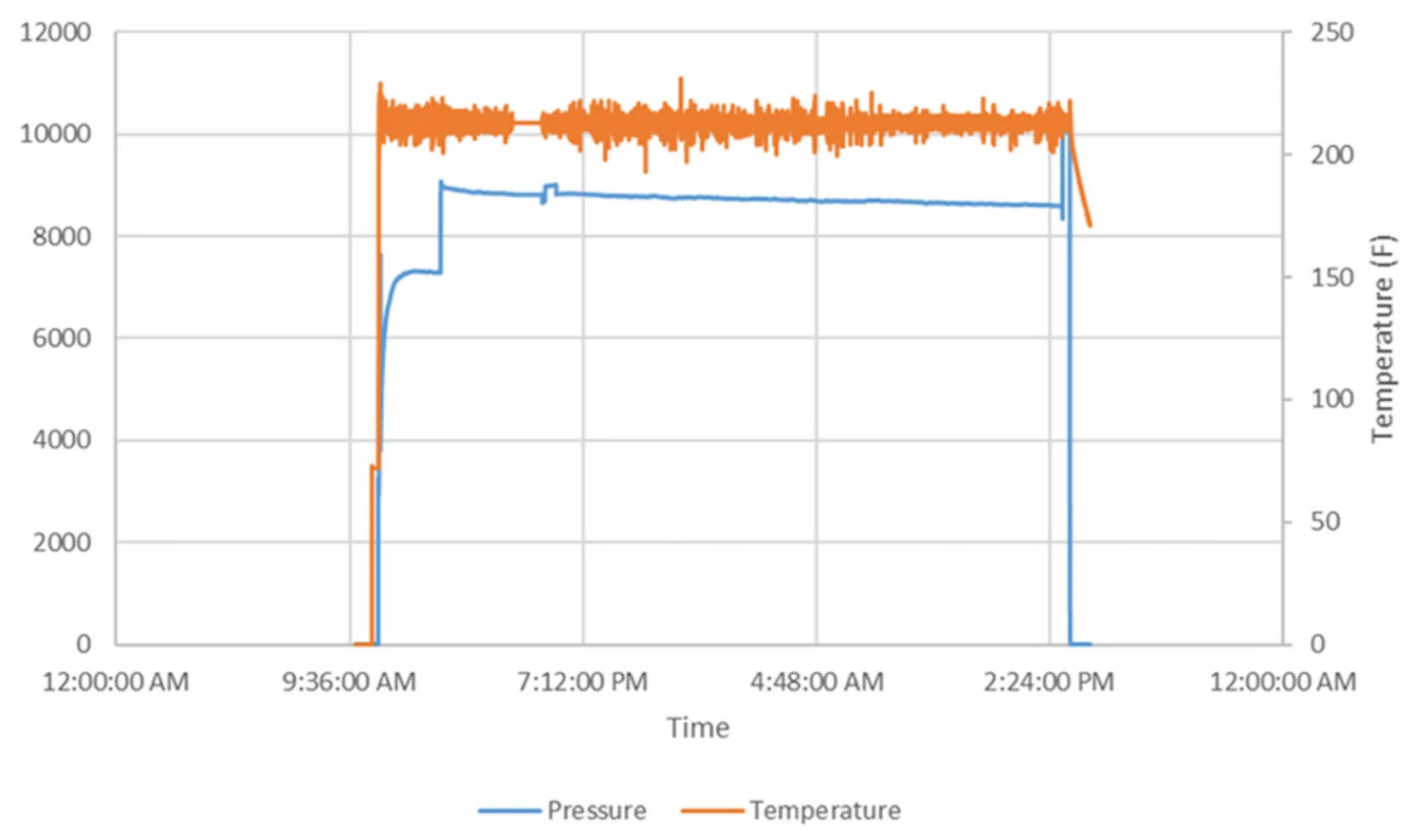
The pressure-holding testing results of dissolvable rubbers made of DR-025 at 100 °C in a water environment [84].

**Figure 45:**
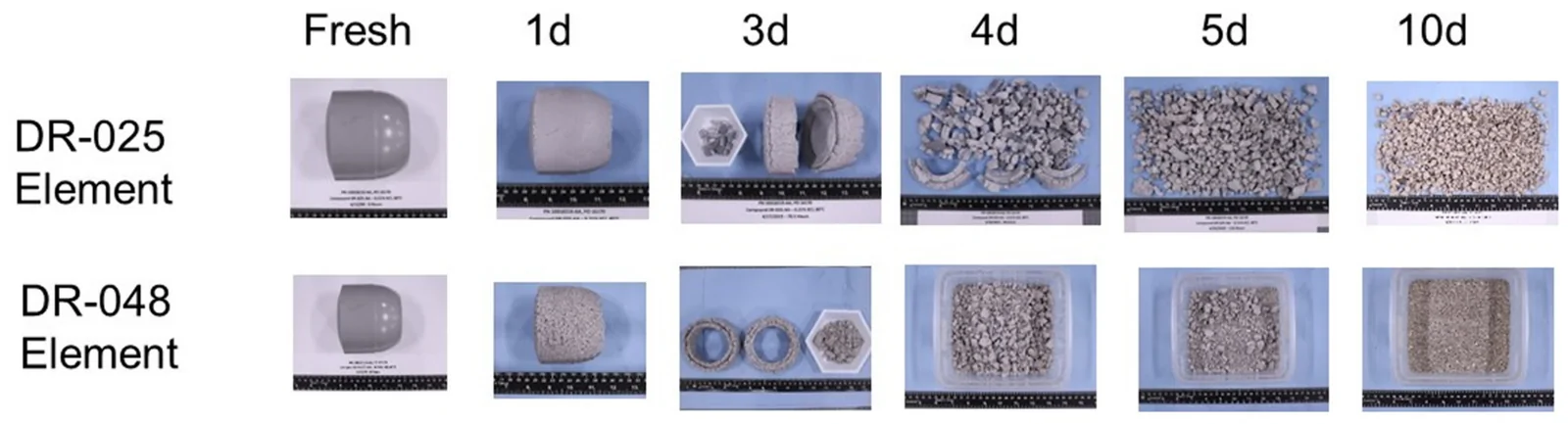
The dissolution testing progress of DR-025 and DR-048 elements at 80 °C in 0.21% KCl (first 5 days) and 1.05% KCl after 5 days [84].

The tensile strength of DR-025 and DR-048 (two grades discussed in this paper) exceeds 1500 psi at 100 °C. The elongation of both DR-025 and DR-048 was higher than 550% at 100 °C (Figure 43). The results suggested that the sealing element made of DR-025 and DR-048 could hold 10,000 psi at 100 °C. Figure 44 shows the pressure rating test results of the dissolvable rubber sealing element made of DR-025 in water at 100 °C. The sealing element held an 8700 psi pressure differential very stable for 24 h and then 10,000 psi for 15 min. The sealing element made of DR-048 also passed the pressure rating testing at 100 °C in water. Figure 45 shows the dissolution process of the dissolvable rubber sealing element made of DR-025 and DR-048 at 80 °C in KCl solution. The sealing element made of DR-025 and DR-048 broke into small and soft pieces in 5 and 4 days, respectively. The dissolvable rubber residues could easily flow back during operation. These dissolvable plugs have been used successfully in many oilfields in China.

### 5.4. High-Temperature Dissolvable Rubbers

There are more and more HT wells in the southwest of China, which require high-temperature dissolvable plugs in recent years. To meet the Southwest Oilfield Company lab test requirements, the HT dissolvable plug needs to hold a 70 MPa pressure in water at 150 °C for 24 h. On the other hand, the dissolvable plug needs to be dissolved in 1% KCl at 95 °C in less than 15 days. These requirements place seemingly mutually exclusive challenges on dissolvable materials. Norman et al. developed dissolvable metal and dissolvable elastomer for high-temperature formation in the Middle Eastern basin [85]. However, the typical high temperature was only 121 °C. The metal–metal seal dissolvable plugs were reported to be used in the southwest of China. However, the metal seal could not make a good seal due to its limited elasticity, especially in the Southwest Oilfield of China. Casing deformation wells are very common in the Southwest Oilfield wells. Significant erosion of the casing has been reported for the location connected with the metal seal of the plug during fracturing.

**Figure 46:**
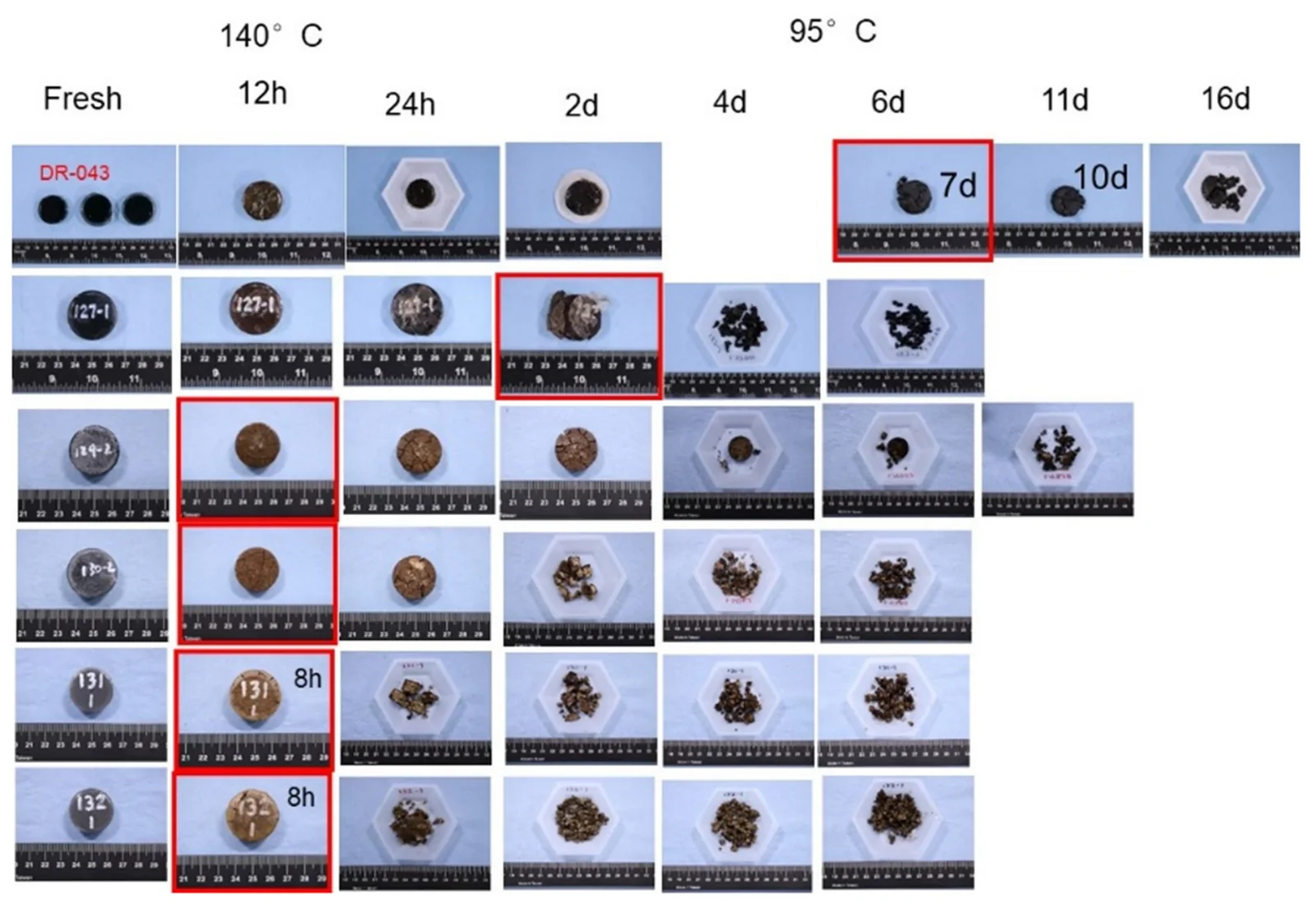
Dissolution progress of HT dissolvable rubbers at 140 °C and 0.3% KCl for 24 h and then at 95 °C and 0.3% KCl for 15 days [86].

**Figure 47:**
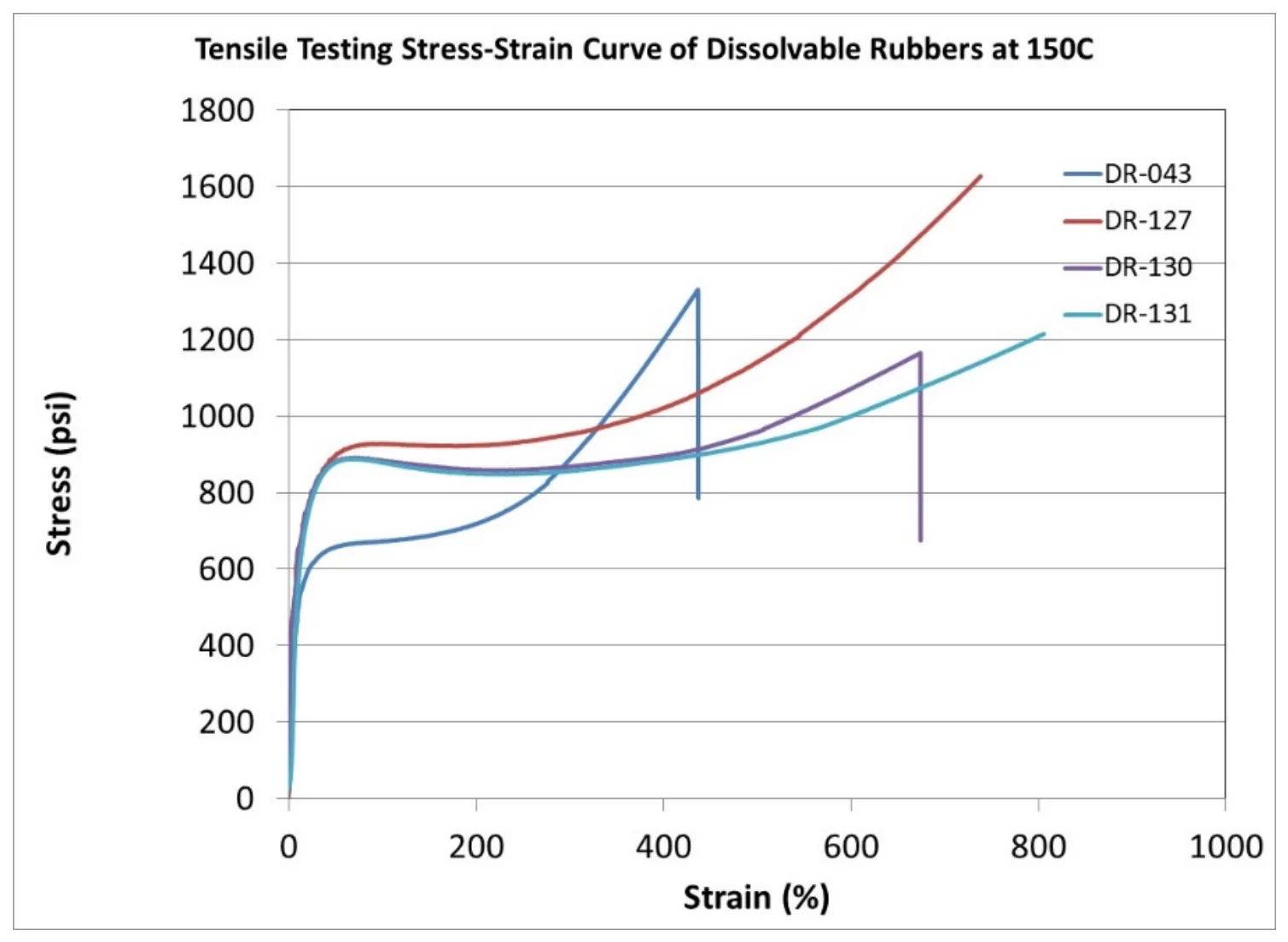
Stress–strain curve of HT dissolvable rubbers at 150 °C [86].

**Figure 48:**
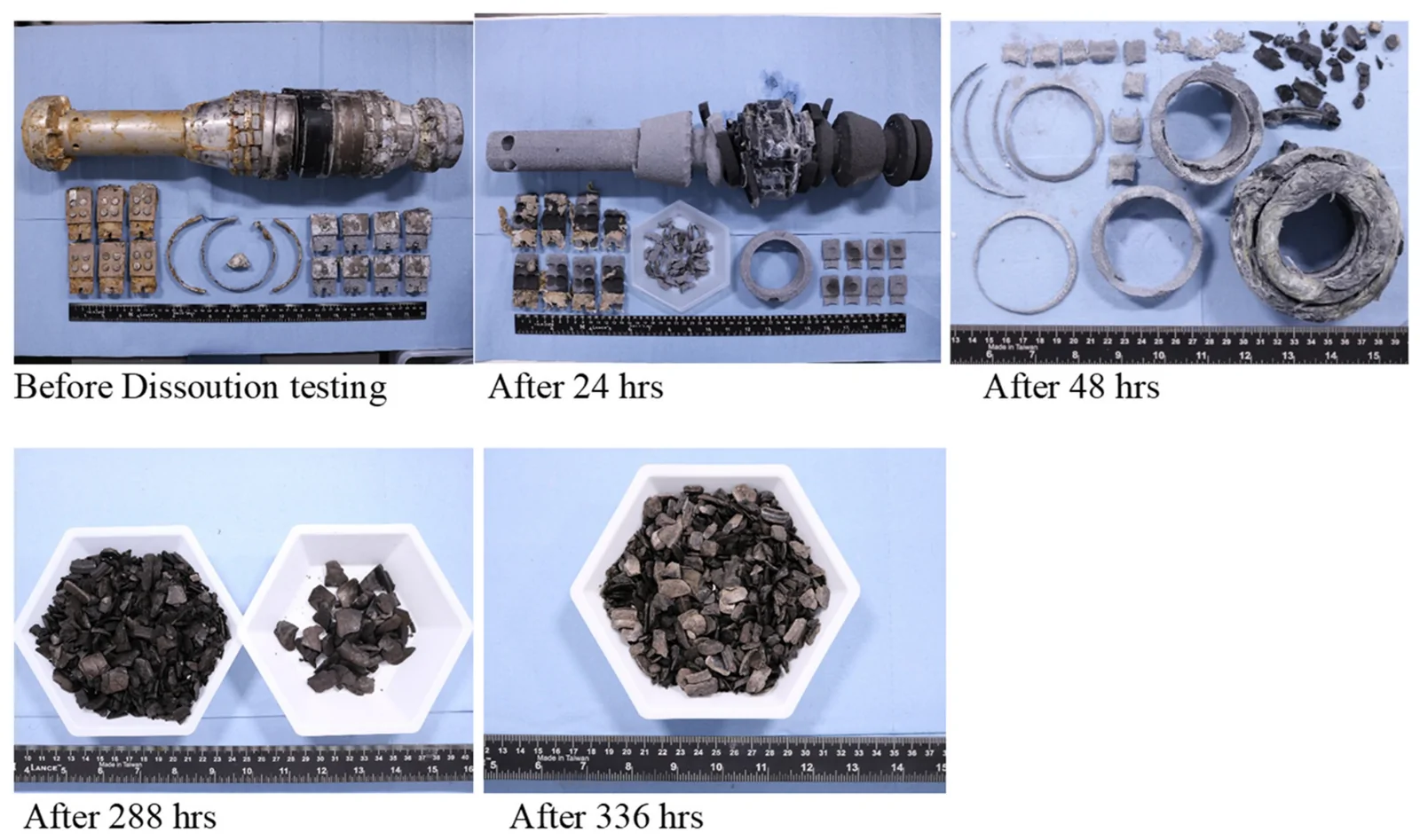
Dissolution testing of HT dissolvable plug at 95 °C and 1% KCl [86].

To meet the industrial challenges, Ren et al. developed a high-temperature dissolvable rubber [86]. In order to let the dissolvable rubber element hold pressure at 150 °C for 24 h, a special polymer coating was developed to delay the dissolution of the HT dissolvable rubber at 150 °C. [87]. Figure 46 shows the dissolution progress of several HT dissolvable rubber composites at 140 °C, 0.3% KCl for 24 h and then at 95 °C, 0.3% KCl for 15 days. The DR-127 composite was fractured after 2 days, and the composite broke into pieces after 4 days. The tensile strength of DR-127 at 150 °C was above 1600 psi, and the elongation of DR-127 was higher than 700% at 150 °C (Figure 47). The results suggest that the dissolvable plug with the dissolvable rubber element made of DR-127 is likely to meet both pressure-holding requirements at 150 °C and the dissolution requirements at 95 °C. Two HT plugs were designed with a rubber sealing element made of DR-127. The plug was set first at an ambient temperature, then soaked in water at 150 °C for 12 h. Afterwards, a 10,000 psi pressure-holding test was performed on the plug. The pressure held stable for 12 h. The plug only lost 400 psi during the 12 h holding at 10 ksi. The testing results meet the HT dissolvable plug pressure-holding testing requirements. Figure 48 shows the dissolution progress of one HT dissolvable plug at 95 °C in 1% KCl after a pressure-holding test. The metal parts were completely dissolved in 72 h. The dissolvable rubber element broke into pieces < 2 cm after 336 h (14 days). The total weight loss of the plug was 95.5%. The dissolution testing results meet the HT dissolvable plug dissolution testing requirements. This is the first time in the industry, based on our knowledge, that an HT dissolvable plug passed a 150 °C, 10 ksi, 24 h pressure-holding test in water and then dissolved in brine at 95 °C in less than 15 days.

## 6. Conclusions and Outlook

The transition from conventional metal materials to polymers and polymer composites has been widely adopted due to their superior corrosion resistance, low cost, and lightweight properties. Similarly, dissolvable polymeric materials are gradually replacing traditional dissolvable magnesium alloys, addressing inherent issues such as recrystallization and high sensitivity to acidic environments. This review has systematically explored various degradable polymeric materials, including thermoplastics, thermosets, and rubbers. It covered aspects such as fundamental mechanisms, material design, properties, product performance, cost analysis, and industrial potential in the oil and gas sector. Many of the technologies discussed have already been commercialized, significantly improving operational efficiency and reducing costs.

Each category of polymeric materials offers unique advantages and limitations, making them suitable for specific applications. The current best practice is to design products with the specific characteristics of each material in mind to maximize their benefits. Over time, as further advancements are made, the differences between these materials will narrow. Centralizing material selection into a limited set of options could greatly simplify supply chains and reduce costs across the industry.

Despite significant progress in both industry and academia, challenges remain that require further development. Thermoplastics are an ideal choice due to their minimal residuals (absence of chemically bonded networks) and cost-effectiveness for large-scale manufacturing. However, their temperature rating is typically limited to below 120 °C, restricting their use to lower-temperature well applications. Advancements in molecular design, composite formulations, and manufacturing techniques are urgently needed to enhance their high-temperature performance.

Thermosets exhibit higher temperature ratings and are already field-tested. However, they often leave large residuals after dissolution, which can become problematic, particularly when chopped fibers are incorporated, potentially blocking production flow paths. While dissolvable glass fibers have been introduced, no mature product has achieved a balanced combination of strength and dissolution capability. Further innovation is needed in this area.

Developing dissolvable rubbers that meet both pressure-holding and dissolution requirements remains a significant challenge. Limited research exists on this topic, with most efforts focused on incorporating dissolvable polymers or catalysts into traditional rubbers (e.g., NBR and HNBR), thermoplastic polyurethane, or modified thermoset polyurethane. Temperature-rated dissolvable rubbers for different formations (low: 38–80 °C, medium: 80–120 °C, and high: 120–150 °C) have been developed. However, further work is required to precisely design dissolvable rubbers that balance pressure-holding and dissolution properties, deeply understand the relationship between rubber chemistry, composition, and performance, and develop ultra-high-temperature dissolvable rubbers (180–220 °C) for HPHT wells, as limited knowledge is available to address this need.

The dissolution behavior of degradable materials heavily depends on environmental conditions, such as temperature and fluid composition, as their primary degradation mechanism is hydrolysis. This variability necessitates the development of numerous grades with diverse dissolution properties to accommodate unique well conditions, complicating supply chains. A promising future direction involves exploring alternative dissolution mechanisms independent of well conditions, such as controlled explosions or self-sustained burning. Although still in their infancy, these technologies hold significant potential for revolutionizing the field. Another approach could be the use of advanced embedded catalyst systems that activate only under specific temperature and pH conditions to enhance controlled dissolution. This could be a feasible and promising route without significantly altering the polymer matrix system itself.

In summary, while substantial advancements have been made in dissolvable polymeric materials for the oil and gas industry, further research and innovation are essential to address current limitations, improve performance, and simplify supply chains. The continued collaboration between academia and industry will be pivotal in driving the next generation of materials and technologies.

## Figures and Tables

**Table 1:** Mapping from performance space to property space, and then to structure space.

Performance	High Strength	High Thermal Stability	Controllable Dissolvability
Property	a. High tensile strength b. High stiffness (compared with rubber) c. High flexural strength d. High flexural modulus	a. High Tg b. High Tm c. High HDT d. Delayed degradation	a. Proper water solubility b. Proper hydrolysis rate c. Proper thermal degradation rate d. Disintegration capability
Structure	Stiff polymer Stiff fibers Continuous fibers Long fibers Interfacial bonding	A rigid chain A high crystallinity Oriented crystals Stiff fibers/fillers Dissolvable coating	Water-soluble molecules Polymer chain containing hydrolysable linkages Hydrophilic chains Structural units sensitive to temperature Non-dissolvable particles in dissolvable matrix

**Table 2:** Rate constants and half time at a pH of 7 for the hydrolysis of some carboxylic acid esters at 25 °C.

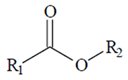 Rate Constants and Half-Lives at pH 7 for Hydrolysis of Some Carboxylic Acid Esters at 25 °C					
R_1_	R_2_	*K_A_* (M^−1^·s^−1^)	*K_N_* (s^−1^)	*K_B_* (M^−1^·s^−1^)	*T*_1/2_ (pH 7)
methyl	ethyl	1.1 × 10^−4^	1.5 × 10^−10^	0.11	2 year
methyl	*ter*-butyl	1.3 × 10^−4^		1.5 × 10^−3^	140 year
methyl	vinyl	1.4 × 10^−4^	1.1 × 10^−7^	10	7 day
methyl	phenyl	7.8 × 10^−5^	6.6 × 10^−8^	1.4	38 day
chloromethyl	methyl	8.5 × 10^−5^	2.1 × 10^−7^	140	14 h
dichloromethyl	methyl	2.3 × 10^−4^	1.5 × 10^−10^	2.8 × 10^3^	40 min
dichloromethyl	phenyl		1.8 × 10^−3^	1.3 × 10^4^	4 min

**Table 3:** Properties of Kuralon^®^ filament in comparison with other filament yarn [23].

	Kuralon			PET	Nylon 6	Aramid	Vectran
Type	1239	5501	5516-1			Regular	HT
Thickness (dtex)	1330	20,000	2000	1110	930	1670	1670
Number of filaments	200	1000	1000	250	96	1000	300
Tensile strength (cN/dtex)	8.2	9.8	11.9	8.1	8.1	19.4	22.9
Elongation at break (%)	7.7	6.6	6.4	10.7	19.4	3.9	3.8
Young’s modulus (cN/dtex)	177	203	260	110	34	493	530
Dry heat shrinkage (%)	0.8	0.6	0.4	11.2	6.5		
Boiling shrinkage (%)	4.5	2.5	2.2	5.4	11.8		
Specific gravity	1.30			1.38	1.14	1.41	1.44
Moisture regain (%)	5.0			0.4	4.5	7.0	0.0

**Table 4:** Mechanical properties of Kuredux^®^ monofilament from Kureha Corporation [24].

	Test Method	Unit	Measured Value
Tensile modulus	ISO 2062 [26]	GPa	29
Tensile strength		GPa	1.1
Tensile elongation		%	20
Single-end breaking force		GPa	1.1
Single-end breaking elongation		%	14

**Table 5:** Summarization of the properties of biodegradable aliphatic polyesters [31].

Sources	Biodegradable Aliphatic Polyester	*M_w_* ^(a)^ [gmol^−1^]	*T_m_* ^(b)^ [°C]	*T_g_* ^(c)^ [°C]	*T_s_* ^(d)^ [MPa]
Fossil fuel	Poly(glycolic acid) (PGA)	51,750	230	40	117
	Poly(ethylene succinate) (PES)	70,000	104	−10	23
	Poly(ε-caprolactone) (PCL)	80,921	65	−61	15
	Poly(propylene fumarate) (PPF)	-	-	23	-
Microbial fermentation	Poly(hydroxyalkanoate) (PHA)	-	166	29	40
	i. Poly(3-hydroxybutyrate) (PHB)	176,800	177	4	43
	i. Poly(3-hydroxybutyrate-co-3-hydroxyhexanoate) (PHBHHX)	500,000	130	-	20
	i. Poly(3-hydroxybutyrate-co-3-hydroxyvalerate) (PHBV)	163,000	170	-	-
	Poly(β-L-malic acid) (PLMA)	-	-	50	-
Plants	Poly(glycerol succinate) (PGSu)	-	-	−17	31
	Poly(glycerol sebacate) (PGS)	-	-	-	1
	Poly(butylene succcinate) (PBS)	88,400	114	−34	33
	Poly(lactic acid) (PLA)	-	173	63	70
	Poly(w-pentadecalactone) (PPDL)	150,200	104	-	29
	Poly(ethylene brassylate) (PEB)	-	70	30	-

^(a)^ molecular weight; ^(b)^ melting temperature; ^(c)^ glass-transition temperature; ^(d)^ tensile strength.

**Table 6:** Mechanical properties of PGA (Kuredux) from injection-molded tensile bars [40].

		Test Method	Unit	Measured Value
Specific Gravity		ISO 1183-1 [42]	-	1.50~1.60
Mechanical Properties				
Injection Molding	Tensile Modulus	ISO 527-1,2 [43,44]	GPa	7.4
	Tensile Strength	ISO 527-1,2	MPa	117
	Tensile Elongation	ISO 527-1,2	%	13
	Flexural Modulus	ISO 178 [45]	GPa	7.6
	Flexural Strength	ISO 178	MPa	195
	Charpy Impact Strength	ISO 179 [46] Notched	KJ/m^2^	2.2
	Izod Impact Strength	ISO 180 [47] Notched	KJ/m^2^	2.9
	Rockwell Hardness	ISO 2039-2 [48] M-scale	-	111

**Table 7:** Effect of the HNBR content on the mechanical properties of MPUR/HNBR composites [76].

Properties	M(MPUR)/m(HNBR)				
	100/0	80/20	70/30	60/40	0/100
Tensile Strength/MPa	27.3	29.0	26.4	24.0	21.9
100% modulus/MPa	11.3	15.3	15.1	14.2	6.8
Elongation at break/%	220	232	199	194	385
Tear strength/(kN·m^−1^)	28.8	35.9	35.5	32.0	71.0
Hardness/Shore A	89	93	91	92	82

## Data Availability

No new data were created or analyzed in this study. Data sharing is not applicable to this article.
